# Natural Apocarotenoids and Their Synthetic Glycopeptide Conjugates Inhibit SARS-CoV-2 Replication

**DOI:** 10.3390/ph14111111

**Published:** 2021-10-30

**Authors:** Ilona Bereczki, Henrietta Papp, Anett Kuczmog, Mónika Madai, Veronika Nagy, Attila Agócs, Gyula Batta, Márton Milánkovits, Eszter Ostorházi, Ana Mitrović, Janko Kos, Áron Zsigmond, István Hajdú, Zsolt Lőrincz, Dávid Bajusz, György Miklós Keserű, Jan Hodek, Jan Weber, Ferenc Jakab, Pál Herczegh, Anikó Borbás

**Affiliations:** 1 Department of Pharmaceutical Chemistry, University of Debrecen, Egyetem tér 1, H-4032 Debrecen, Hungary; bereczki.ilona@pharm.unideb.hu; 2 Szentágothai Research Centre, National Laboratory of Virology, Ifjúság útja 20, H-7624 Pécs, Hungary; pappheni@gamma.ttk.pte.hu (H.P.); kuczmog@hotmail.com (A.K.); madai.monika@pte.hu (M.M.); jakab.ferenc@pte.hu (F.J.); 3 Institute of Biology, Faculty of Sciences, University of Pécs, Ifjúság útja 6, H-7624 Pécs, Hungary; 4 Department of Biochemistry and Medical Chemistry, Medical School, University of Pécs, Szigeti u. 12, H-7624 Pécs, Hungary; vera.nagy@aok.pte.hu (V.N.); attila.agocs@aok.pte.hu (A.A.); 5 Department of Organic Chemistry, University of Debrecen, H-4032 Debrecen, Hungary; batta.gyula@science.unideb.hu; 6 Department of Medical Microbiology, Semmelweis University, Nagyvárad tér 4, H-1089 Budapest, Hungary; milankovits.marton@gmail.com (M.M.); ostorhazi.eszter@med.semmelweis-univ.hu (E.O.); 7 Department of Biotechnology, Jožef Stefan Institute, Jamova 39, 1000 Ljubljana, Slovenia; ana.mitrovic@ijs.si (A.M.); janko.kos@ffa.uni-lj.si (J.K.); 8 Faculty of Pharmacy, University of Ljubljana, Aškerčeva cesta 7, 1000 Ljubljana, Slovenia; 9 TargetEx Ltd., Madách Imre Utca 31/2, H-2120 Dunakeszi, Hungary; zsigmond@target-ex.com (Á.Z.); hajdu@target-ex.com (I.H.); lorincz@target-ex.com (Z.L.); 10 Research Centre for Natural Sciences, Medicinal Chemistry Research Group, Magyar Tudósok krt. 2, H-1117 Budapest, Hungary; bajusz.david@ttk.hu (D.B.); keseru.gyorgy@ttk.hu (G.M.K.); 11 Institute of Organic Chemistry and Biochemistry, Czech Academy of Sciences, Flemingovo nam. 2, CZ-16000 Prague, Czech Republic; jan.hodek@uochb.cas.cz (J.H.); jan.weber@uochb.cas.cz (J.W.)

**Keywords:** teicoplanin, ristocetin, bixin, crocetin, β-apocarotenoic acid, SARS-CoV-2, antiviral activity, antibacterial activity

## Abstract

The protracted global COVID-19 pandemic urges the development of new drugs against the causative agent SARS-CoV-2. The clinically used glycopeptide antibiotic, teicoplanin, emerged as a potential antiviral, and its efficacy was improved with lipophilic modifications. This prompted us to prepare new lipophilic apocarotenoid conjugates of teicoplanin, its pseudoaglycone and the related ristocetin aglycone. Their antiviral effect was tested against SARS-CoV-2 in Vero E6 cells, using a cell viability assay and quantitative PCR of the viral RNA, confirming their micromolar inhibitory activity against viral replication. Interestingly, two of the parent apocarotenoids, bixin and β-apo-8′carotenoic acid, exerted remarkable anti-SARS-CoV-2 activity. Mechanistic studies involved cathepsin L and B, as well as the main protease 3CLPro, and the results were rationalized by computational studies. Glycopeptide conjugates show dual inhibitory action, while apocarotenoids have mostly cathepsin B and L affinity. Since teicoplanin is a marketed antibiotic and the natural bixin is an approved, cheap and widely used red colorant food additive, these readily available compounds and their conjugates as potential antivirals are worthy of further exploration.

## 1. Introduction

The still ongoing COVID-19 pandemic imposes a significant public health burden and a growing economic threat worldwide, and urges to develop effective new antivirals against the causative agent, severe acute respiratory syndrome coronavirus 2 (SARS-CoV-2). Several vaccines have been developed to prevent infection, which are showing positive results. However, vaccine scepticism and the emergence of new virus variants, which could avoid immune responses elicited by either vaccines or previous infections [1,2], still make the development of prophylactic and therapeutic agents against SARS-CoV-2 infection of paramount importance. Early responding reposition campaigns [3,4] identified multiple drug candidates; however, only remdesivir, a broad-spectrum antiviral drug for intravenous use, has been approved for the treatment of COVID-19 patients. The only oral alternative was favipiravir, another broad-spectrum antiviral agent originally developed against influenza [5], which was approved for emergency application in the treatment of mild COVID-19 cases in several countries. Unfortunately, neither of the repositioned antivirals were universally effective [6] and both have adverse effects [7]. Therefore, it is imperative to develop specific and safe antiviral drugs against SARS-CoV-2. The global research community has launched diverse de novo efforts, including worldwide collaborations such as the COVID Moonshot consortium for the discovery of SARS-CoV-2 main protease inhibitors [8], and large fragment screens against multiple viral targets [9,10] using innovative compound libraries [11,12]. In addition to bottom-up approaches, existing chemical entities can be further exploited by repurposing approved drugs [13,14], clinical trial drug candidates or natural products.

**Figure 1:**
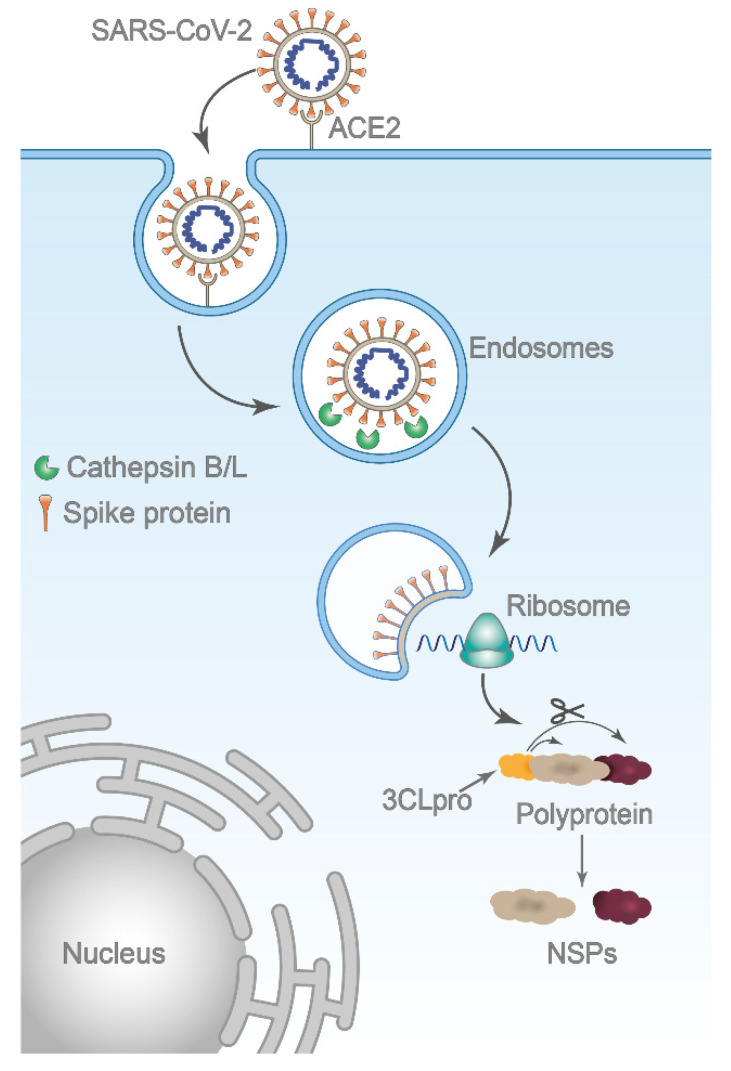
Schematic representation of SARS-CoV-2 entry by the endosomal route. After binding to its host receptor angiotensin-converting enzyme 2 (ACE2), the virus is internalized via endocytosis. The low pH within the endosome activates Cat L, which in turn triggers membrane fusion by proteolytic cleavage of the S protein. Upon membrane fusion, viral genomic RNA is released in the cytoplasm and initiates translation of polyproteins, which is, subsequently, cleaved into nonstructural proteins (NSPs) by viral main protease 3CLpro. Several NSPs constitute the replicase–transcriptase complex essential for viral replication.

Cathepsin L (Cat L), an endosomal cysteine protease is considered a promising target for the development of anti-COVID-19 drugs, as it plays a key role in SARS-CoV-2 infection [15,16,17]. SARS-CoV-2 employs a three-step method for membrane fusion, involving receptor binding and induced conformational changes in surface spike (S) glycoprotein, followed by viral entry through the plasma membrane (early pathway) or by endocytosis (late pathway); the cell entry is mediated by proteolytic cleavage of the S protein by host proteases, the type 2 transmembrane serine protease (TMPRSS2) or cathepsins [17,18]. TMPRSS2 triggers the fusion at the plasma membrane, but if membrane bound proteases are not available, the virus is endocytosed and the acidic milieu activates cathepsin L to trigger fusion at the endosomal membrane [17,18,19]. In the endosomal pathway, the intra-lysosomal activation of the spike protein by cathepsin L is critical for the release of the coronavirus genome into human host cells (Figure 1). Recently, Zhao and co-workers demonstrated the crucial role of Cat L in patients with COVID-19 [20]. Their study revealed that SARS-CoV-2 infection promoted Cat L expression and enzyme activity, which, in turn, enhanced viral infection. Moreover, it was also demonstrated that the inhibition of Cat L activity prevented SARS-CoV-2 infection both in vitro and in vivo.

Glycopeptide antibiotics, such as teicoplanin, dalbavancin, oritavancin and telavancin, have been found to prevent the host cell entry process of the Ebola virus, Middle East respiratory syndrome coronavirus (MERS-CoV) and severe acute respiratory syndrome coronavirus (SARS-CoV) by inhibiting the Cat L activity in a dose-dependent manner [21]. A recent study by Zhang et al. proposed that teicoplanin could block SARS-CoV-2 pseudovirus infection in A549 cells, HEK293 T cells and Huh7 cells [22]. Teicoplanin is used routinely in the clinic for the treatment of severe infections by multiresistant Gram-positive pathogens, especially methicillin-resistant *Staphylococcus aureus* (MRSA). Thus, teicoplanin was proposed as a potential dual drug to block SARS-CoV-2 infection at the early stage and to prevent possible *Staphylococcus aureus* superinfection [22,23]. Ceccarelli et al. studied the impact of teicoplanin on the course of COVID-19 in critically ill patients [24]. The incidence of Gram-positive infection was promisingly low, but no significant antiviral effect was observed. The authors concluded that the anti-SARS-CoV-2 activity of teicoplanin could be more effective at early infections, which is in accordance with recent results by Zhao et al. [20].

We, and others, have demonstrated that the antiviral spectrum and efficacy of glycopeptide antibiotics can be increased by lipophilic modifications, and a number of semisynthetic lipophilic derivatives of the antibiotics vancomycin, teicoplanin and ristocetin have been published that showed good antiviral activity against coronaviruses [25,26,27], as well as HIV [28], flaviviruses [29] and influenza viruses [26,27,30,31,32,33,34]. Unfortunately, the high antiviral activity is often associated with cytotoxicity [33,34].

In this work, we report on the use of apocarotenoids for the lipophilic modification of glycopeptide antibiotics as potential new antivirals against SARS-CoV-2. Selecting non-cytotoxic apocarotenoids, we hypothesize that these derivatives possess anti-SARS-CoV-2 activity without cytotoxicity.

Carotenoids are natural hydrophobic products with 40 carbon atoms, having conjugated polyenic structures and possessing a number of beneficial biological activities, including antioxidant and immune enhancer properties, the regression of malignant lesions and mutagenesis inhibition [35,36,37]. Carotenoids are susceptible to oxidation; they are readily metabolised to apocarotenoids by oxygenases or reactive oxygen species. Apocarotenoids (with less than 40 carbons), generally bearing aldehyde or carboxylic acid functions, are believed to be responsible for the beneficial health effect of nutritional carotenoids [38]. Bixin (main source is *Bixa orellana*) and crocetin (main source is saffron) are naturally occurring apocarotenoids-bearing carboxyl groups. Both are used as powerful, biocompatible and non-toxic colorants and food additives, having favourable physiological activities [39,40]. The ethyl ester of β-apo-8′-carotenoic acid is mostly produced synthetically and can be used for food colouring as well. In our work, the readily available apocarotenoid carboxylic acids are used for the acylation of the primary amino functionality of glycopeptide antibiotic derivatives.

## 2. Results

### 2.1. Chemical Synthesis

**Figure 2:**
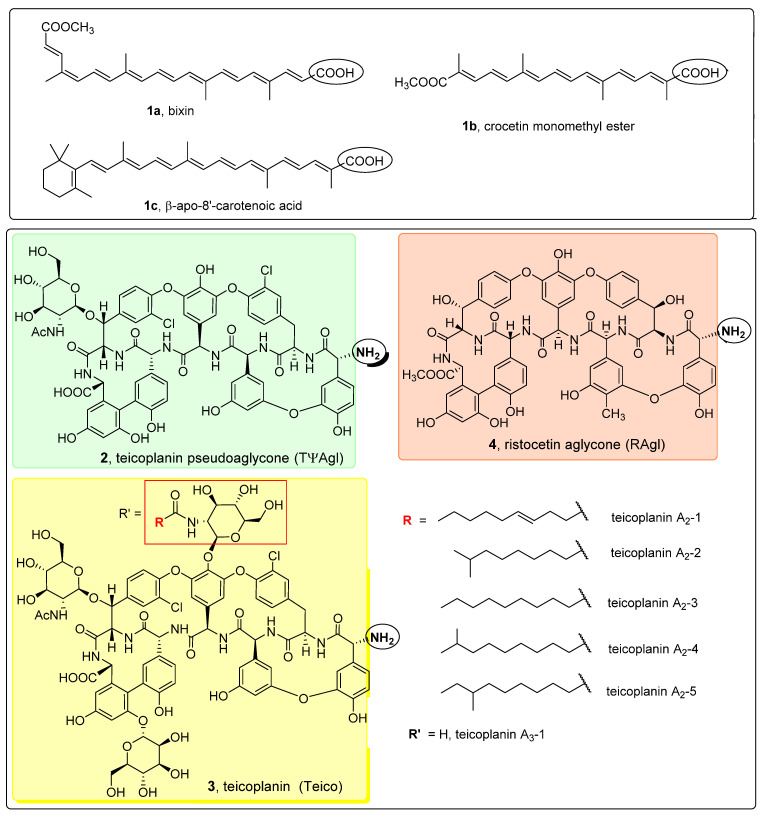
Apocarotenoids (top panel) and glycopeptide antibiotic derivatives (bottom panel) used for the synthesis of the glycopeptide conjugates. The functional groups involved in the conjugation reactions are marked with a circle.

Apocarotenoids and glycopeptide antibiotic derivatives used in this study for the synthesis of glycopeptide conjugates are shown in Figure 2. Bixin (**1a**) was isolated from Bixa orellana seed extract (this extract is also known as annatto), crocetin monomethyl ester (**1b**) was prepared from crocetin in two steps via diesterification followed by partial ester cleavage and β-apo-8′-carotenoic acid (**1c**) was obtained from a commercially available ethyl ester derivative by saponification. Teicoplanin pseudoaglycone (**2**) was prepared from teicoplanin (**3**) by partial deglycosylation [31], while ristocetin aglycone (**4**) was obtained by the complete removal of sugar components of the parent antibiotic [32].

**Figure 3:**
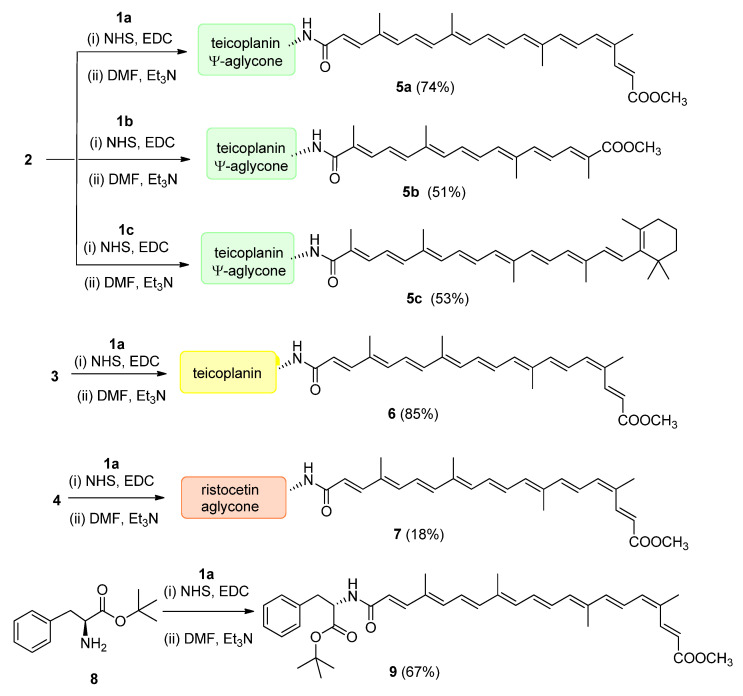
Conjugation of apocarotenoids **1a**–**c** to the *N*-terminal amino group of glycopeptide derivatives **2**–**4** and to l-phenylalanine derivative **8**. (NHS: *N*-hydroxysuccinimide; EDC: 1-ethyl-3-(3-dimethylaminopropyl)carbodiimide).

From bixin (**1a**), crocetin monomethyl ester (**1b**) and β-apo-8′-carotenoic acid (**1c**) *N*-hydroxysuccinimide active esters were prepared using 1-ethyl-3-(3-dimethylaminopropyl)carbodiimide (EDC). The *N*-terminal primary amino group of teicoplanin pseudoaglycone (**2**) was acylated by these active esters to produce apocarotenoid derivatives **5a** (74%), **5b** (49%) and **5c** (53%), respectively (Figure 3).

Therapeutically used natural teicoplanin **3** (also known as the teicoplanin complex) is a complex mixture that comprises five major components having different lipophilic (C10-C11) *N*-acyl substituents at one of the glucosamine residues. In order to study the effect of the original sugar substituents (*N*-acyl glucosamine, d-mannose) of teicoplanin on the biological activity, bixin was conjugated to teicoplanin complex **3,** resulting in the expected **6** in a high yield. To test the impact of the peptide core on potential antiviral activity, compound **7** was synthesized by conjugating bixin to ristocetin aglycone **4**. In addition, a simplified model of the glycopeptide–bixin conjugate was prepared as a reference compound by acylating phenylalanine *t*-butyl ester **8** with the active ester of bixin **1a**.

### 2.2. Antiviral Evaluations

The anti-SARS-CoV-2 activity of all new glycopeptide–apocarotenoid conjugates (**5a**–**c**, **6**, and **7**) were evaluated using three orthogonal methods, including the viral RNA reduction assay, a cytopathic effect (CPE) reduction assay and an immunofluorescence assay. The parent glycopeptide cores (**2**–**4**), bixinoil-phenylalanine **9** and apocarotenoids **1a**–**c** were included in the antiviral studies as reference compounds (Table 1).

First, a viral RNA reduction assay was used to determine antiviral EC_50_ values in Vero E6 cells infected with SARS-CoV-2 at a multiplicity of infection (MOI) of 0.01 (Figure S13). Apocarotenoid conjugates of teicoplanin pseudoaglycone and ristocetin aglycone (**5a**–**c**, and **7**), as well as native teicoplanin itself (**3**), showed a very similar antiviral effect with EC_50_ values of 4.4–6.7 μM; among these, the apocarotenoic acid conjugate **5c** showed the highest, while the ristocetin aglycone **7** derivative showed the lowest activity. Using this antiviral assay, the bixin conjugate of teicoplanin (**6**) exhibited the most robust antiviral effect against SARS-CoV-2 with an EC_50_ value of 1.8 μM.

The aglycone derivatives **2** and **4** proved to be completely inactive. This finding was not unexpected, since, in contrast to lipophilic glycopeptide antibiotics (e.g., teicoplanin, dalbavancin, oritavancin and telavancin), analogues with no lipophilic groups (e.g., vancomycin) had no antiviral activity either [21]. Hence, our results, in line with literature results, suggested that lipophilic groups play an important role in the antiviral mechanism of action of glycopeptide derivatives [21,22].

Surprisingly, two apocarotenoids, bixin **1a** and β-apo-8′-carotenoic acid **1c**, also exerted remarkable activity against SARS-CoV-2, while crocetin-monomethyl ester **1b** did not show antiviral activity at a 50 μM concentration.

Based on the reasonable anti-SARS-CoV-2 effect of bixin, the commercially available annatto (composed of bixin and norbixin ~4:1) and norbixin (**1d**), the minor component of annatto, were also tested against SARS-CoV-2. The activity of annatto (EC_50_ = 9.2 μM) was in good correlation with its bixin content, while norbixin was completely inactive.

Next, antiviral EC_50_ values were determined in Vero E6 cells infected with SARS-CoV-2 at an MOI of 0.04 using a CPE-based assay (Figure S14). The activity of the glycopeptide conjugates followed similar trends, but the EC_50_ values were one order of magnitude higher, which could be explained by the much higher level of infection in addition to the differences in methods. In this assay, the native teicoplanin **3** showed the highest efficacy with an EC_50_ value of 15.7 μM. The remarkable antiviral activity of apocarotenoids, bixin **1a** and β-apo-8′-carotenoic acid **1c** alone against SARS-CoV-2 was further confirmed by this method. As the crocetin-monomethyl ester proved to be inactive in the previous assay, it was not tested.

Finally, an immunofluorescence assay (IFA) was also used to evaluate anti-SARS-CoV-2 activity of the apocarotenoids and their teicoplanin pseudoaglycone conjugates (Figures S15 and S16). The activity of teicoplanin (**3**), teicoplanin derivatives (**5a**–**5c**) and apocarotenoids (**1a** and **1c**) in inhibiting viral replication was further confirmed by the visualization of SARS-CoV-2 nucleoprotein expression via fluorescence microscopy at 72 h post-infection.

### 2.3. Mechanism of Action

Based on the mechanism described for teicoplanin, we hypothesized that our compounds exerted their antiviral effect through inhibiting the enzyme activity of cathepsin L. To test this hypothesis, the Cat L inhibitory effect of the compounds showing antiviral activity (except for ristocetin derivative **7**) was assayed (Figure S17). The new semisynthetic glycopeptides (**5a**–**5c**, **6**) and the apocarotenoids (**1a**, **1c** and **9**) displayed inhibitory activity with IC_50_ values of 22–103 µM (Table 2). These data showed that the compounds inhibited Cat L at one order of magnitude higher concentrations (in the case of the viral reduction assay at an MOI of 0.01) or at the same concentration range (in the case of the CPE-based and IF assays at MOI of 0.04) as they inhibited SARS-CoV-2.

Importantly, teicoplanin exerted a very low inhibitory activity against Cat L (5% inhibition at 50 µM), which seemingly contradicted the results of Zhou et al. and Zhang et al., reporting that teicoplanin blocks the SARS-CoV-1 and SARS-CoV-2 entry with IC_50_ values of 3.76 and 1.66 µM, respectively, by specifically inhibiting Cat L in the late endosome/lysosome [21,22]. At the same time, the IC_50_ values for Cat L inhibition, determined by two different methods [41,42] by Zhou et al., were 208 µM and 425 µM [21]. The authors explained this huge difference in entry-blocking and enzyme inhibitory activities by the relatively low sensitivity of the cathepsin L activity assays [21]. However, our results clearly showed that while the antiviral potency of the new semisynthetic compounds was very similar to the one of teicoplanin, the Cat L inhibitory potency of teicoplanin was much inferior than that of our compounds.

Looking for alternative mechanisms, we examined the effect of the compounds on the exopeptidase activity of cathepsin B (Cat B) as a potential host target (Figure S18), as well as to 3-chymotrypsin-like protease (3CLPro, also called main protease, MPro) as a potential viral target (Figure S19).

The glycopeptide derivatives **5a**–**5c** and **6**, as well as PheAla-bixin **9**, inhibited Cat B exopeptidase to a similar extent as Cat L. Apocarotenoids, bixin **1a** and β-apo-8′-carotenoic acid **1c** showed a significantly higher inhibitory activity against Cat B than against Cat L. The activation of the viral S protein by lysosomal Cat L has been shown to be critical for SARS-CoV-1 entry through endocytosis [42]. The proteolysis of viral glycoprotein by both Cat B and Cat L is required for the membrane fusion of the Ebola virus [43,44]. Hoffmann et al. reported that either Cat L or Cat B play an important role in the proteolytic priming of the S protein of SARS-CoV-2 as well [18]. Ou et al. studied the effect of specific inhibitors of Cat L and Cat B on SARS-CoV-2 entry, and this study revealed that Cat L, rather than Cat B, is essential for S protein priming and membrane fusion in the lysosome [19]. The crucial role of Cat L in SARS-CoV-2 infection was recently confirmed by Zhao et al. [20]. Therefore, it is likely that cathepsin L inhibition plays a key role in the anti-SARS-CoV-2 activity of apocarotenoids and their teicoplanin conjugates. At the same time, given the general role of cathepsins in the entry of different viruses into cells, activity against both cathepsins L and B suggests a broad antiviral potency [16,45].

Viral main protease 3CLPro is essential for the cleavage of viral polyproteins and its difference from cellular proteases makes this enzyme an ideal drug target against SARS-CoV-2. Importantly, Tripathy et al. demonstrated through an SPR-based method that teicoplanin can significantly reduce the proteolytic activity of 3CLPro [46], suggesting that teicoplanin may be a dual-mechanism anti-SARS-CoV-2 agent. To test this hypothesis, 3CLPro inhibition was assayed in a FRET-based inhibition measurement (Figure S19).

The main protease 3CLPro was blocked by only four compounds; norbixin, β-apo-8′-carotenoic acid **1c**, and two teicoplanin pseudoaglycone conjugates, **5a** and **5c**, showed dose-dependent inhibitory activities (Table 2). It is interesting that, while the glycopeptide core was not active on its own, it seemed to enhance the activity of the respective apocarotenoids upon conjugation, suggesting that both units are utilized upon 3CLPro binding and inhibition.

**Figure 4:**
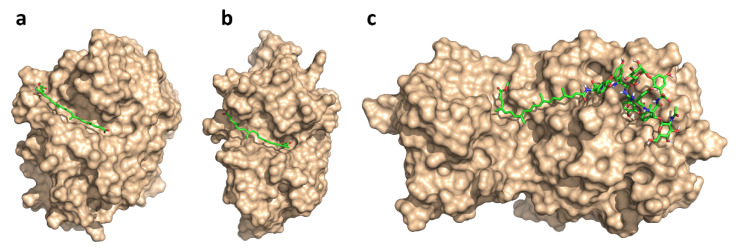
Predicted binding modes of bixin **1a** for cathepsins B (**a**) and L (**b**), and teicoplanin pseudoaglycone conjugate **5a** for 3CLPro (**c**).

To understand the preference of the glycopeptide conjugates and apocarotenoids towards certain proteases, we predicted their respective binding modes by computational methods. Specifically, due to the large size and conformational complexity of these compounds, we applied low-mode docking [47]. This approach used the LMOD conformational searching algorithm [48], which samples the conformational space effectively along low-frequency vibrational modes. It, thus, provided a computationally efficient way to account for the flexibility of large ligands, and binding site residues, or even entire protein loops during ligand docking. We performed low-mode docking to model the binding modes of the most potent compounds against each of the protease targets: these were bixin **1a** for cathepsins B and L, and the teicoplanin pseudoaglycone conjugate **5a** for the main protease. The low-energy binding modes presented in Figure 4 highlight the crucial differences between the binding sites of the enzymes. For cathepsins, the subsites of the active site were arranged almost linearly, in channel-shaped binding cavities; these were nicely complemented by the long apocarotenoid chains, with the polar end-groups serving as anchor points against H-bond-donating residues, such as G74 and H111 (CatB) or N18 and G164 (CatL). By contrast, in the main protease of SARS-CoV-2, the crucial S1, S2 and S1’ subsites were arranged in a less accessible, cavern-like structure. Here, the numerous polar features of the glycopeptide core provided H-bonding interactions against the neighbouring active site residues. The apocarotenoid chain extended from subsite S1, along the protein surface, into a shallow groove between domains I and II of the main protease [49]. Based on these observations, we proposed that binding to cathepsins B and L was mediated primarily by the apocarotenoid chains, while there were key contributions from both main structural units in binding to the main protease. Due to the lack of more substantial, biophysical proof for on-target engagement (e.g., X-ray structures or direct binding measurements), these models provide a possible explanation for the observed target preferences.

### 2.4. Antibacterial Evaluation

In patients with COVID-19, methicillin-resistant *Staphylococcus aureus* (MRSA) and enterococci strains should be considered for superinfection [24]. Therefore, we tested the compounds by the broth microdilution method on a panel of eight Gram-positive bacterial strains. The lipophilic modification of teicoplanin derivatives **5a**–**5c** and **6** did not led to a dramatic change in the antibacterial activity compared to the parent antibiotic teicoplanin. The ristocetin aglycone derivative **7** showed significantly lower effects against all bacteria tested than teicoplanin. Against some of these relevant strains, the crocetin and apocarotenoic acid conjugates of teicoplanin pseudoaglycone (**5b** and **5c**) showed similar or better activity than teicoplanin. The teicoplanin–bixin conjugate **6** was less effective against the MRSA strain than native teicoplanin, but showed significant activity against the two vancomycin-resistant enterococci. As anticipated, neither the apocarotenoids, nor phenylalanoyl-bixin **9** exhibited activity against any bacterium (Table 3).

## 3. Discussion

Literature data suggested that the glycopeptide antibiotic teicoplanin may be used as a dual action drug in the management of COVID-19; its anti-SARS-CoV-2 pseudovirus activity was proved and it has the potential to prevent possible *Staphylococcus aureus* superinfection [21,22,23]. Our previous results demonstrated that the antiviral activity of semisynthetic lipophilic glycopeptides, especially teicoplanin pseudoaglycone derivatives, often outweighed the activity of teicoplanin, while retaining their antibacterial activity [31,32,33,34]. This prompted us to prepare new lipophilic conjugates of teicoplanin pseudoaglycone as potential antivirals against SARS-CoV-2. Three apocarotenoids, bixin, crocetin and β-apo-8′-carotenoic acid, were chosen as lipophilic moieties for two reasons: (i) they are non-toxic and (ii) their carboxylic acid function allows easy conjugation to the amino group of glycopeptides. Lipophilic apocarotenoid glycopeptide conjugates of teicoplanin (**6**), its pseudoaglycone (**5a**–**5c**), and a related ristocetin aglycone (**7**), were prepared and tested against SARS-CoV-2 infected cells. Phenylalanoyl-bixin **9** was also prepared and used as a reference in biological studies. The antiviral activity of the semisynthetic derivatives as well as the starting glycopeptides and apocarotenoids was evaluated in Vero E6 cells using three orthogonal assays. The semisynthetic glycopeptides and native teicoplanin inhibited the viral reproduction with similar efficacy, the EC_50_ values were in the low micromolar range in the RNA reduction assay and in the ten-micromolar range in the CPE-based and immunofluorescent assays. The one order of magnitude difference between the EC_50_ values can be explained by the four times lower multiplicity of infection and the removal of the viral inoculum after a half hour incubation in the RNA reduction assay. The most interesting finding of this study was the remarkable, unexpected antiviral activity of two apocarotenoids, bixin (**1a**), and β-apo-8′-carotenoic acid (**1c**). Although a plethora of natural compounds has been investigated and listed as potential SARS-CoV-2 inhibitors [50,51,52], apocarotenoids have not been among the candidates so far.

Zhou et al. hypothesized that teicoplanin might interact with the enzymatic domains of cathepsin L and block their functions similar to the reported inhibitory effect of antimicrobial peptide LL-37 on cathepsin L [21,53]. This hypothesis was based on the lipid II binding ability of teicoplanin, but notably, this was not supported by our results presented here, as teicoplanin and its pseudoaglycone alone (without the conjugated apocarotenoid chains) were not observed to significantly inhibit either of the cathepsins, or 3CLPro. This was in line with our computational results obtained by low-mode docking, an efficient implementation of the flexible docking paradigm; for both cathepsins suggested that binding is mainly achieved by the apocarotenoid chains. In the case of 3CLPro, both structural units have importance, although the enzyme inhibition results highlighted the presence of the apocarotenoid chain as a stronger requirement. Altogether, our results suggested that this series of glycopeptide apocarotenoid conjugates may have a complex antiviral mechanism of action as they acted against both human and viral proteases. However, regarding the modest enzyme inhibitory activity, other mechanisms may also contribute to their antiviral effect.

Together with the demonstrated antibacterial properties, these compounds can be promising candidates against SARS-CoV-2 infection, especially in groups with a higher risk of bacterial superinfection. This was further corroborated by the fact that both of the constituting structural units are in human use: teicoplanin is a marketed drug, while apocarotenoids are approved natural colorants used as food additives.

## 4. Materials and Methods

### 4.1. Chemical Synthesis

#### 4.1.1. General Information

*tert*-Butyl l-phenylalaninate **8** was synthesized according to the literature [54]. TLC was performed on Kieselgel 60 F254 (Merck) with detection either by immersing into ammonium molybdate-sulfuric acid solution followed by heating or by using Pauly’s reagent for detection. Flash column chromatography was performed using Silica gel 60 (Merck 0.040–0.063 mm).

Conventional 1D and 2D ^1^H and ^13^C NMR spectra (^1^H-COSY, ^1^H-^13^C-HSQC, ^1^H-^13^C-HSQC-TOCSY, ^1^H-^13^C-HMBC) were recorded using a Bruker DRX-400 spectrometer (at 298 K or 300 K) and a 500 MHz (Bruker, Billerica, MA, USA), Avance II spectrometer (at 310 K) equipped with a TXI probe head. Chemical shifts were referenced to Me_4_Si (0.00 ppm for ^1^H) and to the solvent residual signals (CDCl_3_ or DMSO-d_6_). For the glycopeptide derivatives (**5a**, **5b**, **5c**, **7**), initial signal assignments were aided using earlier works on glycopeptide aglycon NMR characterisations [55,56] unlabelled peaks in the 6–8/110–145 ppm ^1^H/^13^C regions belonged to carotenoid sidechain atoms. Typical 90° pulses were 10 and 16 μs for the ^1^H and ^13^C channels, and relaxation delays allowed were generally 2 s. The ^1^H-^13^C Heteronuclear Single-Quantum Correlation (HSQC) spectra were recorded using «hsqcetgpsi2» pulse program and 4–8 scans for each of the 512 increments in indirect dimension. The 2D spectra were processed with Topspin 3.1 software using Gaussian window function (Lb = −5, GB = 0.05) in F2 and cosine-square (QSINE, SSB = 2) in F1 dimension. To support the ^1^H/^13^C assignments heteronuclear multiple bond correlation experiments, HMBC (pulse program: “hmbcgplpndqf”, 70 ms evolution time), HSQC-TOCSY (pulse program: “hsqcdietgpsi”, 70 ms mixing time) and homonuclear correlated spectroscopy (COSY) (pulse program: “cosygpqf”) were also run. The digital resolution of the processed spectra was typically 2–3 Hz. The ^13^C J-modulated spin-echo and ^1^H-^13^C HSQC spectra of glycopeptide derivatives (**5a**, **5b**, **5c**, and **7**) are shown in the Supplementary Material, equipped with characteristic ^1^H-^13^C signal assignments.

MALDI-TOF MS measurements were carried out with a Bruker Autoflex Speed mass spectrometer equipped with a time-of-flight (TOF) mass analyser. In all cases, 19 kV (ion source voltage 1) and 16.65 kV (ion source voltage 2) were used. For reflectron mode, 21 kV and 9.55 kV were applied as reflector voltage 1 and reflector voltage 2, respectively. A solid phase laser (355 nm, ≥100 μJ/pulse), operating at 500 Hz, was applied to produce laser desorption and 3000 shots were summed. 2,5-Dihydroxybenzoic acid (DHB) was used as matrix and F_3_CCOONa as cationising agent in DMF. ESI-QTOF MS measurements were carried out on a maXis II UHR ESI-QTOF MS instrument (Bruker), in positive ionization mode. The following parameters were applied for the electrospray ion source: capillary voltage: 3.5 kV; end plate offset: 500 V; nebulizer pressure: 0.8 bar; dry gas temperature: 200 °C; dry gas flow rate: 4.5 L/min. Constant background correction was applied for each spectrum; the background was recorded before each sample by injecting the blank sample matrix (solvent). Na-formate calibrant was injected after each sample, which enabled internal calibration during data evaluation. Mass spectra were recorded by otofControl version 4.1 (build: 3.5, Bruker) and processed by Compass DataAnalysis version 4.4 (build: 200.55.2969).

For analytical RP-HPLC, a Waters 2695 Separations Module (Waters Corp., Milford, CT, USA) was used. The separation was carried out on a VDSpher PUR 100 C18-M-SE, 5 μm, 150 × 4.6 mm column at an injection volume of 10 μL, using a flow rate of 1.0 mL/min with a Waters 2996 DAD set at 254 nm and a Bruker MicroTOF-Q type Qq-TOF MS instrument (Bruker Daltonik, Bremen, Germany) as detectors. The following system was used for the elution: Solvent A: Water:MeCN 9:1 + 0.005 *v*/*v*% TFA; Solvent B: MeCN. Gradient elution: from 20% of B to 80% from 0 to 40 min, 80% of B from 40 to 50 min and 20% of B from 51 min in the case of glycopeptide derivatives **5a**–**c**, **6** and **7** and from 20% of B to 80% from 0 to 40 min, 80% of B from 40 to 60 min and 20% of B from 61 min in the case of **9**. The MicroTOF-Q mass spectrometer was equipped with an electrospray ion source. The mass spectrometer was operated in positive ion mode with a capillary voltage of 3.5 kV, an endplate offset of −500 V, nebulizer pressure of 1.8 bar and N_2_ as drying gas with a flow rate of 9.0 L/min at 200 °C. The mass spectra were recorded by means of a digitizer at a sampling rate of 2 GHz. The mass spectra were calibrated externally using the exact masses of clusters [(NaTFA)_n_ + TFA]^+^ from the solution of sodium trifluoroacetate (NaTFA). The spectra were evaluated with the DataAnalysis 3.4 software from Bruker. HPLC-DAD measurements were conducted with gradient pump Dionex P680 and a diode array detector Dionex PDA-100 (Thermo Fisher Scientific Inc., Waltham, MA, USA). Chromatograms were detected at 450 nm wavelength; the data acquisition was performed by Chromeleon 6.8 or 7.3 software. The separation was carried out on an endcapped C18 column (250 × 4.6 mm i.d.; LiChrospher C18, 5 µm, Merck Europe KGaA, Darmstadt, Germany).

#### 4.1.2. Isolation of Bixin (**1a**)

A total of 500 mg of *Bixa orellana* seed extract (INEXA C.A., Ecuador) was flash chromatographed on a 100 g silica gel column in dichlomethane/methanol 10:1. The fractions containing the main reddish violet pigment were collected and evaporated. The crude bixin was crystallized from dichloromethane/hexane to yield 170 mg of bixin **1a** (m.p. 198 °C). Physical and chromatographic properties were identical with those of our authentic sample and literature data [57].

#### 4.1.3. Synthesis of Crocetin Monomethyl Ester (**1b**)

Crocetin dimethyl ester (356 mg, 1 mmol) was dissolved in THF (5 mL) and NaOH (500 mg, 12.5 mmol) was added in methanol (16 mL). The solution was stirred for a week and then 5% citric acid (50 mL) was added. The reaction mixture was washed with dichloromethane (3 × 50 mL), the organic phase was dried and evaporated. The product was purified by column chromatography (dichloromethane/methanol 9:1) to yield crocetin monomethyl ester **1b** [58] (120 mg, 35%, purity: 97%, HPLC) as an orange powder and the starting material (72 mg). M.p. 204–205 °C. UV (ethanol) 404, 427, 452 nm. HRMS (MALDI): *m*/*z* calcd for C_21_H_26_O_4_+Na^+^: 365.1723 [*M*+Na]^+^; found: 365.1719.

#### 4.1.4. Synthesis of β-Apo-8′-carotenoic Acid (**1c**)

Ethyl apo-8′-carotenoate (Hoffmann-La Roche, now DSM) (500 mg, 1.086 mmol) was dissolved in diethyl ether (200 mL) and saponified overnight with 30% methanolic KOH solution (30 mL). On the next day, 5% citric acid (300 mL) and diethyl ether (100 mL) were added to the reaction mixture and the phases were separated. The ethereal phase was washed twice with brine, dried and evaporated. The crude acid was crystallized from toluene/hexane to yield **1c** (322 mg, 69%, HPLC purity: 100%) as a dark red product. M.p. 194–195 C. UV (ethanol) 443, 463 nm. MS (MALDI-TOF): *m/z* calcd for C_30_H_40_O_2_+H^+^: 433 [M+H]^+^; found: 433.

#### 4.1.5. Synthesis of Norbixin (**1d**)

In total, 1.5 M aqueous tetrabutylammonium hydroxide solution (Acros, purum) (2.8 mL, 4.2 mmol) was added to a stirred (partial) solution of bixin (336 mg, 0.85 mmol) in THF (18 mL). The mixture was stirred at RT for 35 min (the reaction was monitored by TLC analysis). Then, the clear dark-red solution was quenched with 1 M aqueous acetic acid solution (10 mL, 10 mmol). The solution was seeded and stirred at RT for 15 min while the product began to crystallize. Water (25 mL) was added dropwise over 30 min to the stirred suspension. The red precipitate was collected by filtration, washed with water (3 × 15 mL) and acetone (2 × 5 mL) and dried. Recrystallization from THF/EtOAc yielded **1d** (302 mg, 93%) as dark red crystals (m.p. >250 °C, HPLC 99 %). Physical and chromatographic properties were identical with those of our authentic sample and literature data [59].

#### 4.1.6. General Procedure for the Synthesis of Active Esters **1a-ester**, **1b-ester** and **1c-ester**

Compounds **1a**, **1b** or **1c** (0.15 mmol) were dissolved in the mixture of dichloromethane (10 mL) and acetonitrile (2 mL). The mixture was cooled in an ice bath and *N*-hydroxysuccinimide (20 mg, 0.167 mmol) and *N*-(3-dimethylaminopropyl)-*N′*-ethylcarbodiimide hydrochloride (EDC) (31 mg, 0.16 mmol) were added. The reaction mixture was allowed to warm up to room temperature and stirred for 24 h. The solvent was evaporated and the product was purified by flash column chromatography (hexane/EtOAc 7:3 for **1a-ester**; 6:4→1:1 for **1b-ester**, and 7:3 for **1c-ester**, respectively).

**1a-ester**: yield 55.4 mg (76%) of dark red powder; *R*_f_ = 0.33 (hexane/EtOAc 1:1); the product was used in the next step without NMR characterization; HRMS (MALDI): *m/z* calcd for C_29_H_33_N_1_O_6_+Na^+^: 514.2200 [*M*+Na]^+^; found: 514.2207.

**1b-ester**: yield 37.5 mg (57%) of orange powder; *R*_f_ = 0.40 (hexane/EtOAc 1:1); the product was used in the next step without NMR characterization; HRMS (MALDI): *m/z* calcd for C_25_H_29_N_1_O_6_+Na^+^: 462.1887 [*M*+Na]^+^; found: 462.1890.

**1c-ester**: yield 56 mg (71%) of red powder; *R*_f_ = 0.42 (hexane/EtOAc 1:1); ^1^H NMR (400 MHz, CDCl_3_) *δ* (ppm) 7.53 (dd, 1H, *J* = 11.4, 1.6 Hz, C*H*), 6.82–6.09 (m, 11H, 11C*H*), 2.86 (d, 4H, 2C*H*_2_), 2.08 (d, 3H, *J* = 1.2 Hz, C*H*_3_), 2.06–1.94 (m, 11H, 3C*H*_3_ and 1C*H*_2_), 1.72 (s, 3H, C*H*_3_), 1.66–1.57 (m, 2H, C*H*_2_), 1.50–1.44 (m, 2H, C*H*_2_), 1.03 (s, 6H, 2C*H*_3_); ^13^C NMR (100 MHz, CDCl_3_) *δ* (ppm) 169.6, 163.5 (3C, *C* = O), 147.2, 144.1, 138.0, 137.8, 137.0, 133.4, 132.1, 130.8, 129.3, 127.3, 126.3, 122.3 (12C, *C*H), 138.8, 135.2, 129.7, 120.1 (6C, Cq), 39.8 (1C, *C*H_2_), 34.4 (1C, Cq), 33.2 (1C, *C*H_2_), 29.1 (2C, *C*H_3_), 25.8 (2C, *C*H_2_), 21.9 (1C, *C*H_3_), 19.4 (1C, *C*H_2_), 13.1, 13.0, 12.8 (3C, *C*H_3_). HRMS (MALDI): *m/z* calcd for C_34_H_43_N_1_O_4_+Na^+^: 552.3084 [*M*+Na]^+^; found: 552.3159.

#### 4.1.7. General Procedure for the Synthesis of Apocarotenoid–Glycopeptide Conjugates **5a**, **5b**, **5c**, **6** and **7**

Glycopeptide derivative (**2**, **3** or **4**) (0.05 mmol) was dissolved in dimethylformamide (2 mL) and triethylamine (7 µL, 0.05 mmol) and apocarotenoid active ester (**1a-ester**, **1b-ester** or **1c-ester**) (0.075 mmol) was added. The reaction mixture was stirred overnight; then, the solvent was evaporated and the product was purified by flash column chromatography (toluene/MeOH 7:3→1:1 for **5a**, **5b**, **5c** and **7**; acetonitrile/H_2_O 95:5→9:1 for **6**, respectively).

Compound **5a**: yield 65.5 mg (74%, HPLC purity 97.4%), dark red powder; *R*_f_ = 0.28 (toluene/MeOH 1:1); NMR data of **5a** are shown in Table 4.

HRMS (ESI): *m/z* calcd for C_91_H_85_Cl_2_N_8_O_26_Na+Na^+^: 1821.4742 [*M*-H+2Na]^+^; found: 1821.4742.

Compound **5b**: yield 43 mg (51%, HPLC purity 96.1%), orange powder; *R*_f_ = 0.27 (toluene/MeOH 1:1); NMR data of **5b** are shown in Table 5. HRMS (ESI): *m/z* calcd for C_87_H_82_Cl_2_N_8_O_26_Na+Na^+^: 1769.4429 [*M*-H+2Na]^+^; found: 1769.4428;

Compound **5c**: yield 47.5 mg (53%, HPLC purity 96.6%), red powder; *R*_f_ = 0.29 (toluene/MeOH 1:1); NMR data of **5c** are shown in Table 6. HRMS (ESI): *m/z* calcd for C_96_H_95_Cl_2_N_8_O_24_+Na^+^: 1859.5626 [*M*-H+2Na]^+^; found: 1859.5627.

**Figure 5:**
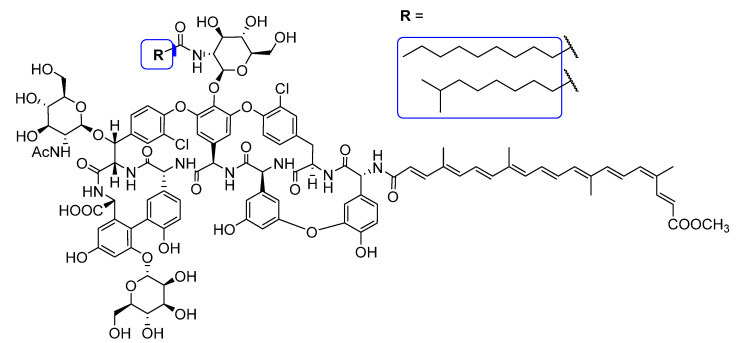
Structure of the two major components of conjugate **6**.

Compound **6**: yield 96 mg (85%), red powder; *R*_f_ = 0.32 (acetonitrile/H_2_O 85:15); Structures of the two major components of **6** are shown in Figure 5. RP-HPLC-ESI MS for the main peak (63.2%): RT = 26.97 min, *m/z* calcd for C_113_H_125_Cl_2_N_9_O_36_+2H^+^: 1127.887 [*M*+2H]^2+^; found: 1127.887.

Compound **7**: yield 14 mg (18%, HPLC purity 95.7%) red powder; *R*_f_ = 0.51 (toluene/MeOH 1:1); NMR data of **7** are shown in Table 7. HRMS (MALDI): *m/z* calcd for C_85_H_79_N_7_O_22_+Na^+^: 1572.5176 [*M*+Na]^+^; found: 1572.5670.

#### 4.1.8. Synthesis of **9**

*tert*-Butyl l-phenylalaninate (52 mg, 0.2 mmol) was dissolved in abs. dimethylformamide (2 mL) and 4-methylmorpholine (18 μL, 0.22 mmol) was added and the mixture was stirred for 30 min. Under argon atmosphere, **1a-ester** (49 mg, 0.1 mmol) was added and the reaction mixture was stirred overnight. The solvent was evaporated and the product was purified by flash column chromatography (hexane/EtOAc 8:2→7:3) to yield **9** (40 mg, 67%, HPLC purity 95.9%) as a dark red powder. *R*_f_ = 0.56 (hexane/EtOAc 7:3); ^1^H NMR (400 MHz, CDCl_3_) *δ* (ppm) 7.96 (d, 1H, *J* = 15.5 Hz, C*H*), 7.35–7.20 (m, 4H, C*H*), 7.20–7.14 (m, 2H, C*H*), 6.91–6.80 (m, 1H, C*H*), 6.71–6.64 (m, 2H, C*H*), 6.64–6.57 (m, 1H, C*H*), 6.56–6.28 (m, 6H, C*H*), 6.03 (d, 1H, *J* = 7.7 Hz, N*H*), 5.91 (d, 1H, *J* = 15.5 Hz, C*H*), 5.84 (d, 1H, *J* = 15.2 Hz, C*H*), 4.92–4.84 (m, 1H, C*H*-CH_2_-Ph), 3.79, (s, 3H, OC*H*_3_), 3.15 (d, 2H, *J* = 5.8 Hz, benzyl C*H*_2_), 2.00 (s, 3H, C*H*_3_), 1.98 (s, 3H, C*H*_3_), 1.96 (s, 3H, C*H*_3_), 1.92 (s, 3H, C*H*_3_), 1.42 (s, 9H, *t*-Bu C*H*_3_); ^13^C NMR (100 MHz, CDCl_3_) *δ* (ppm) 171.0, 168.1, 165.9 (3C, *C* = O), 145.9, 141.2, 140.6, 138.7, 138.1 (6C, *C*H), 136.9, 136.8, 136.4 (3C, *C*q), 134.6, 134.5 (2C, *C*H), 133.5, 131.6 (2C, *C*H), 131.1, 131.0, 129.8, 128.5, 127.0, 124.6, 123.3, 118.6, 117.6 (11C, *C*H), 82.6 (1C, *C*q), 53.8 (1C, *C*H-CH_2_-Ph), 51.8 (1C, O*C*H_3_), 38.3 (1C, benzyl *C*H_2_), 28.1 (3C, *t*-Bu *C*H_3_), 20.4 (1C, *C*H_3_), 13.2, 13.0, 12.9 (3C, *C*H_3_). HRMS (ESI): *m/z* calcd for C_38_H_47_NO_5_+H^+^: 598.353 [*M*+H]^+^; found: 598.351.

### 4.2. Biological Studies

#### 4.2.1. Antiviral Activity Screening Using Viral RNA Reduction Assay

The inhibitory effect of the different compounds against a Hungarian SARS-CoV-2 isolate (GISAID accession ID: EPI_ISL_483637) was measured in Vero E6 cells. The compounds were dissolved in DMSO for obtaining stock solutions. For the antiviral screen, dilution was conducted in cell culture media that consisted of DMEM (Lonza), 1% penicillin–streptomycin (Lonza) and 2% heat-inactivated foetal bovine serum (Gibco). Infection (MOI 0.01) and treatment were performed at the same time for 30 min at 37 °C in 5% CO_2_ atmosphere. After, the infection the media was replaced and the cells were incubated for 48 h in the presence of the different compounds. RNA was extracted from the supernatant using Monarch^®^ Total RNA Miniprep Kit (New England Biolabs Inc., Ipswich, MA, USA). Viral copy number was determined with droplet-digital PCR (Bio-Rad Laboratories Inc., Hercules, CA, USA) Primers and probe were specific for the SARS-CoV-2 RdRp-gene (forward: GTGARATGGTCATGTGTGGCGG; reverse: CARATGTTAAASACACTATTAGCATA, probe FAM-CAGGTGGAACCTCATCAGGAGATGC-BBQ). Copy numbers were normalized to the mean for the untreated, infected wells, *n* = 3 biological replicates. EC_50_ values were determined with GraphPad Prism 8 software using a four-parameter logistic nonlinear regression model. Cell viability was determined using CellTiter-Glo^®^ Luminescent Cell Viability Assay (Promega Corp., Madison, WI, USA). The same concentrations were applied as in the antiviral screen. After 48 h, the viability was measured according to the manufacturer’s instructions.

#### 4.2.2. Antiviral Activity Determination Using CPE-Based Assay

In the cytopathic effect (CPE)-based assay, the anti-SARS-CoV-2 activity was measured as the extent of the test compounds inhibited virus-induced CPE in Vero E6 cells. Briefly, two-fold serial dilutions of compounds were prepared from 100 to 0.78 µM and added in triplicate in a 384-well plate with 5000 Vero E6 cells seeded four hours before in DMEM medium with 2% FBS, 100 U of penicillin/mL and 100 µg of streptomycin/mL (all Merck). After 1 h incubation, SARS-CoV-2 (strain hCoV-19/Czech Republic/NRL_6632_2/2020) was added at multiplicity of infection 0.04 IU/mL. Following three days incubation at 37 °C in 5% CO_2,_ the cell viability was determined by addition of XTT solution (Sigma-Aldrich) for 4 h and the absorbance was measured using EnVision plate reader (PerkinElmer, Waltham, MA, USA). Drug concentrations required to reduce viral cytopathic effect by 50% (EC_50_) were calculated using nonlinear regression from plots of percentage cell viability versus log10 drug concentration using GraphPad Prism software.

#### 4.2.3. Antiviral Activity Determination Using Immunofluorescence Assay (IFA)

The anti-SARS-CoV-2 activity was measured by determining the extent to which the test compounds inhibited virus replication in Vero E6 cells represented as the reduction in SARS-CoV-2 nucleoprotein detected by IFA. Briefly, two-fold serial dilutions of compounds from 100 µM were added in triplicate in a 96-well plate with 15,000 Vero plated day before in the same medium as above. After one hour incubation, SARS-CoV-2 (strain hCoV-19/Czech Republic/NRL_6632_2/2020) was added at multiplicity of infection 0.04 IU/cell. After three days of incubation at 37 °C in 5% CO_2_, cells were fixed with 4% paraformaldehyde, permeabilized with 0.2% Triton X100 (both Merck), washed, incubated with anti-SARS-CoV-2 antibody (mouse monoclonal nucleoprotein IgG, ProSci) for 2 h at room temperature, followed by 1 h incubation with Cy3-conjugated donkey anti-mouse IgG (Jackson ImmunoResearch Europe, Ely, UK) and documented using fluorescence microscope with camera (Olympus). The compound concentrations required to reduce fluorescence by 50% (EC_50_) were calculated from graphs of percentage of fluorescent cells versus log10 drug concentration using nonlinear regression analysis with GraphPad Prism software.

#### 4.2.4. Determination of Compound Cytotoxicity in Vero E6 Cells

Cytotoxicity was evaluated by incubating two-fold serial dilutions of each compound from 100 µM concentration with Vero E6 cells in 384-well plate. Following three days incubation at 37 °C in 5% CO_2_, the cell viability was determined by addition of XTT solution and the compound concentrations resulting in 50% reduction in absorbance (CC_50_), corresponding to 50% reduction in viability, were calculated as above in the antiviral activity determination using CPE-based assay.

#### 4.2.5. Cathepsin Inhibition Assays

##### Enzyme Kinetics

Human recombinant cathepsins B and L were expressed in *Escherichia coli* [60,61]. The assay buffers 60 mM acetate buffer, pH 5.0 and 100 mM acetate buffer, pH 5.5 were used for determination of cathepsin B exopeptidase and cathepsin L activities, respectively. Each assay buffer contained 0.1% PEG 8000 (Sigma-Aldrich, St. Louis, MO, USA), 5 mM cysteine and 1.5 mM EDTA. Enzymes were activated in the assay buffer for 5 min at 37 °C prior to the assay.

##### Determination of Relative Inhibition

Substrates Abz-Gly-Ile-Val-Arg-Ala-Lys(Dnp)-OH (Bachem, Basel, Switzerland) and Z-Phe-Arg-AMC (Bachem) were used to determine the effect of inhibitors on cathepsin B exopeptidase and cathepsin L activity, respectively. To initiate the reaction, 90 μL of activated enzyme in the assay buffer was added to the wells of a black microplate containing 5 μL of substrate (final concentration 5 and 1 μM for cathepsin B exopeptidase activity, and 2 μM for cathepsin L) and 5 μL of inhibitor at concentration of 50 μM. Formation of the fluorescent degradation products during reaction was continuously monitored at 460 nm ± 10 nm with excitation at 380 nm ± 20 nm for Z-Phe-Arg-AMC and at 420 nm ± 10 nm with excitation at 320 nm ± 20 nm for Abz-Gly-Ile-Val-Arg-Ala-Lys(Dnp)-OH at 37 °C on a Tecan Infinite M1000 (Mannedorf, Switzerland) spectrofluorimeter. All assay mixtures contained 5% (*v/v*) DMSO. To all assay mixtures, 0.01% Triton X-100 was also added, to prevent false-positive inhibition due to the formation of compound aggregates [62]. All measurements were performed in triplicates and repeated twice. The relative inhibition was calculated using the equation: Relative inhibition (%) = 100(1 − v_i_/v_o_); where v_i_ and v_o_ designate the reaction velocities in the presence and absence of inhibitor, respectively. IC_50_ values were determined from nine data points at different inhibitor concentrations.

##### CLPro Inhibition Assay, Cloning and Expression

3CLPro coding sequence (GenBank: MN908947.3) was optimized according to *E. coli* codon preference and synthetized by Thermo Fisher Scientific, Inc. (Waltham, MA, USA). We inserted the gene into pPAL7 (Bio-Rad Laboratories, Inc., Hercules, CA, USA) expression vector using BamHI and HindIII enzymes. For the recombinant protein expression, we used Rosetta2 (Novagen) cells and o/n induction with 1 mM IPTG at 20 °C.

##### Purification of Recombinant 3CLPro Protein

Thawed cell stocks were suspended in 0.1 M NaH_2_PO_4_ buffer (pH = 7.4), then homogenized on ice using ultrasonic homogenizer. The homogenized sample was centrifuged at 13,000× *g* for 30 min. The supernatant was filtered through a 0.45 µm pore size cellulose acetate membrane filter and loaded on Mini Profinity eXact cartridges (Bio-Rad Laboratories, Inc., Hercules, CA, USA) at room temperature. After washing, 2 CV 100% elution buffer (0.1 M NaH_2_PO_4_, 0.1 M NaF, pH = 7.4) was loaded to start the enzymatic reaction in the column, for 30 min. The elution was performed with 100% elution buffer. The eluate was saturated with 50% (NH_4_)_2_SO_4_ at 4 °C, and was kept at 4 °C overnight. The next day, the sample was centrifuged at 13,000× *g*, the supernatant was carefully decanted, and the precipitate was redissolved in 3CLPro reaction buffer (0.1 M NaH_2_PO_4_, 1 mM EDTA, 100 mM NaCl, pH = 7.5) and stored at 4 °C.

##### Enzyme Activity Assays

For the fluorescence measurements, a generic 3CLPro FRET peptide substrate was used (Hilyte^TM^ Fluor—488—ESATLQSGLRKAK—(QXL^®^—520)—NH2, Anaspec, Inc., (Fremont, CA, USA) Cat. no. 510/791-9560) [63]. The conditions of the measurements were as follows: either 600 nM or no 3CLPro, 250 nM substrate, 5% DMSO and the requisite concentration of inhibitors in 3CLPro reaction buffer at a final volume of 50 µL/well. The covalent 3CLPro inhibitor 5-Chloropyridin-3-yl-benzo-(b)-thiophene-2-carboxylate (Maybridge, Ltd., Altrincham, UK, Cat. no. BTB07408SC) was used in the assays in 10 µM final concentration as a control [64]. Fluorescence was measured on a black 384-well plate (Thermo Fisher Scientific Inc., Waltham, MA, USA, Cat. no. 95040020) with a fluorescence microplate reader (Victor^2^ 1420 multilabel counter, PerkinElmer Inc., Waltham, MA, USA) at 485/520 nm for excitation and emission wavelength, respectively, in duplicates. The candidates were first screened in 100 µM final concentrations. Those showing more than 50% inhibition were tested at a two-fold dilution series during a dose–response measurement, consisting of 6–8 data points. IC_50_ values were determined using a logistic curve fit (Origin 8, Northampton, MA, USA).

#### 4.2.6. Antibacterial Evaluations

Antibacterial activity of the compounds was assessed with broth microdilution method following the Clinical and Laboratory Standards Institute (CLSI) guidelines (CLSI. Methods for Dilution Antimicrobial Susceptibilities Tests for Bacteria that Grow Aerobically; Approved Standard, 11th edition CLSI document M07-A10. Wayne, PA: Clinical Laboratory and Standards Institute; 2018 [65]). Stock solutions of the substances were prepared in H_2_O and DMSO (1:1). Bacterial cells (0.5 McFarland in saline) were inoculated into ca-MH broth (BioLab Inc., Budapest, Hungary) containing the active compound in a twofold serial dilution series ranging between 256 and 0.5 µg mL^−1^. The minimum inhibitory concentrations (MICs) of all compounds were determined after 24 h of incubation at 37 °C. The MIC was determined as the lowest concentration of the drug that resulted in no visible bacterial growth.

### 4.3. Low-Mode Docking

To predict the binding modes of bixin **1a** and the teicoplanin pseudoaglycone conjugate **5a** in the binding site of cathepsins B (PDB: 6AY2) [66] and L (PDB: 3H8B) [67] and 3CLPro (PDB: 6LU7) [49], we used low-mode docking [47]. Low-mode docking was performed with the LMOD conformational searching algorithm [48], which sampled the conformational space along low-frequency vibrational modes of the part of the system that was defined as flexible (usually the ligand and proximal residues/loops), and, additionally, explored the rotational and translational degrees of freedom of the ligand. As such, it was a computationally efficient implementation of flexible docking, and it presented an ideal alternative over conventional docking for large ligands with a complex conformational space, such as the compounds considered here. For the calculations presented here, we performed 10 LMOD iterations after defining the ligands and the flexible loops in the vicinity of the binding sites as flexible, and kept low-energy conformations within an energy window of 50 kcal/mol as the output of the calculations. The full protocol was described in the Supplementary Information. Marvin 21.4 (2021) was used for drawing and preparing chemical structures (ChemAxon, http://www.chemaxon.com, last accessed: 28 June 2021), Pymol was used for preparing images (Schrödinger LLC and Warren L. DeLano, 2020, http://www.pymol.org/pymol, last accessed: 30 June 2021) and the Amber biomolecular simulation package was used to run the LMOD calculations [68].

## 5. Conclusions

The aim of this work was to study the effect of lipophilic carotenoid side chains on the anti-SARS-CoV-2 activity of the glycopeptide antibiotic teicoplanin. Indeed, several derivatives were obtained, which showed remarkable antiviral activity and were completely devoid of cytotoxicity. However, the anti-SARS-CoV-2 activity of bixin seemed to be one of the most promising results. Bixin and annatto are widely used, cheap, non-toxic, natural food colorants [69,70], approved by the European Food Safety Authority. The acceptable daily intake of bixin is 6 mg/kg body weight [70]. Conjugates of teicoplanin pseudoaglycone with apocarotenoids such as bixin and apocarotenoic acid offer a combination of antiviral and antibacterial effects.

It is worth noting that the antiviral activity of the antibiotic teicoplanin against wild-type SARS-CoV-2 was first demonstrated in our present work. Based on our results, the anti-SARS-CoV2 effect of teicoplanin could not be satisfactorily explained by any of the mechanisms of action hypothesized in the literature, namely, the inhibition of either cathepsin L [21,22] or the viral main protease [46], as teicoplanin exerted only low inhibitory activity against these enzymes. Therefore, the full mechanism of action of teicoplanin against SARS-CoV-2 remains to be elucidated. For the carotenoid conjugates, the enzyme inhibitory activities seemed to contribute to the antiviral effect; however, their generally weak activity suggested that the contribution of other mechanisms could not be ruled out.

## Figures and Tables

**Table 1:** Antiviral effects of the glycopeptide conjugates and carotenoids according to the three assays.

Compound	SARS-CoV-2 RNA Reduction EC_50_ (µM)	SARS-CoV-2 CPE, EC_50_ (µM)	SARS-CoV-2 IFA, EC_50_ (µM)	CC_50_ (µM)	Therapeutic Index *** CC_50_/EC_50_
**1a**	5.9 ± 1.7	14 ± 3.5	28 ± 8.8	>100	>17
**1b**	not active at 50 µM	n.d.	n.d.	n.d.	n.d.
**1c**	15 ± 2	14 ± 2.2	31 ± 7.9 *	>100 **	>6.7
**1d** (norbixin)	not active at 50 µM	n.d.	n.d.	>100	n.d.
Annatto (E-160b)	9.2 ± 0.1	n.d.	n.d.	>100	>10.9
**2**	not active at 50 µM	n.d.	n.d.	n.d.	n.d.
**3**	5.6 ± 1.3	16 ± 1.7	18 ± 5.4	>100	>17.9
**4**	not active at 50 µM	n.d.	n.d.	n.d.	n.d.
**5a**	5.9 ± 1.3	91 ± 88	74 ± 20	>100	>17
**5b**	5.2 ± 0.2	19 ± 1.8	8.8 ± 1.4	>100	>19.2
**5c**	4.4 ± 0.4	56 ± 10	24 ± 5.3	>100	>22.7
**6**	1.8 ± 0.2	56 ± 6.2	51 ± 9.8	>100	>55.6
**7**	6.7 ± 0.4	n.d.	n.d.	n.d.	n.d.
**9**	n.d.	31 ± 4.7	n.d.	>100	n.d.

n.d.—not determined; * measurement from 50 µM; ** cytotoxic at 100 µM; *** calculated from RNA reduction EC_50_ values.

**Table 2:** Cathepsins B and L, and 3CLPro inhibitory effects of the glycopeptide conjugates and carotenoids.

Compound	Exopeptidase Cathepsin B IC_50_ (µM)	Cathepsin L IC_50_ (µM)	3CLPro IC_50_ (µM)
**1a** (bixin)	14.56 ± 1.71	41.92 ± 1.12	56% at 100 μM
**1b**	n.d.	n.d.	24% at 100 μM
**1c**	21.31 ± 1.20	76.04 ± 1.74	73.00 ± 7.10
**1d** (norbixin)	n.d.	n.d.	36.11 ± 11.71
**2**	7.80% at 50 μM	0.04% at 50 μM	34% at 200 μM
**3**	0.45% at 50 μM	5.07% at 50 μM	13% at 200 μM
**5a**	47.95 ± 1.07	42.39 ± 1.86	13.86 ± 1.73
**5b**	99.54 ± 1.16	103.0 ± 1.12	50% at 200 μM
**5c**	56.53 ± 1.06	47.05 ± 1.05	34.52 ± 12.49
**6**	64.42 ± 1.05	56.51 ± 1.08	53% at 200 μM
**9**	23.78% at 50 μM	22.08% at 50 μM	42% at 100 μM

**Table 3:** Antibacterial effect.

	1a	Teico (3)	5a	5b	5c	6	7	9
	MIC ^[g]^ (μg/mL)							
Bacillus subtilis ATCC ^[a]^ 6633	512	0.5	32	16	16	4	32	512
Staphylococcus aureus MSSA ^[b]^ ATCC 29213	512	0.5	16	8	8	16	64	512
Staphylococcus aureus MRSA ^[c]^ ATCC 33591	512	0.5	0.5	2	1	16	64	512
Staphylococcus epidermidis ATCC 35984 biofilm	512	2	0.5	0.5	0.5	32	64	512
Staphylococcus epidermidis mecA ^[d]^	512	16	16	2	0.5	256	64	512
Enterococcus faecalis ATCC 29212	512	2	16	4	4	4	32	512
Enterococcus faecalis 15376 VanA ^[e]^	512	256	16	8	8	4	256	512
Enterococcus faecalis ATCC 51299 VanB ^[f]^	512	4	16	8	8	4	64	512

^[a]^ American Type Culture Collection. ^[b]^ Methicillin-sensitive *Staphylococcus aureus*. ^[c]^ Methicillin-resistant *Staphylococcus aureus*. ^[d]^ mecA gene expression in Staphylococcus. ^[e]^ vanA gene positive. ^[f]^ vanB gene positive. ^[g]^ Minimum inhibitory concentration.

**Table 4:** NMR data of **5a**.

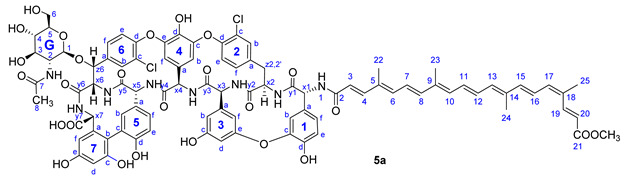		
Annotation	^1^H Shift (ppm)	^13^C Shift (ppm)
19	7.899	140.42
1b	6.788	119.07
1e	6.993	119.09
1f	7.039	125.61
20	5.959	118.11
21 (OMe)	3.707	51.84
22,23	1.976	13.07
24	1.897	13.07
25	1.940	20.33
2b	7.226	131.46
2e	7.269	125.33
2f	7.706	130.68
3	6.269	120.85
3b	6.360	110.56
3d	6.375	105.48
3f	6.323	102.23
4	7.246	144.29
4b	5.563	108.03
4f	5.113	104.97
5b	7.120	136.49
5e	6.667	116.99
5f	6.673	125.82
6b	7.872	128.98
6e	7.265	123.66
6f	7.256	128.61
7d	6.420	103.70
7f	6.495	108.28
DMSO	2.505	40.24
G1	4.396	99.67
G2	3.510	56.32
G3	3.218	70.59
G4	3.412	73.82
G5	3.104	77.26
G6a	3.641	60.84
G6b	3.582	60.85
X1	4.340	59.57
X2	4.947	55.17
X3	5.360	58.54
X4	5.629	55.33
X5	4.359	54.13
X6	4.160	61.39
X7	5.769	56.32
z2a	3.327	37.19
z2b	2.842	37.19
z6	5.393	76.54

**Table 5:** NMR data of **5b**.

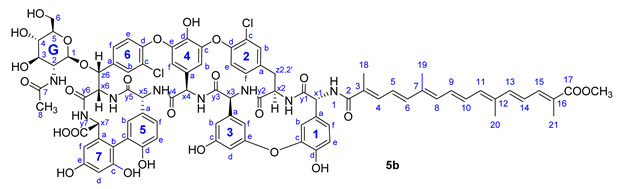		
Annotation	^1^H Shift (ppm)	^13^C Shift (ppm)
1b	6.789	119.70
1e	7.043	118.97
1f	7.043	125.86
2b	7.214	131.42
2e	7.265	125.40
2f	7.670	130.69
3b	6.353	110.47
3d	6.390	105.40
3f	6.366	102.41
3xMe	1.963	13.06
4?	7.235	139.13
4b	5.562	108.09
4f	5.108	105.05
5b	7.125	136.50
5e	6.675	116.99
5f	6.674	125.87
6b	7.863	128.99
6e	7.247	123.67
6f	7.251	128.51
7d	6.386	103.42
7f	6.440	108.05
DMSO	2.510	40.28
G1	4.375	100.12
G2	3.525	56.13
G3	3.215	70.45
G4	3.408	73.78
G5	3.088	77.15
G6a	3.644	60.67
G6b	3.588	60.66
Me	1.967	13.52
OMe	3.694	52.12
X1	4.330	59.23
X2	4.953	55.13
X3	5.334	58.51
X4	5.621	55.33
X5	4.363	54.11
X6	4.173	61.35
X7	5.710	56.90
z2a	3.310	37.30
z2b	2.828	37.31
z6	5.333	77.00

**Table 6:** NMR data of **5c**.

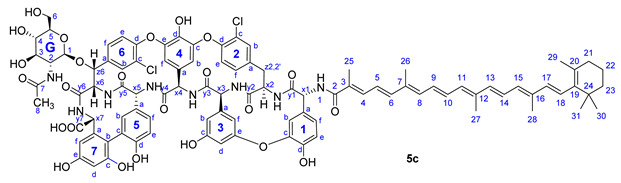		
Annotation	^1^H Shift (ppm)	^13^C Shift (ppm)
1b	6.792	119.57
1e	6.976	119.05
1f	7.060	125.92
21	2.018	33.10
22	1.589	19.23
23	1.455	39.75
25, 26, 27	1.959	13.01
28	1.974	13.51
29	1.701	21.98
2b	7.231	131.47
2e	7.285	125.36
2f	7.678	130.63
30, 31	1.029	29.30
3b	6.365	110.51
3d	6.368	105.39
3f	6.418	103.39
4b	5.568	107.94
4f	5.117	104.94
5b	7.122	136.49
5e	6.642	116.93
5f	6.682	125.95
6b	7.879	128.97
6e	7.257	123.66
6f	7.259	128.64
7d	6.300	102.09
7f	6.511	108.42
DMSO	2.513	40.31
G1	4.423	99.26
G2	3.515	56.47
G3	3.241	70.58
G4	3.412	73.91
G5	3.114	77.34
G6	3.603	60.89
X1	4.337	59.69
X2	4.976	55.12
X3	5.357	58.50
X4	5.631	55.38
X5	4.364	54.13
X6	4.152	61.43
X7	5.731	56.87
z2a	2.845	37.32
z2b	3.333	37.31
z6	5.442	76.09

**Table 7:** NMR data of **7**.

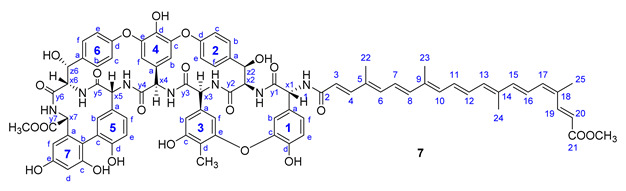		
Annotation	^1^H Shift (ppm)	^13^C Shift (ppm)
1b	6.716	118.01
1e	6.956	118.87
1f	7.013	124.98
20	5.961	118.15
3	6.293	121.15
3b	6.416	109.95
3f	6.470	104.05
4b	5.655	107.36
4f	5.308	106.17
5b	7.113	136.00
5e	6.623	117.25
5f	6.678	126.12
6b	7.843	128.64
6c	7.191	123.30
7d	6.385	103.29
7f	6.066	105.68
Bix21 (OMe)	3.710	51.84
Me (3d)	1.971	8.79
OMe-7	3.699	55.41
x1	4.509	57.10
x2	5.946	55.65
x3	5.261	58.51
x4	5.579	55.11
x5	4.418	53.99
x6	4.182	62.49
x7	5.075	60.89
z2	5.145	71.72
z6	5.129	72.31

## Data Availability

Data is contained in this article.

## Supplementary Materials

The following are available online at https://www.mdpi.com/article/10.3390/ph14111111/s1, HPLC chromatograms, EC_50_ graphs of antiviral assays, IC_50_ graphs of enzyme inhibitory assays and ^1^H and ^13^C NMR spectra of compounds.
