# Supplementary material for: Natural Apocarotenoids and Their Synthetic Glycopeptide Conjugates Inhibit SARS-CoV-2 Replication
Source: Pharmaceuticals (Basel). 2021 Oct 30;14(11):1111. doi: 10.3390/ph14111111 (PMC8619593; doi:10.3390/ph14111111)

Supplementary information

Natural apocarotenoids and their synthetic glycopeptide conjugates inhibit SARS-CoV-2 replication

Ilona Bereczki, Henrietta Papp, Anett Kuczmog, Mónika Madai, Veronika Nagy, Attila Agócs, Gyula Batta, Márton Milánkovits, Eszter Ostorházi, Ana Mitrović, Janko Kos, Áron Zsigmond, István Hajdú, Zsolt Lőrincz, Dávid Bajusz, Miklós György Keserű,* Jan Hodek, Jan Weber,* Ferenc Jakab,* Pál Herczegh,* Anikó Borbás*

Table of contents

Experimental S3

General information S3

HPLC Chromatogram of the crude annatto powder S4

Isolation of bixin (1a) S5

Synthesis of crocetin monomethyl ester (1b) [5] S6

Synthesis of β-apo-8'-carotenoic acid (1c) S7

Synthesis of norbixin (1d) S8

General procedure for the synthesis of active esters 1a-ester, 1b-ester and 1c-ester S9

General procedure for the synthesis of apocarotenoid-glycopeptide conjugates 5a, 5b, 5c, 6, 7 .... S11

Synthesis of 9 .... S21

Antiviral activity determination based on viral RNA reduction assay S23

Antiviral activity determination using CPE-based assay S24

Antiviral activity determination using immunofluorescence assay (IFA) S25

Cathepsin inhibition assays S30

3CLPro inhibition assay S32

Computational modeling S34

References S35

NMR spectra S38

NMR spectra of crocetin monomethyl ester (1b) S38

NMR spectra of β-apo-8'-carotenoic acid (1c) S39

NMR spectra of 1c-ester S41

NMR spectra of 9 .... S42

NMR spectra of glycopeptide derivatives 5a, 5b, 5c and 7 (125 MHz and 500 MHz in DMSO) S44

Experimental

General information

tert-Butyl L-phenylalaninate **8** was synthesized according to the literature [1]. For the synthetic glycopeptide derivatives (**5a**, **5b**, **5c**, **7**) initial signal assignments were aided using earlier works on glycopeptide aglycon NMR characterisations [2,3], unlabelled peaks in the 6-8/110-145 ppm $^{1}H/^{13}C$ regions belong to carotenoid sidechain atoms. Typical 90° pulses were 10 and 16 µs for the ^{1}H and ^{13}C channels, and relaxation delays allowed were generally 2s. ^{1}H-^{13}C heteronuclear Single Quantum Correlation (HSQC) spectra were recorded using «hsqcetgpsi2» pulse program and 4-8 scans for each of the 512 increments in indirect dimension. The 2D spectra were processed with Topspin 3.1 software using Gaussian window function (Lb= -5, GB= 0.05) in F2 and cosine-square (QSINE, SSB = 2) in F1 dimension. To support the $^{1}H/^{13}C$ assignments heteronuclear multiple bond correlation experiments, HMBC (pulse program: "hmbcgplpndqf", 70 ms evolution time), HSQC-TOCSY (pulse program: "hsqcdietgpsi", 70 ms mixing time), and homonuclear correlated spectroscopy (COSY) (pulse program: "cosygpqf") were also run. The digital resolution of the processed spectra was typically 2–3 Hz. ^{13}C J-modulated spin-echo and ^{1}H-^{13}C HSQC spectra of glycopeptide derivatives (**5a**, **5b**, **5c**, and **7**) are shown at the end of the supplementary material, equipped with characteristic ^{1}H-^{13}C signal assignments.

HPLC Method for **1a**, **1b**, **1d** and **annatto powder**:

Eluents: (A) MeOH; (B) $CH_3COOH:H_2O$ = 1:99 v/v%. The chromatography was performed in isocratic conditions with 90% A and 10% B eluents, 1.00 mL/min flow rate at 24 °C. Measurements were performed at 450 nm.

HPLC Chromatogram of the crude annatto powder

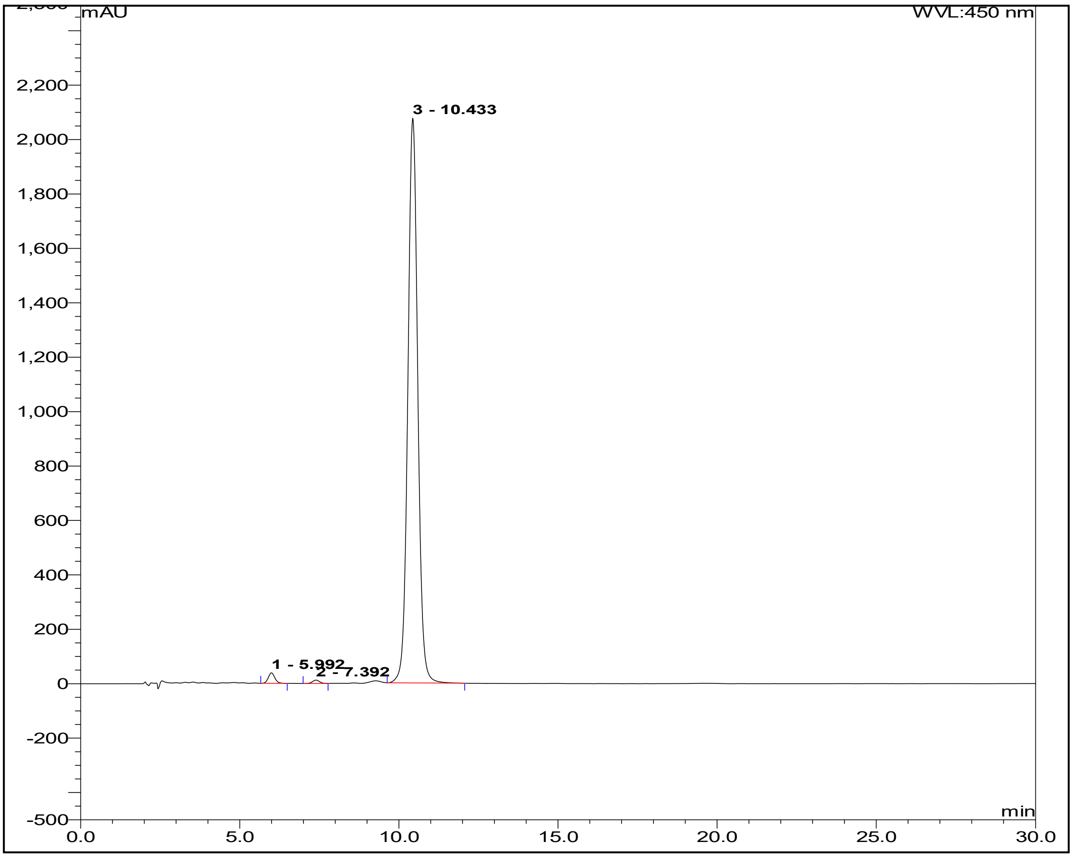

No.	Ret.Time min	Peak Name	Height mAU	Area mAU*min	Rel.Area %	Amount	Type
1	5,99	n.a.	38,322	9,123	1,20	n.a.	BMB
2	7,39	n.a.	12,317	3,130	0,41	n.a.	BMB
3	10,43	n.a.	2075,887	748,970	98,39	n.a.	BMB
**Total:**			2126,526	761,224	100,00	0,000	

Figure S1. HPLC Chromatogram of the crude annatto powder (INEXA bixin powder) at 450 nm. Bixin content according to this HPLC is 98%. Bixin content was determined also by using calibration curve with pure bixin, it gave 80 % bixin content, this can be regarded as real bixin content.

Isolation of bixin (1a)

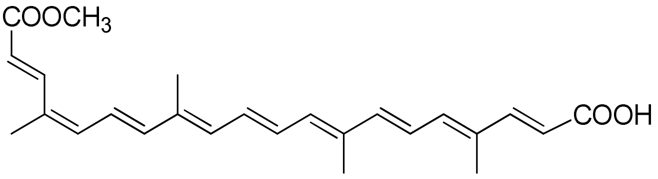

1a

500 mg of *Bixa orellana* seed extract (INEXA C.A., Ecuador) was flash chromatographed on a 100 g silica gel column in dichlomethane/methanol 10:1. The fractions containing the main reddish violet pigment were collected and evaporated. The crude bixin was crystallized from dichloromethane/hexane to yield 170 mg of bixin **1a** (m.p. 198 °C, HPLC purity 98%). Physical and chromatographic properties were identical with those of our authentic sample and literature data [4].

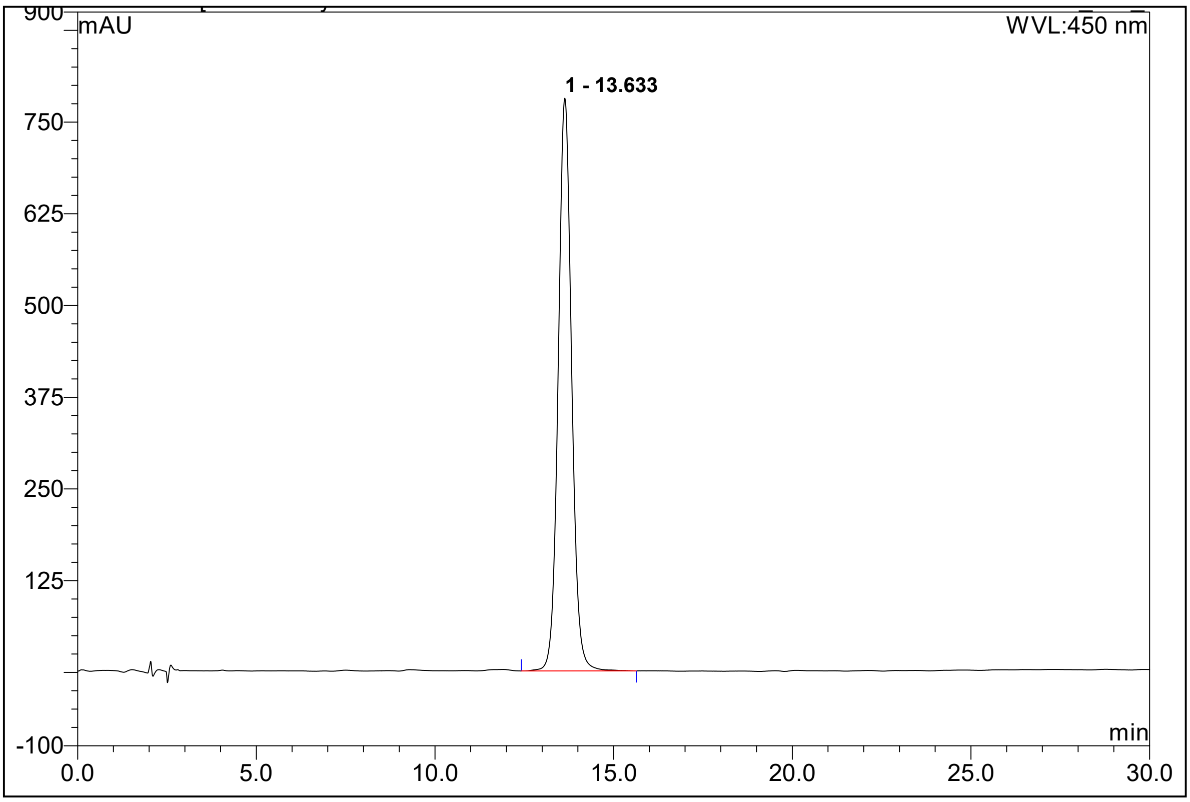

Figure S2. HPLC Chromatogram of bixin **1a**

Synthesis of crocetin monomethyl ester (1b) [5]

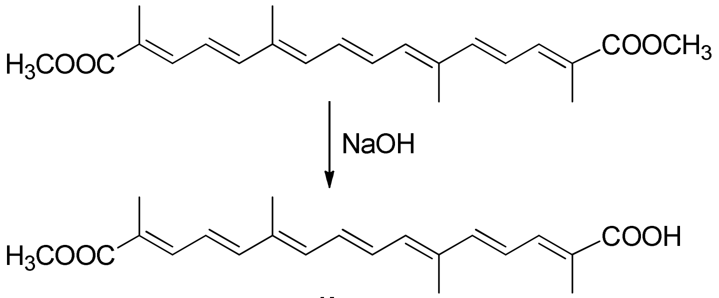

1b

Crocetin dimethyl ester (356 mg, 1 mmol) was dissolved in THF (5 ml) and NaOH (500 mg, 12,5 mmol) was added in methanol (16 ml). The solution was stirred for a week and then 5 % citric acid (50 ml) was added. The reaction mixture was washed with dichloromethane (3x50 ml), the organic phase was dried and evaporated. The product was purified by column chromatography (dichloromethane/methanol 9:1) to yield crocetin monomethyl ester **1b** (120 mg, 35%, HPLC purity 97%) as an orange powder and the starting material (72 mg).

M.p. 204-205 °C. UV (ethanol) 404, 427, 452 nm. HRMS (MALDI): *m/z* calcd for $C_{21}H_{26}O_4$+Na^+: 365.1723 [*M*+Na]$^+$; found: 365.1719.

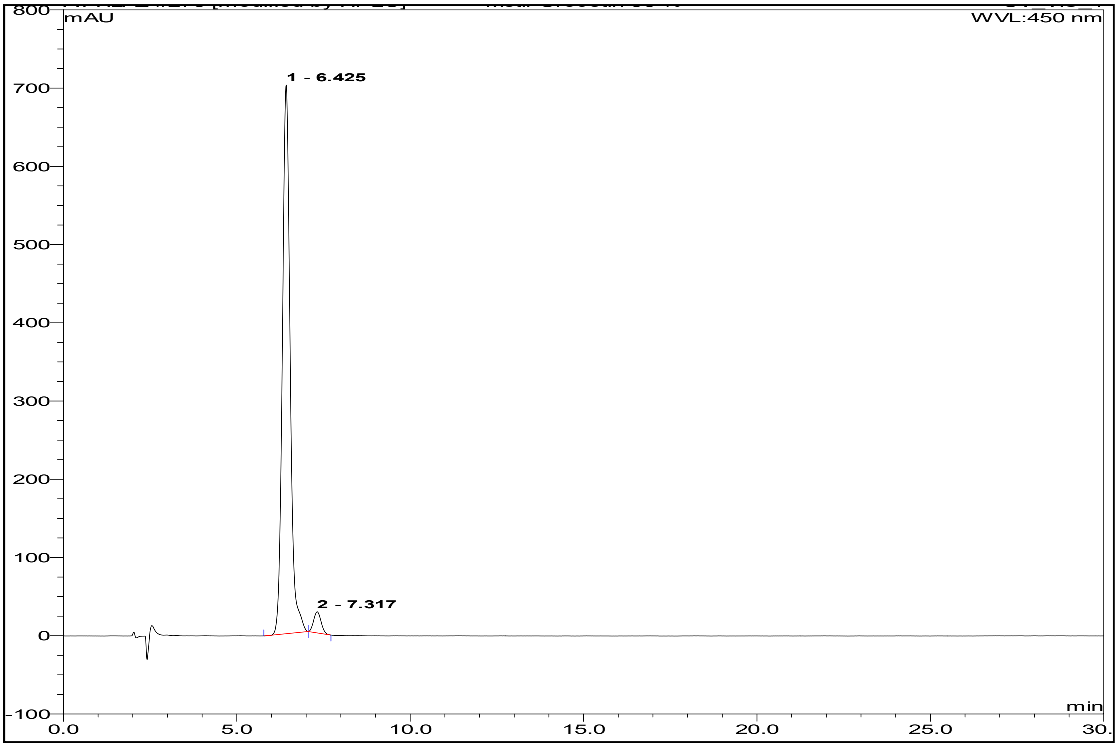

Figure S3. HPLC Chromatogram of crocetin monomethyl ester **1b**

Synthesis of β-apo-8'-carotenoic acid (1c)

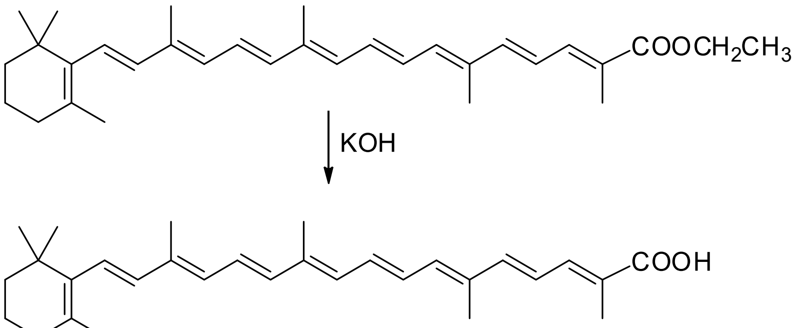

Ethyl apo-8'-carotenoate (Hoffman-La Roche, now DSM) (500 mg, 1.086 mmol) was dissolved in diethyl ether (200 ml) and saponified overnight with 30 % methanolic KOH solution (30 ml). On the next day 5% citric acid (300 ml) and diethyl ether (100 ml) were added to the reaction mixture and the phases were separated. The ethereal phase was washed twice with brine, dried, and evaporated. The crude acid was crystallized from toluene/hexane to yield **1c** (322 mg, 69%) as a dark red product. HPLC purity was 100%.

M.p. 194-195 C. UV (ethanol) 443, 463 nm. MS (MALDI-TOF): *m/z* calcd for $C_{30}H_{40}O_2+H^+$: 433 $[M+H]^+$; found: 433.

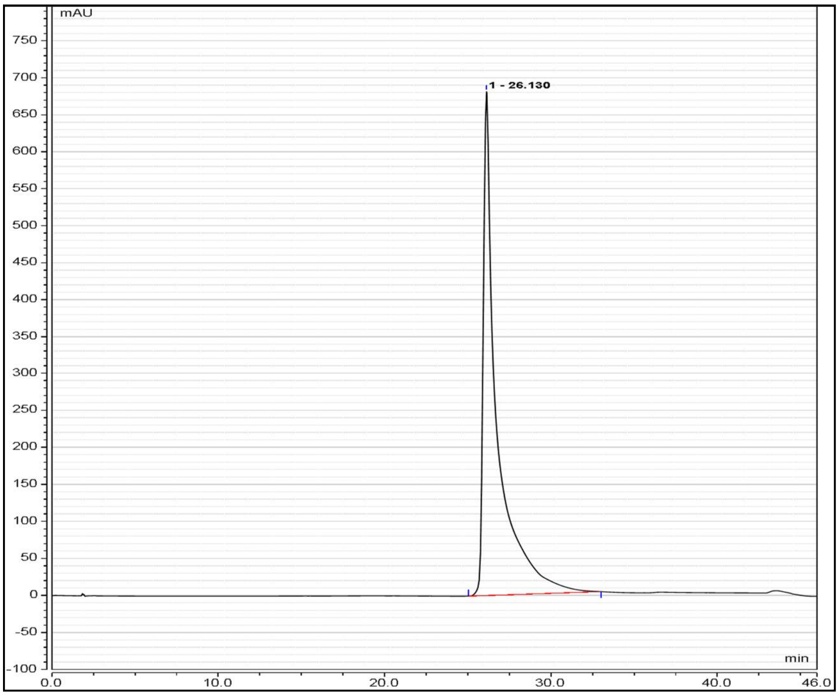

Figure S4. HPLC chromatogram of β-apo-8'-carotenoic acid **1c** at 450 nm. Eluents: (A) H_2O:MeOH = 12:88 v/v%, (B) MeOH and (C) Acetone:MeOH = 50:50 v/v%. The gradient program was the following:

0–2 min 100% A, 2–10 min to 80% A/20%B, 10–18 min to 50% A/50% B, 18–25 min to 100% B, 25–27 min 100% B, 27– 33 min to 100% C, 33–38 min 100% C, 38–40 min to 100% B (in linear steps). The flow rate was 1.25 ml/min, at 22 °C.

Synthesis of norbixin (1d)

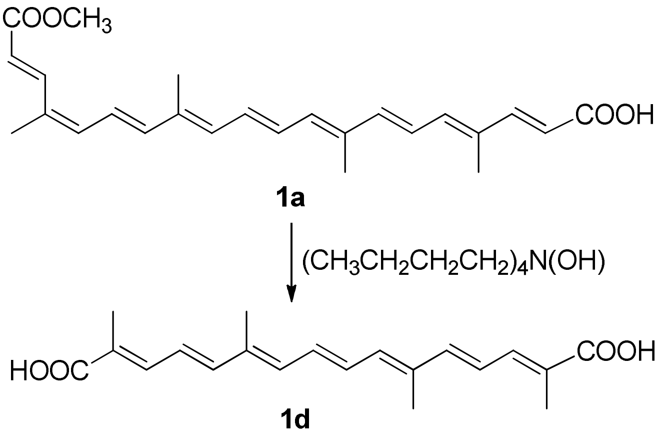

1.5 M aqueous tetrabutylammonium hydroxide solution (Acros, purum) (2.8 ml, 4.2 mmol) was added to a stirred (partial) solution of bixin (336 mg, 0.85 mmol) in THF (18 ml). The mixture was stirred at RT for 35 min (the reaction was monitored by TLC-analysis). Then the clear dark-red solution was quenched with 1 M aqueous acetic acid solution (10 ml, 10 mmol). The solution was seeded and stirred at RT for 15 min while the product began to crystallize. Water (25 ml) was added dropwise over 30 min. to the stirred suspension. The red precipitate was collected by filtration, washed with water (3 x 15 ml) and acetone (2 x 5 ml) and dried. Recrystallization from THF/EtOAc yielded **1d** (302 mg, 93%) as dark red crystals (m.p. >250 °C, HPLC purity 99 %). Physical and chromatographic properties were identical with those of our authentic sample and literature data [6].

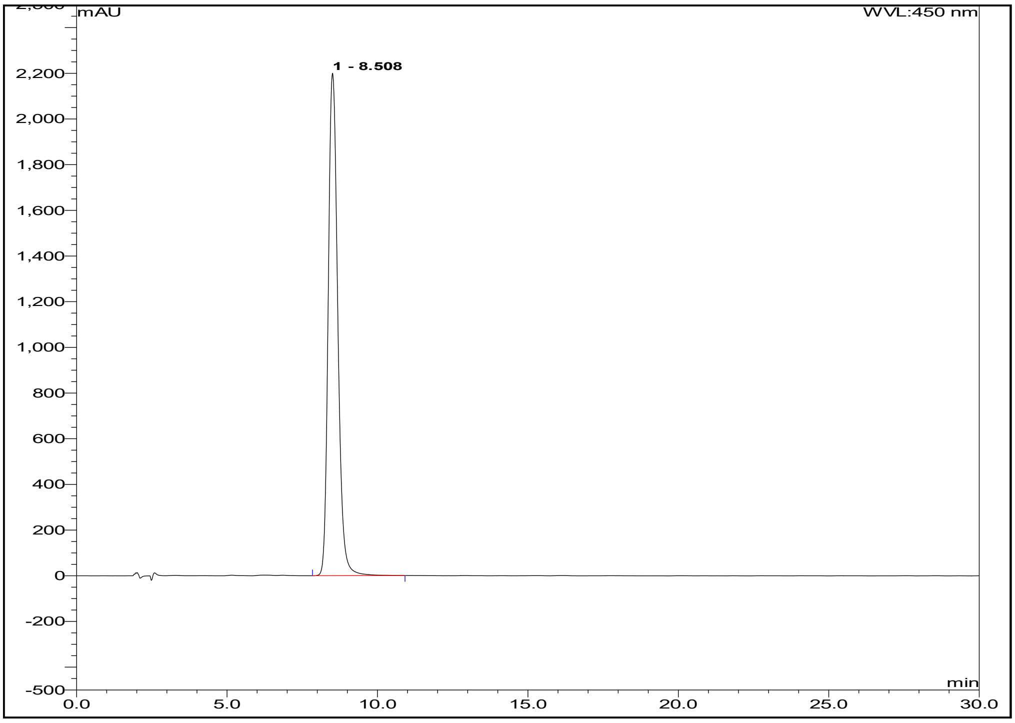

Figure S5. HPLC chromatogram of norbixin **1d**

General procedure for the synthesis of active esters 1a-ester, 1b-ester and 1c-ester

Compound **1a**, **1b** or **1c** (0.15 mmol) was dissolved in the mixture of dichloromethane (10 ml) and acetonitrile (2 ml). The mixture was cooled in an ice bath and *N*-hydroxysuccinimide (20 mg, 0.167 mmol) and *N*-(3-dimethylaminopropyl)-*N′*-ethylcarbodiimide hydrochloride (EDC) (31 mg, 0.16 mmol) were added. The reaction mixture was allowed to warm up to room temperature and stirred for 24 hours. The solvent was evaporated and the product was purified by flash column chromatography (hexane/EtOAc 7:3 for **1a-ester**; 6:4→1:1 for **1b-ester**, and 7:3 for **1c-ester**, respectively).

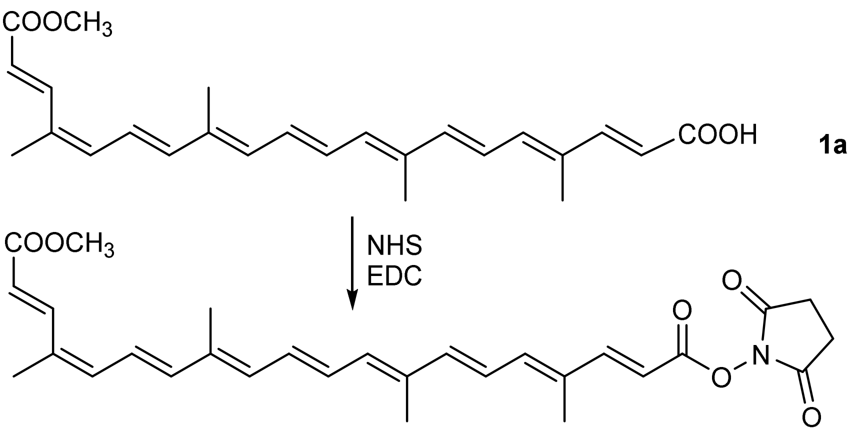

1a

1a-ester

1a-ester: yield 55.4 mg (76%) of dark red powder; R_f=0.33 (hexane/EtOAc 1:1); The product was used in the next step without NMR characterization; HRMS (MALDI): *m/z* calcd for $C_{29}H_{33}N_1O_6$+Na^+: 514.2200 [*M*+Na]$^+$; found: 514.2207.

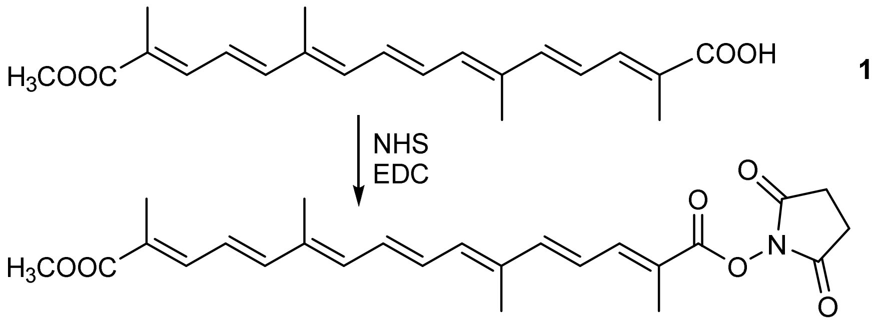

1b

1b-ester

1b-ester: yield 37.5 mg (57%) of orange powder; R_f=0.40 (hexane/EtOAc 1:1); The product was used in the next step without NMR characterization; HRMS (MALDI): *m/z* calcd for $C_{25}H_{29}N_1O_6$+Na^+: 462.1887 [*M*+Na]$^+$; found: 462.1890.

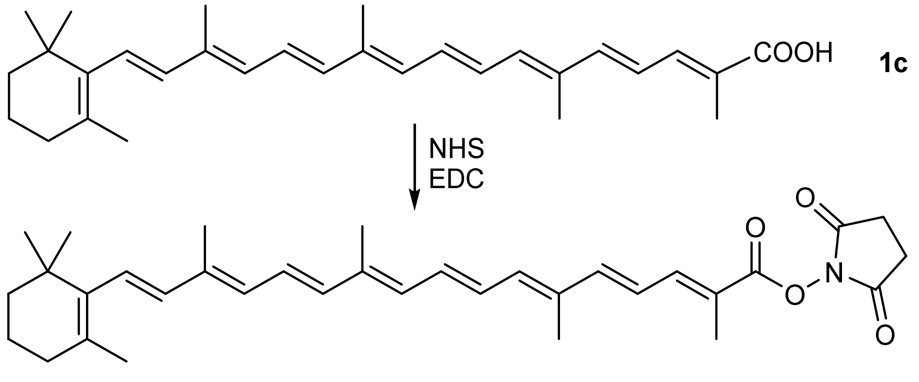

1c

1c-ester

1c-ester: yield 56 mg (71%) of red powder; R_f=0.42 (hexane/EtOAc 1:1); HRMS (MALDI): *m/z* calcd for $C_{34}H_{43}N_1O_4$+Na^+: 552.3084 [*M*+Na]$^+$; found: 552.3159.

General procedure for the synthesis of apocarotenoid-glycopeptide conjugates 5a, 5b, 5c, 6, 7

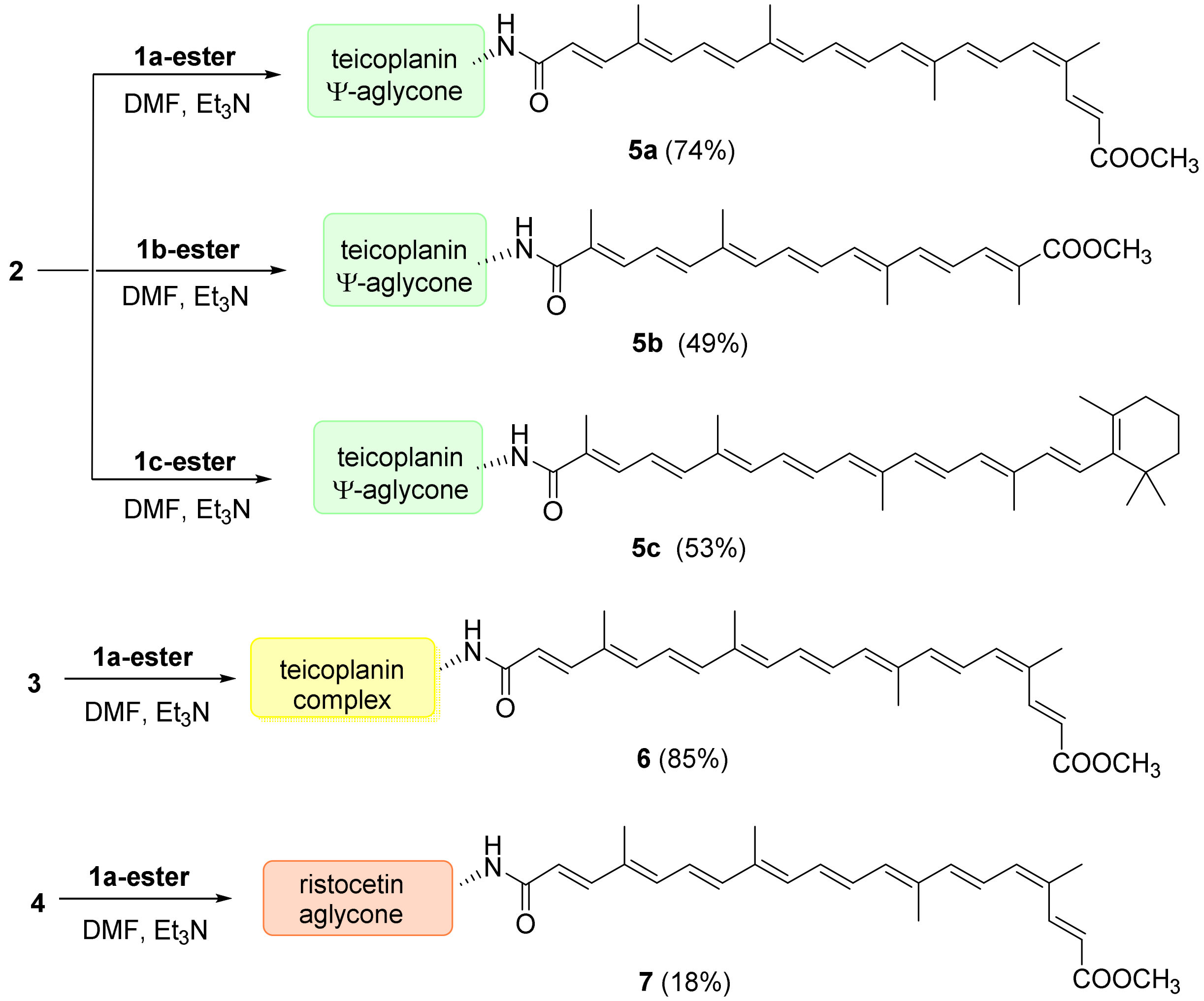

Scheme S1. Acylation of the *N*-terminal amino group of glycopeptide derivatives **2-4** by apocarotenoid active esters of **1a-1c**

Glycopeptide derivative (**2**, **3** or **4**) (0.05 mmol) was dissolved in dimethylformamide (2 ml) and triethylamine (7 µl, 0.05 mmol) and apocarotenoid active ester (**1a-ester**, **1b-ester** or **1c-ester)** (0.075 mmol) was added. The reaction mixture was stirred overnight, then the solvent was evaporated and the product was purified by flash column chromatography (toluene/MeOH 7:3→1:1 for **5a**, **5b**, **5c** and **7**; acetonitrile/H_2O 95:5→9:1 for **6**, respectively).

5a: yield 65.5 mg (74%, HPLC purity 97.4%), dark red powder; R_f=0.28 (toluene/MeOH 1:1); HRMS (ESI): *m/z* calcd for $C_{91}H_{85}Cl_2N_8O_{26}Na+Na^+$: 1821.4742 [*M*-H+2Na]$^+$; found: 1821.4742.

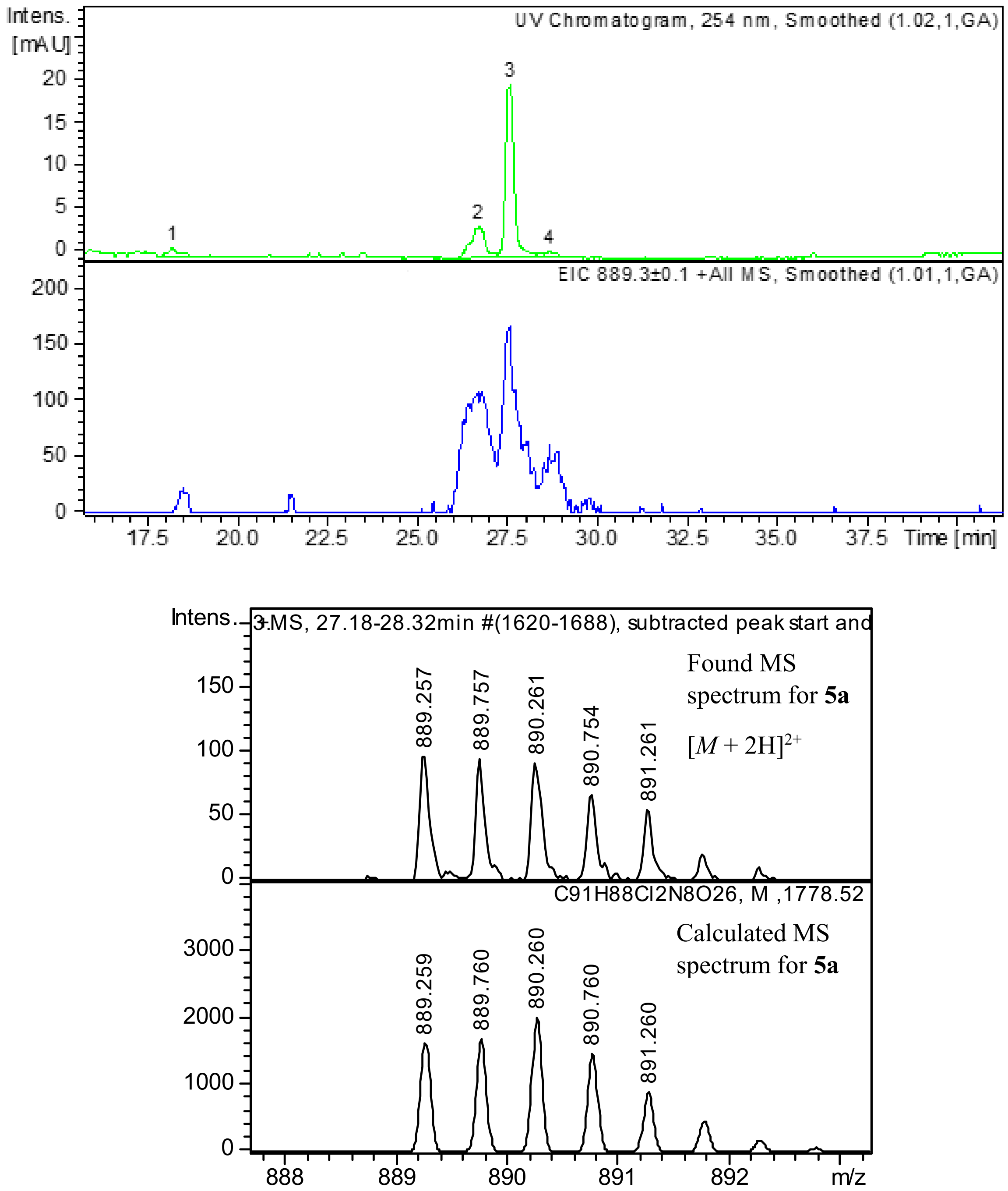

Peak	RT [min]	Area	Area Frac. %	Sum of isomers %
1	18.15	11.244	2.6	
2	26.67	97.080	22.3	97.4
3	27.53	312.318	71.8	
4	28.63	14.171	3.3	

Figure S6. HPLC-MS of compound **5a**.

5b: yield 43 mg (51%, HPLC purity 96.1%), orange powder; R_f=0.27 (toluene/MeOH 1:1); HRMS (ESI): *m/z* calcd for $C_{87}H_{82}Cl_2N_8O_{26}Na+Na^+$: 1769.4429 [*M*-H+2Na]$^+$; found: 1769.4428;

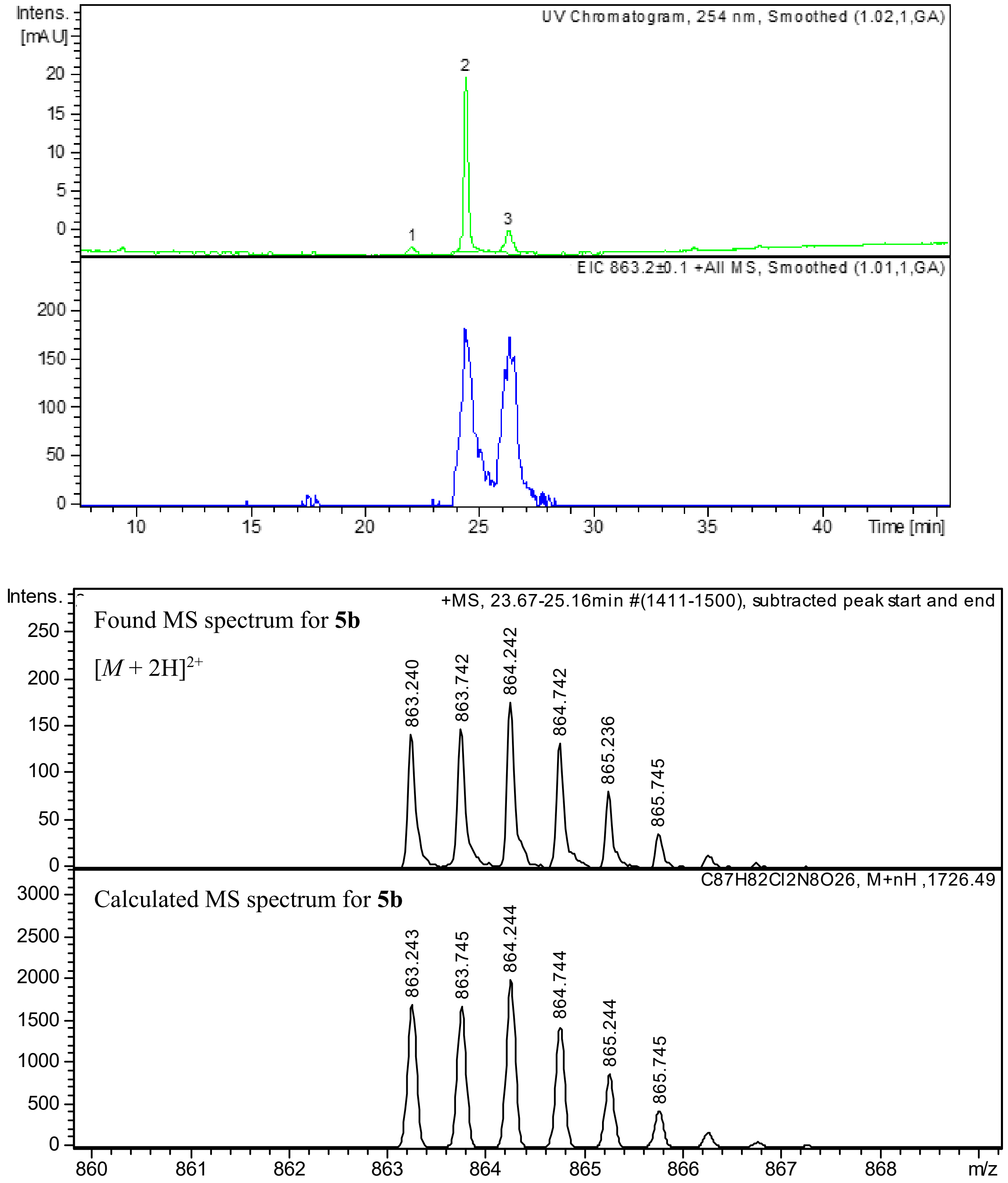

Peak	RT [min]	Peak area	Area Frac. %	Sum of isomers %
1	22.00	16	3.9	
2	24.36	325	78.9	96.1
3	26.26	71	17.2	

Figure S7. HPLC-MS of compound **5b**.

5c: yield 47.5 mg (53%, HPLC purity 96.6%), red powder; R_f=0.29 (toluene/MeOH 1:1); HRMS (ESI): *m/z* calcd for $C_{96}H_{95}Cl_2N_8O_{24}+Na^+$: 1859.5626 [*M*-H+2Na]$^+$; found: 1859.5627;

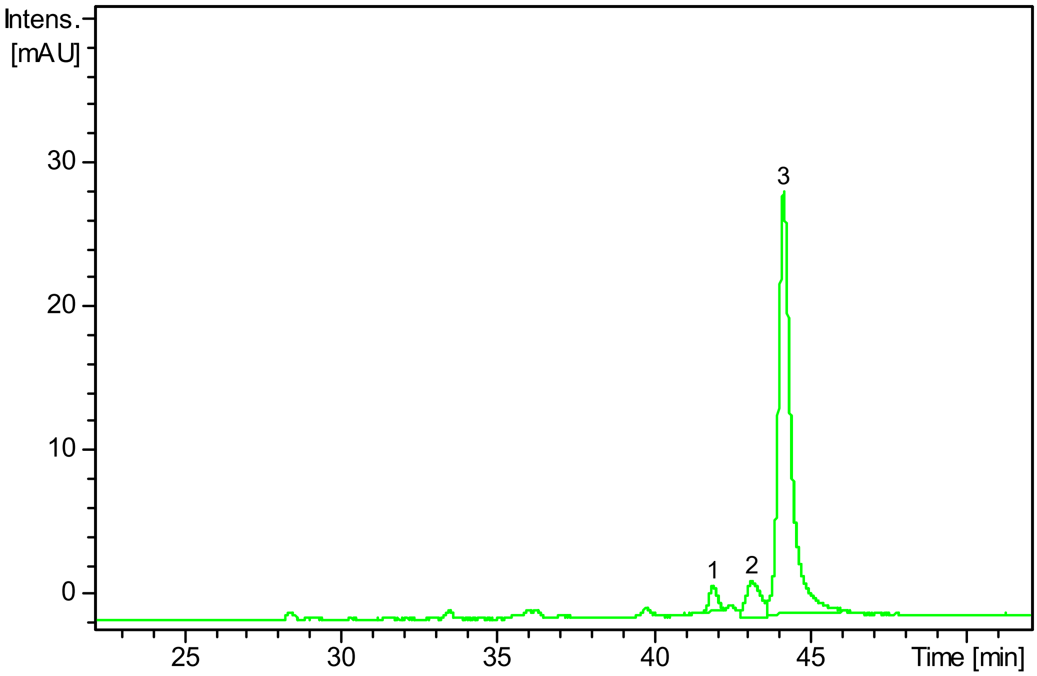

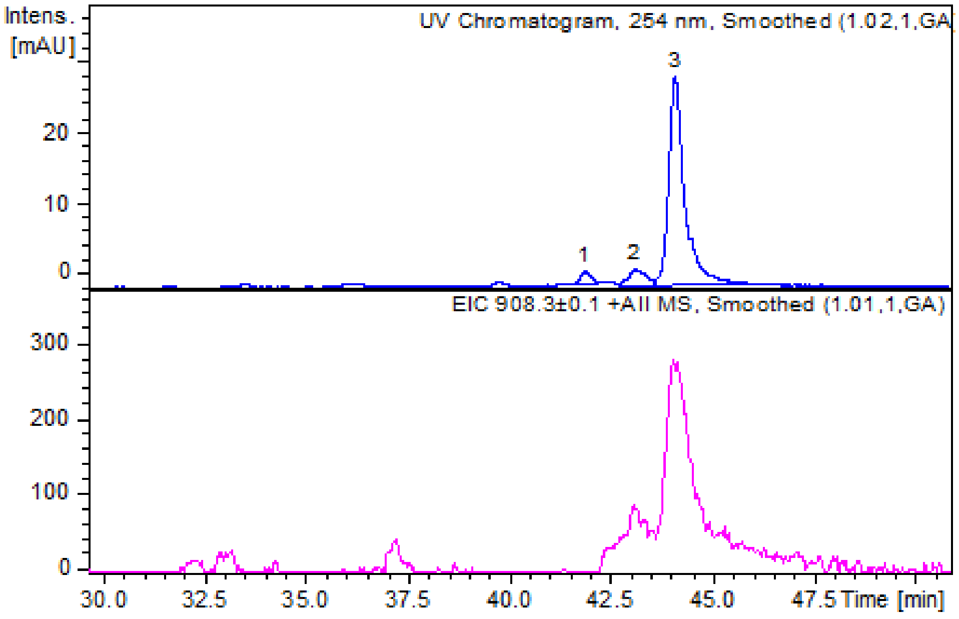

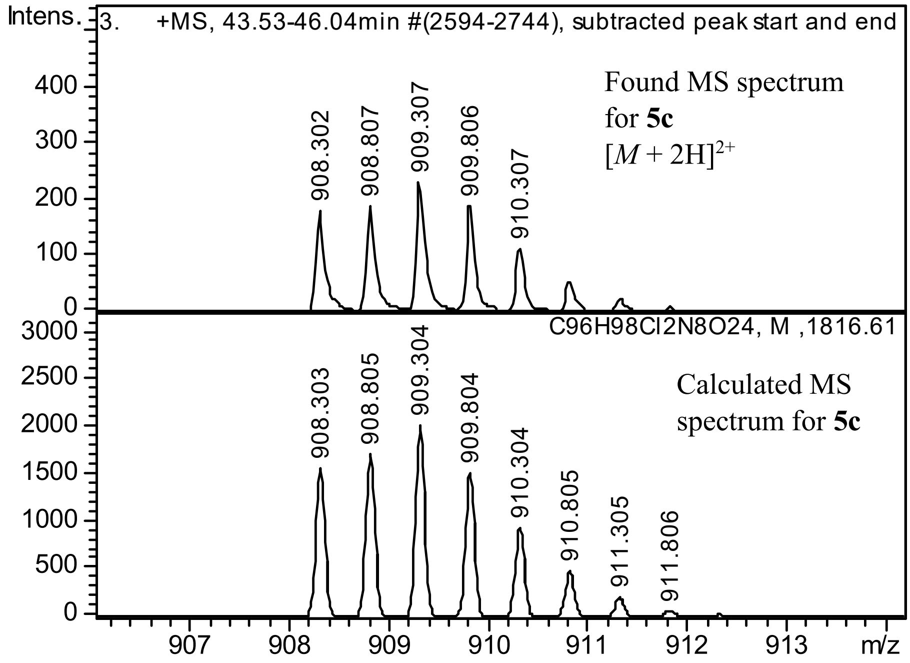

Peak	RT [min]	Area	Area Frac. %	Sum of isomers %
1	41.82	31.3	3.4	
2	43.03	82.5	8.8	96.6%
3	44.04	816.3	87.8	

Figure S8. HPLC-MS of compound **5c**.

6: yield 96 mg (85%), red powder; R_f=0.32 (acetonitrile/H_2O 85:15); RP-HPLC-ESI MS for the main peak (63.2%): RT = 26.97 min, *m/z* calcd for $C_{113}H_{125}Cl_2N_9O_{36}+2H^+$: 1127.887 $[M+2H]^{2+}$; found: 1127.887;

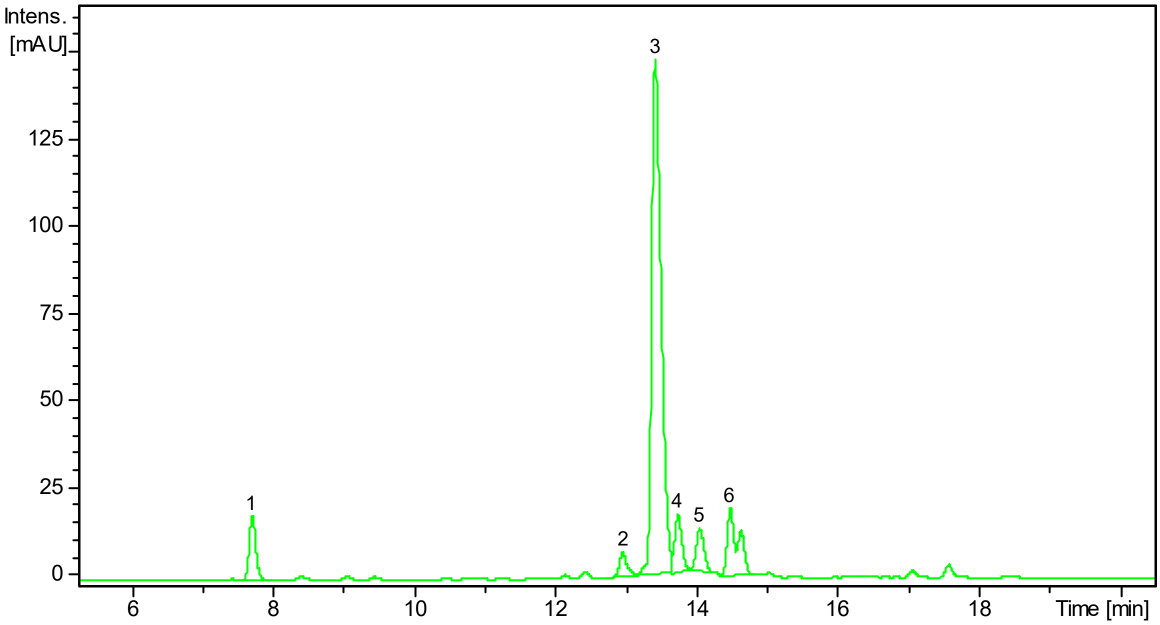

Peak	RT [min]	Area	m/z	Area Frac. %	Compound
1	7.73	135.510	782.190	6.2	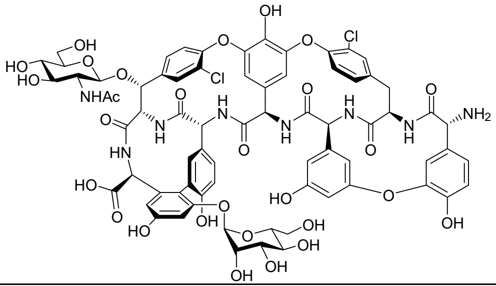
2	12.94	56.376	938.785	2.6	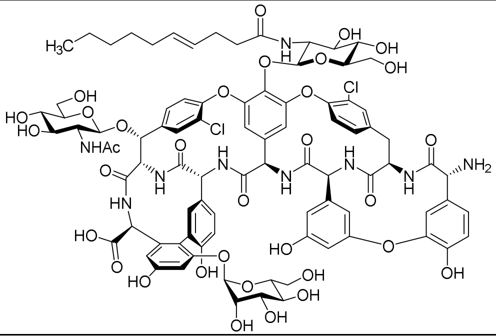

3	13.40	1514.511	939.795	69.8	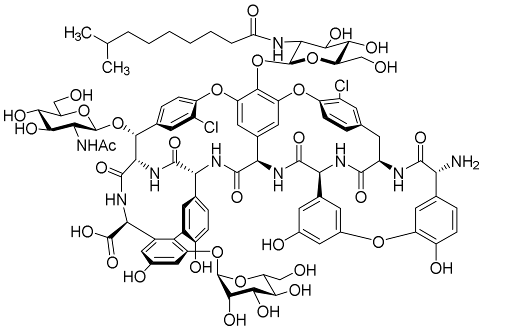 or
4	13.72	131.641	939.787	6.1	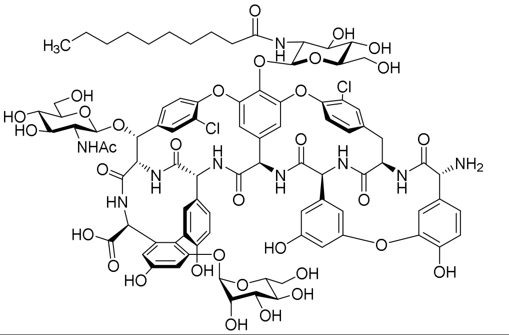
5	14.03	102.122	858.769	4.7	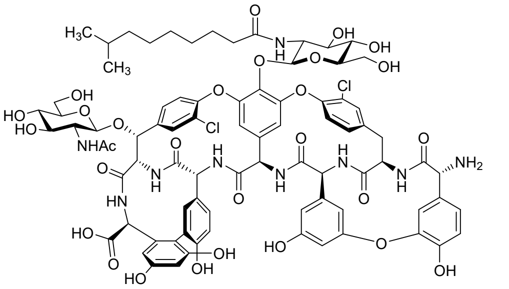 or 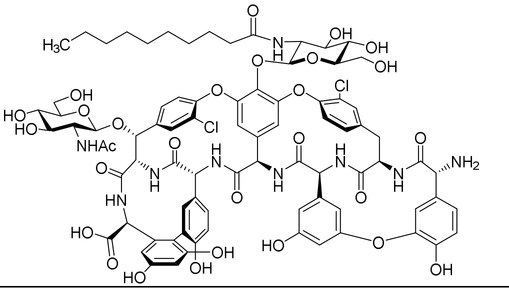
6	14.46	229.217	946.804	10.6	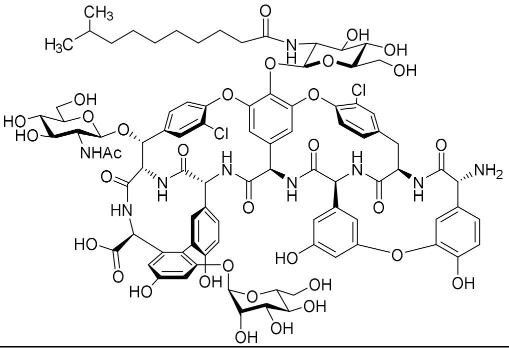

Figure S9. HPLC-MS of teicoplanin (**3**)

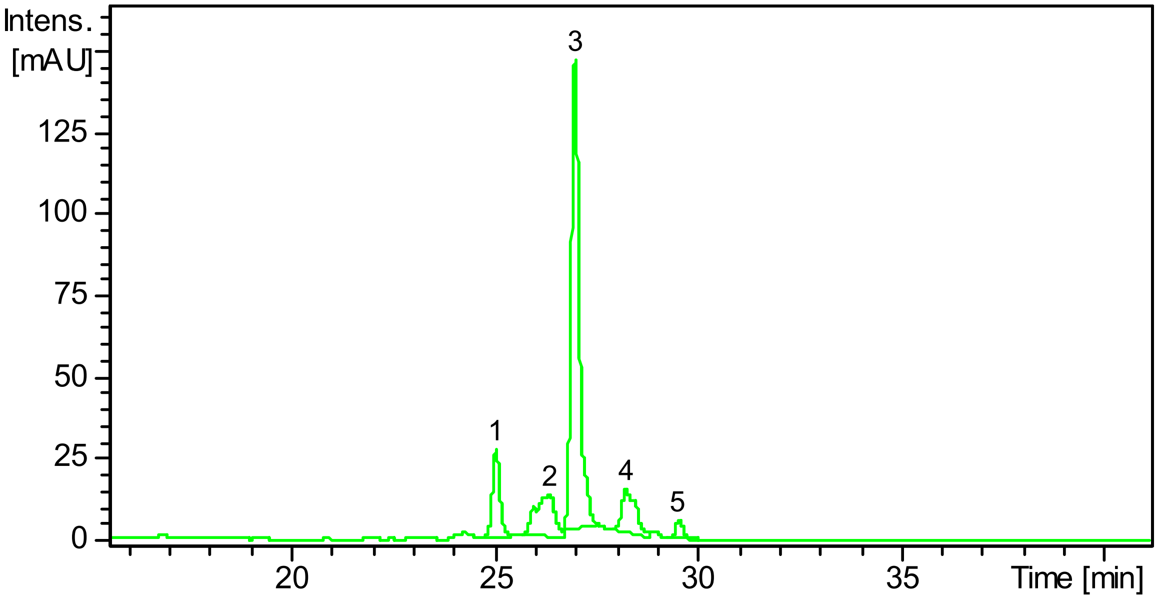

Peak	RT [min]	Area	m/z	Area Frac. %	Compound
1	25.05	338.861	970.289	10.9	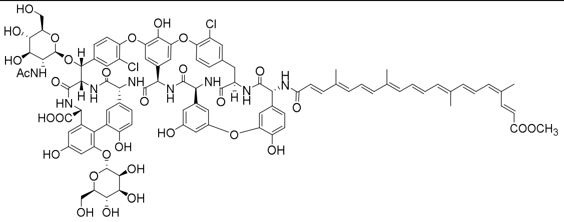
2	26.35	415.338	1127.889	13.3	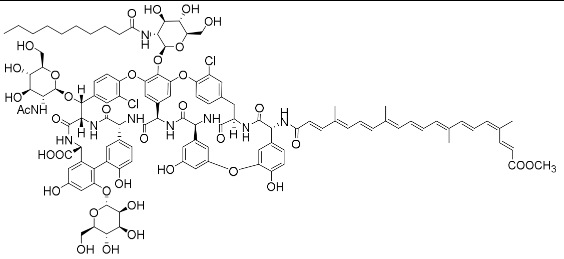 and
3	26.97	1973.513	1127.887	63.2	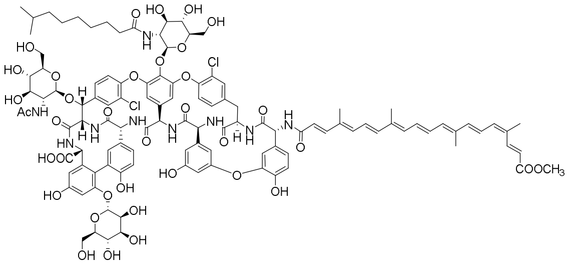
4	28.21	315.676	1134.896	10.1	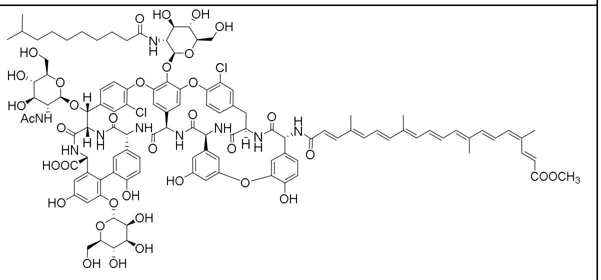

					or 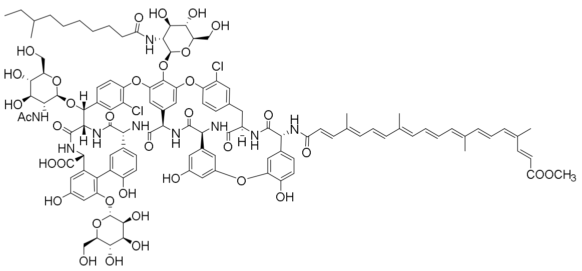
5	29.50	77.329	1046.860	2.5	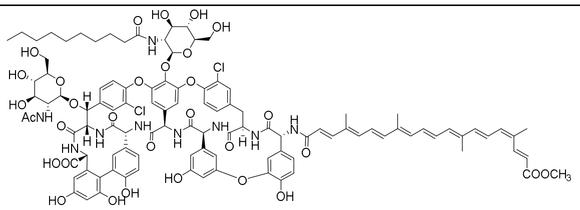

Figure S10. HPLC-MS of compound **6**

7: yield 14 mg (18%, HPLC purity 95.7%) red powder; R_f=0.51 (toluene/MeOH 1:1); HRMS (MALDI): *m/z* calcd for $C_{85}H_{79}N_7O_{22}$+Na^+: 1572.5176 [*M*+Na]$^+$; found: 1572.5670.

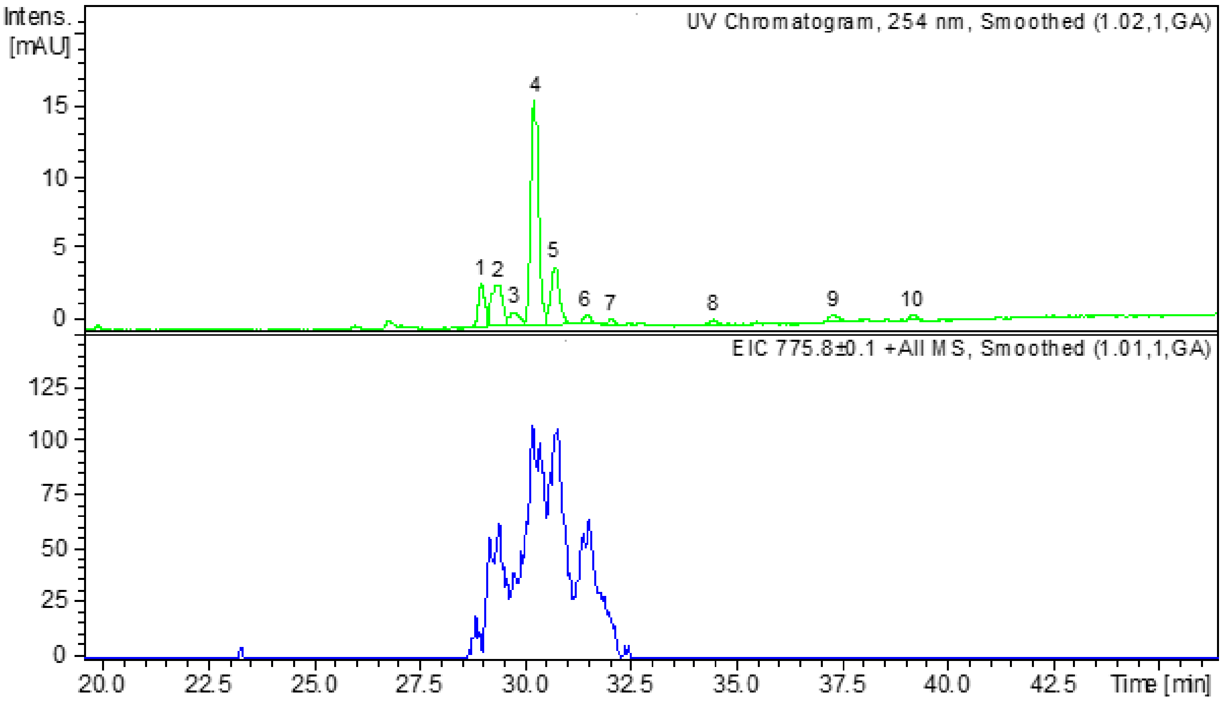

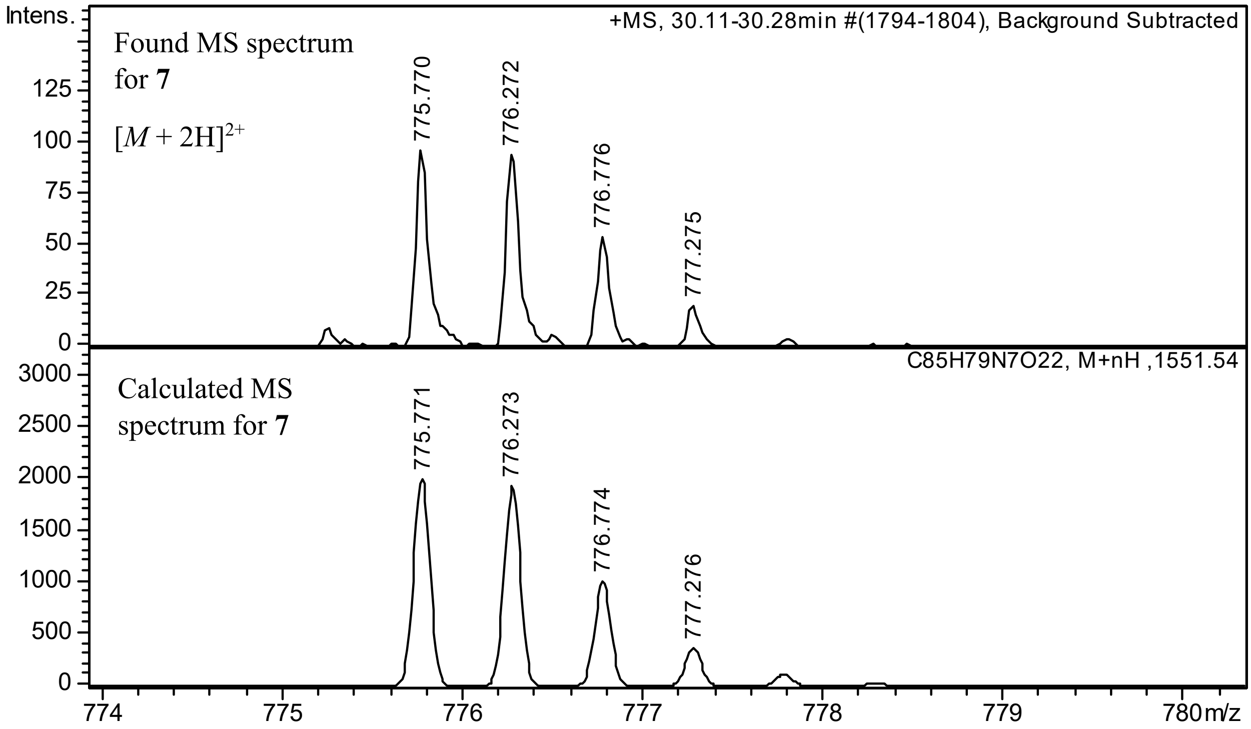

Peak	RT [min]	Peak area	Area Frac. %	Sum of isomers %
1	28.92	35.5	9.5	95.7
2	29.30	53.2	14.2	
3	29.69	16.7	4.5	
4	30.19	182.8	48.8	
5	30.67	56.0	15.0	
6	31.40	9.0	2.4	
7	32.01	4.7	1.3	
8	34.42	4.4	1.2	
9	37.24	7.3	2.0	
10	39.13	4.8	1.3	

Figure S11. HPLC-MS of **7**.

Synthesis of 9

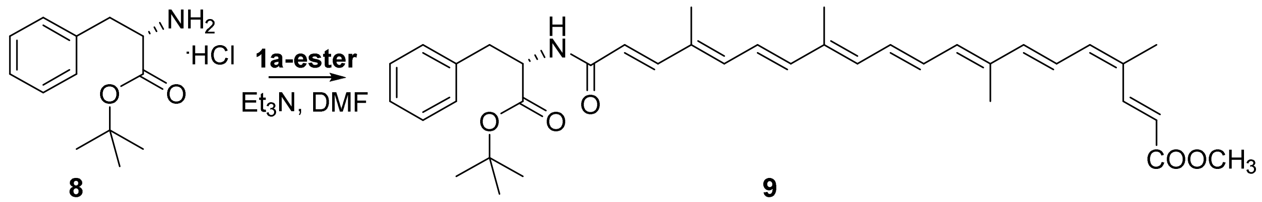

tert-Butyl L-phenylalaninate (52 mg, 0.2 mmol) was dissolved in abs. dimethylformamide (2 ml) and 4-methylmorpholine (18 µl, 0.22 mmol) was added and the mixture was stirred for 30 minutes. Under argon athmosphere, **1a-ester** (49 mg, 0.1 mmol) was added and the reaction mixture was stirred overnight. The solvent was evaporated and the product was purified by flash column chromatography (hexane/EtOAc 7:3→6:4) to yield **9** (40 mg, 67%, HPLC purity 95.9%) as a dark red powder. R_f=0.56 (hexane/EtOAc 7:3). HRMS (ESI): *m/z* calcd for $C_{38}H_{47}NO_5+H^+$: 598.353 $[M+H]^+$; found: 598.351.

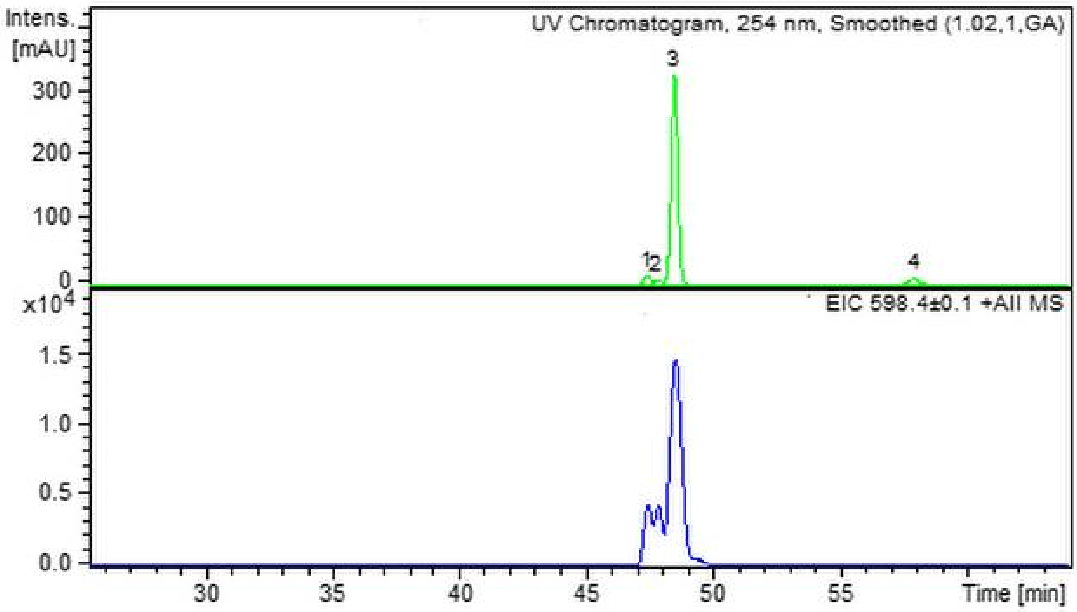

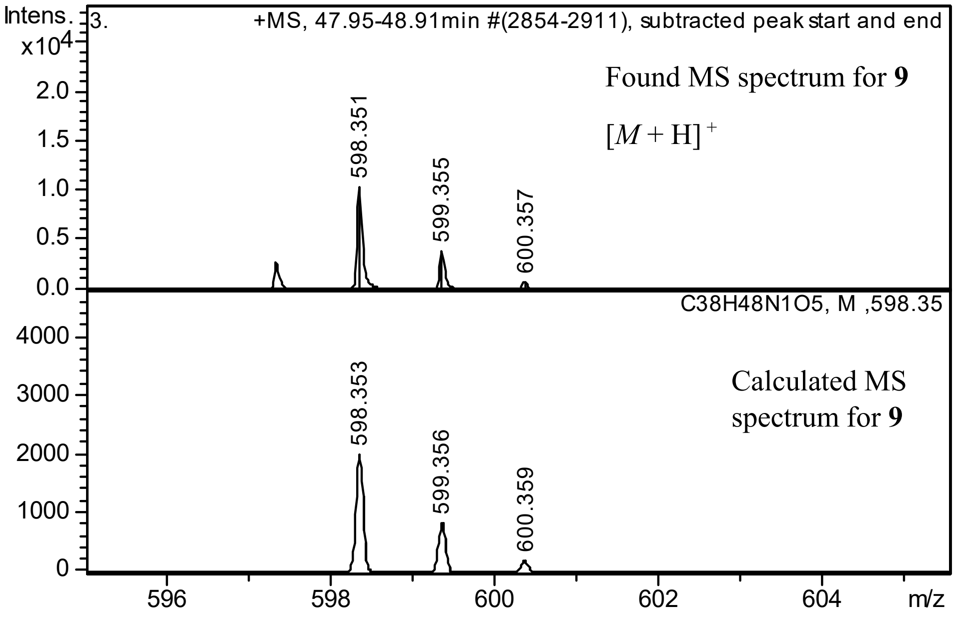

Peak	RT [min]	Area	Area Frac. %	Sum of isomers %
1	47.31	254.7	3.9	95.9
2	47.72	134.3	2.1	
3	48.41	5821.7	89.9	
4	57.77	264.0	4.1	

Figure S12. HPLC-MS of compound **9**.

Antiviral activity determination based on viral RNA reduction assay

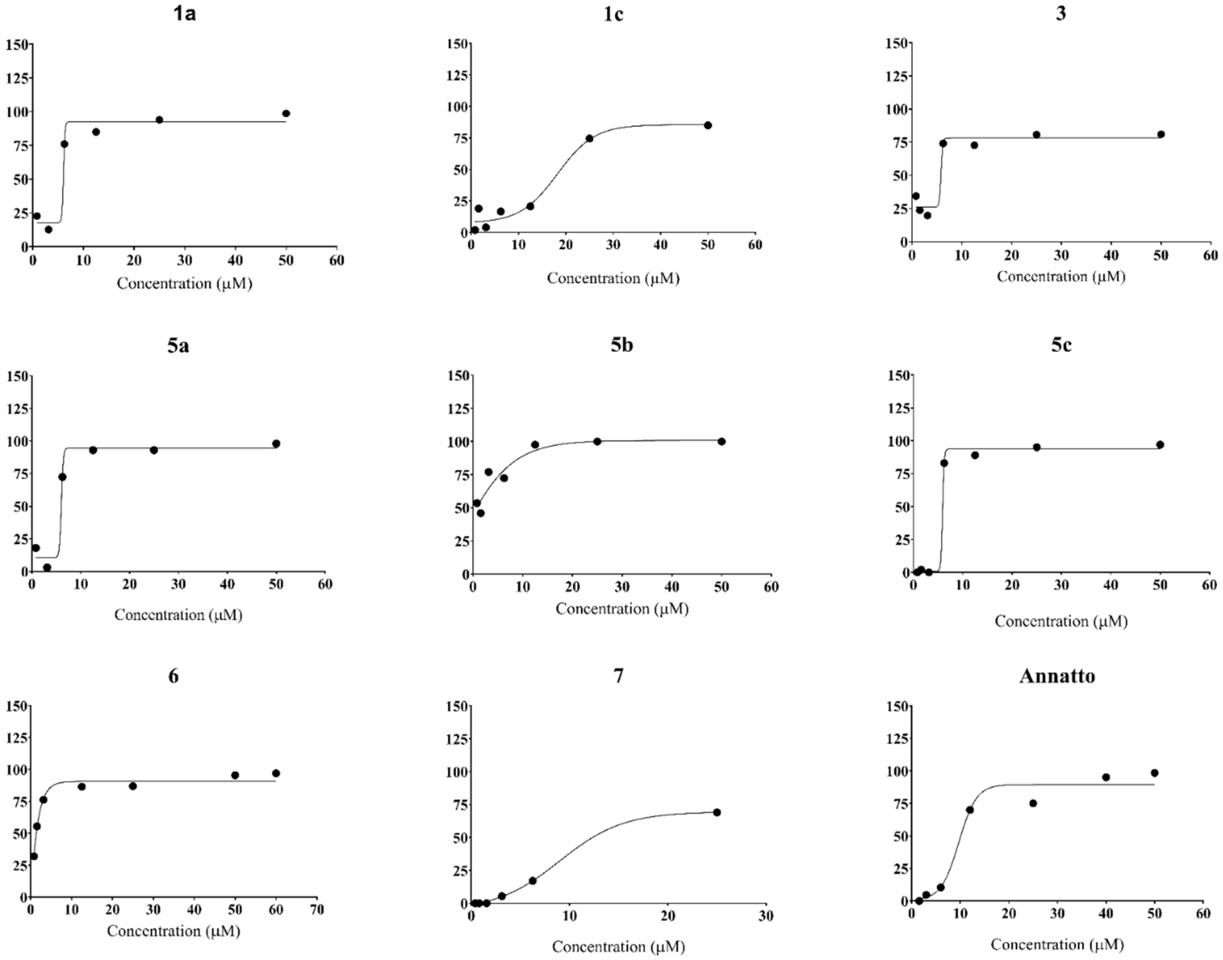

Figure S13. Inhibition of SARS-CoV-2 replication in Vero E6 cells by different doses of apocarotenoids (**1a** and **1b**), teicoplanin (**3**) teicoplanin derivatives (**5a**-**5c** and **6**), ristocetin derivative (**7**) and annatto. Vero E6 cells were infected with SARS-CoV-2 at an MOI of 0.01 for 48 h; and treatment were done at the same time. The viral yield in cell supernatant was quantified by ddPCR.

Antiviral activity determination using CPE-based assay

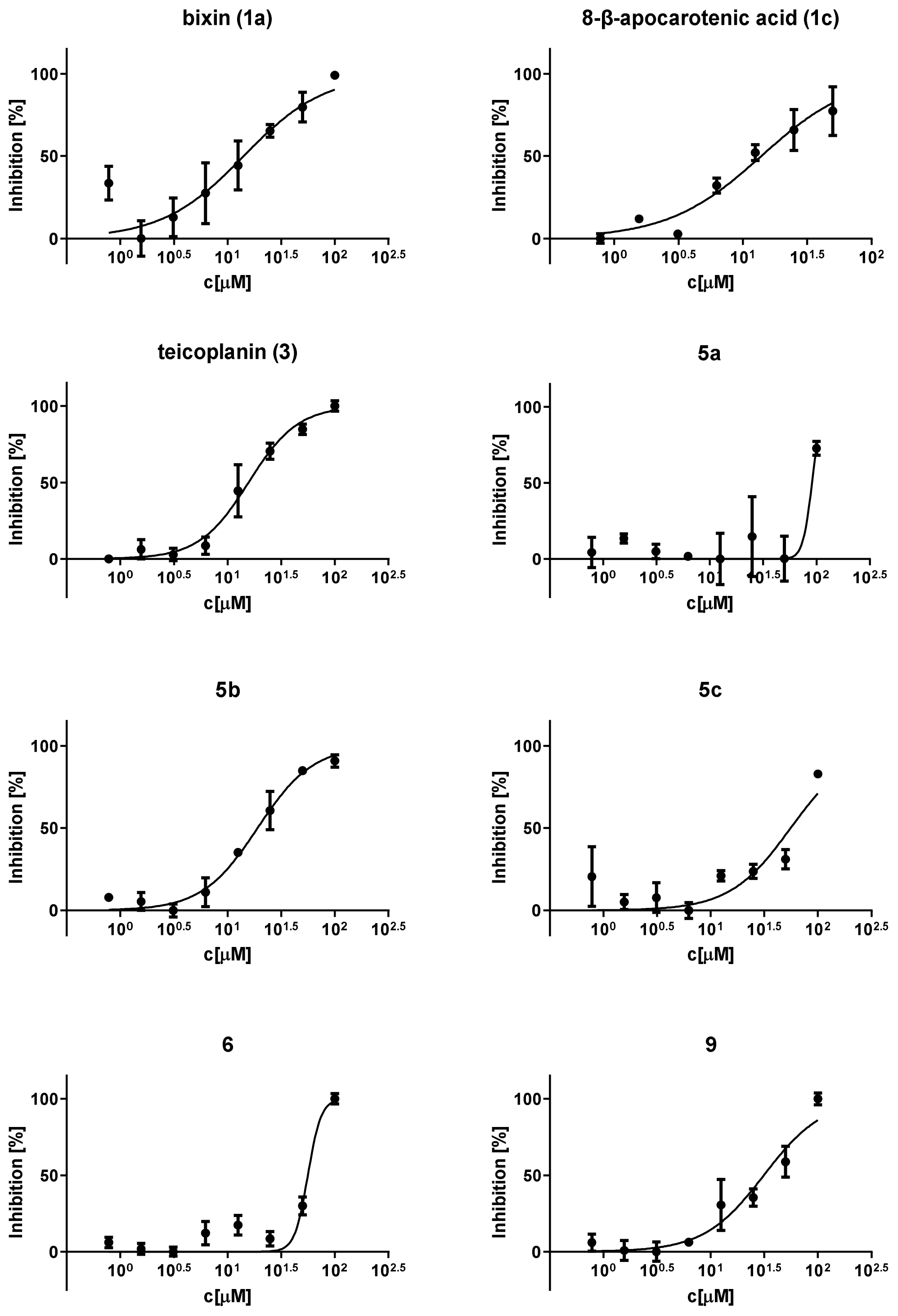

Figure S14. Inhibition of cytopathic effect of SARS-CoV-2 (MOI 0.04) in Vero E6 cells by different doses of apocarotenoids (**1a** and **1b**), teicoplanin (**3**), teicoplanin derivatives (**5a**-**5c** and **6**), and bixinoyl phenylalanine derivative (**9**). Vero E6 cells were infected with SARS-CoV-2 at an MOI of 0.04 for 72 h and the inhibition of virus-induced cytopathic effect was determined by XTT assay.

Antiviral activity determination using immunofluorescence assay (IFA)

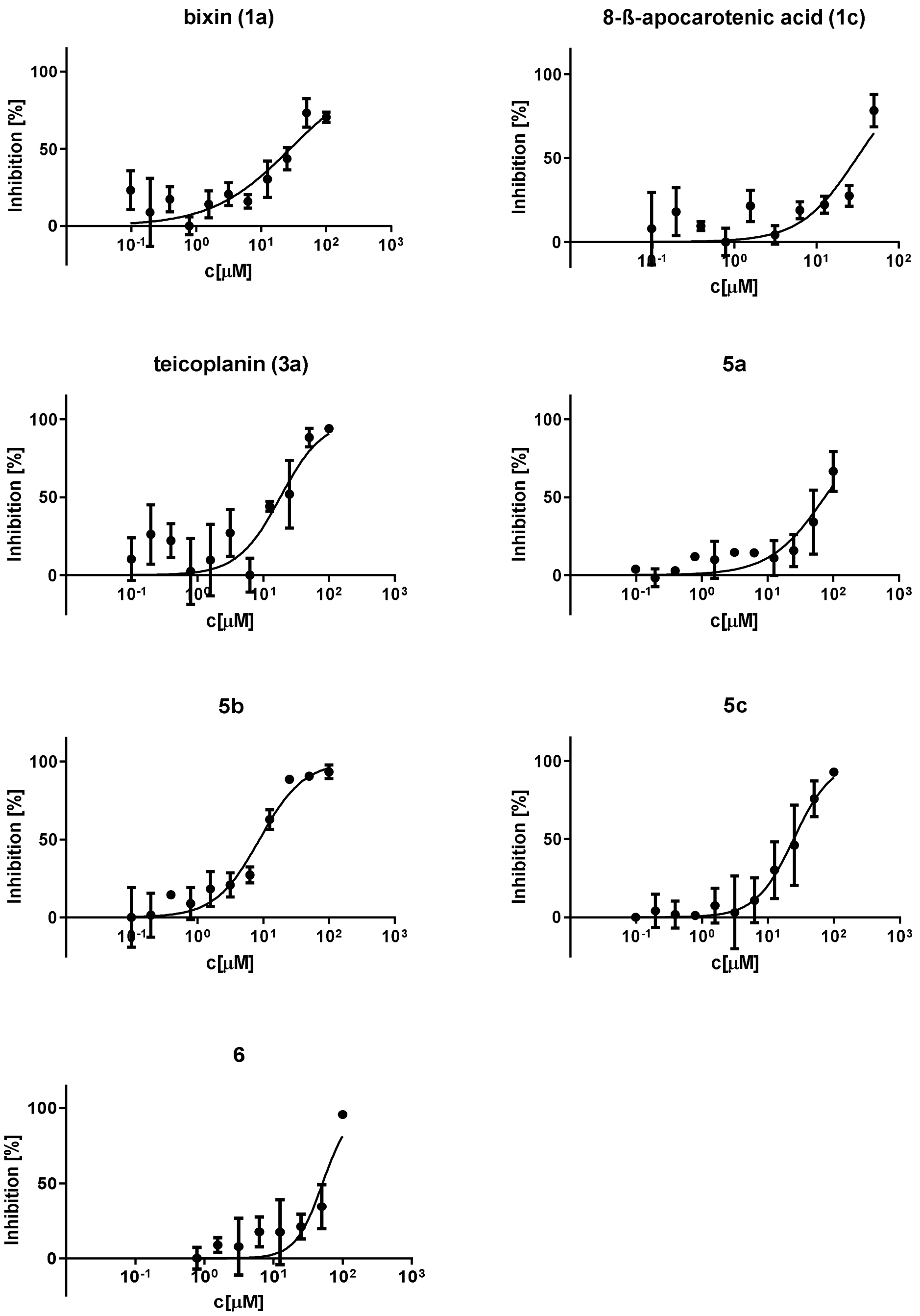

Figure S15. Inhibition of SARS-CoV-2 replication in Vero E6 by different doses of apocarotenoids (**1a** and **1c**), teicoplanin (**3**) and teicoplanin derivatives (**5a**-**5c** and **6**) detemined by IFA. Vero E6 cells were infected with SARS-CoV-2 at an MOI of 0.04 for 72 h and the inhibition of virus replication was analyzed by anti-SARS-CoV-2 nucleoprotein antibody.

SARS-CoV-2 DAPI MERGE

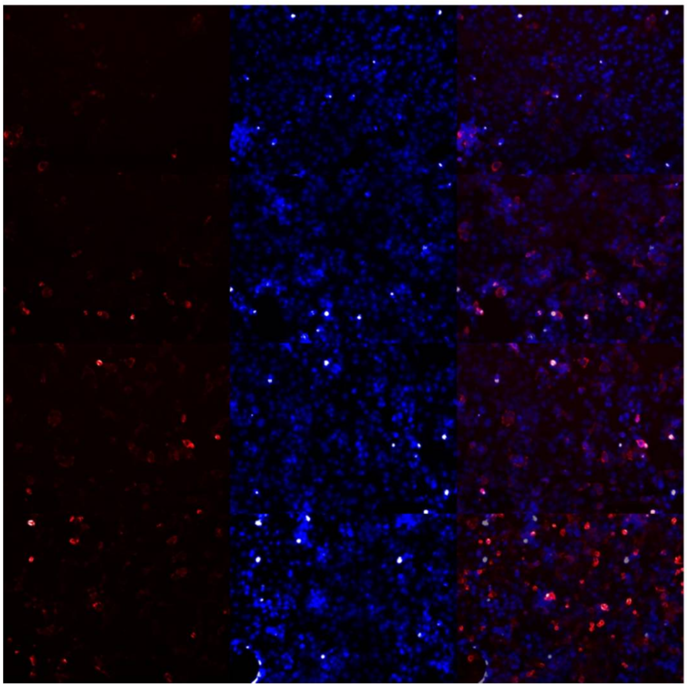

Bixin (1a), 100 µM

Bixin (1a), 12.5 µM

Bixin (1a), 0.78 µM

Bixin (1a), 0.098 µM

SARS-CoV-2 DAPI MERGE

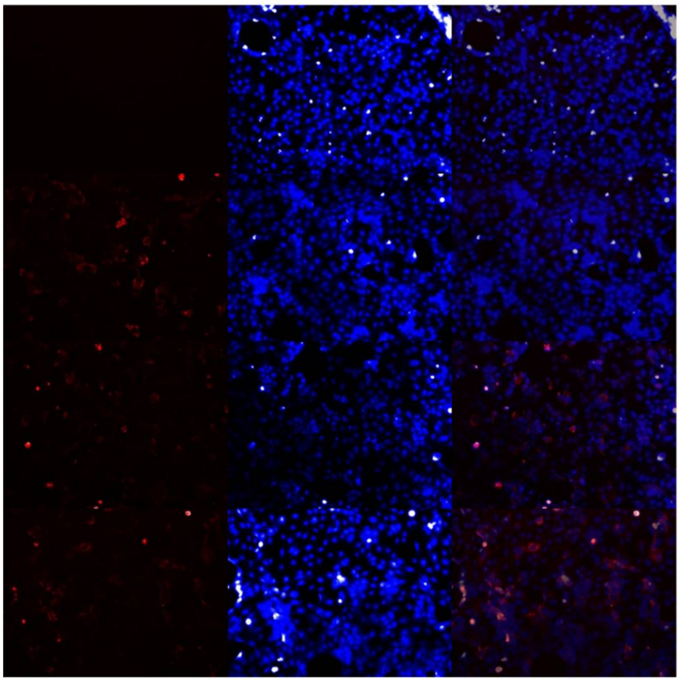

8-β-apocarotenic acid (1c), 100 µM

8-β-apocarotenic acid (1c), 12.5 µM

8-β-apocarotenic acid (1c), 0.78 µM

8-β-apocarotenic acid (1c), 0.098 µM

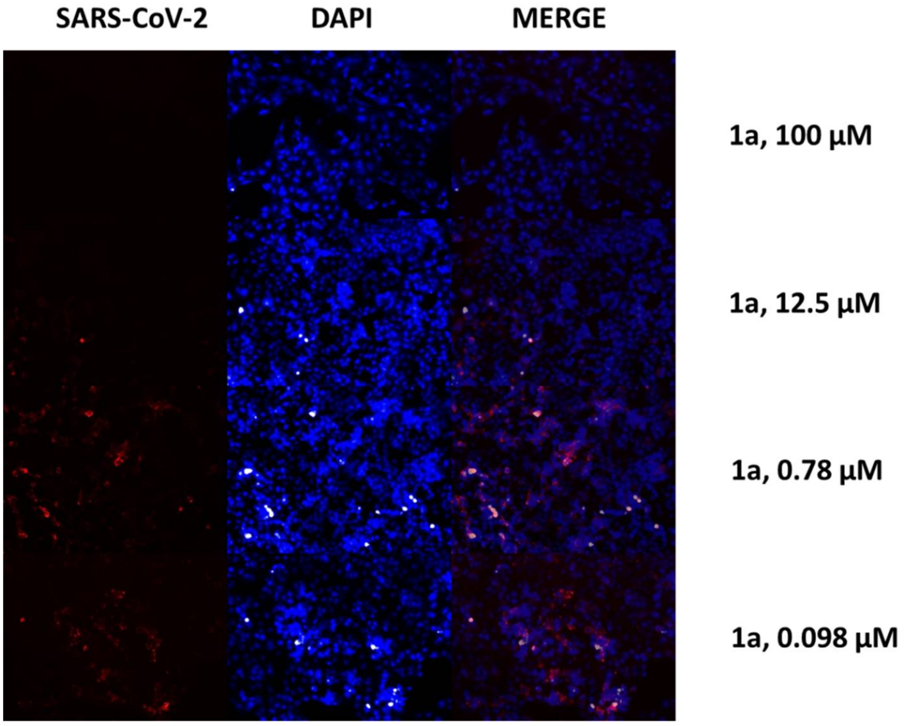
SARS-CoV-2
DAPI
MERGE
1a, 100 μM
1a, 12.5 μM
1a, 0.78 μM
1a, 0.098 μM

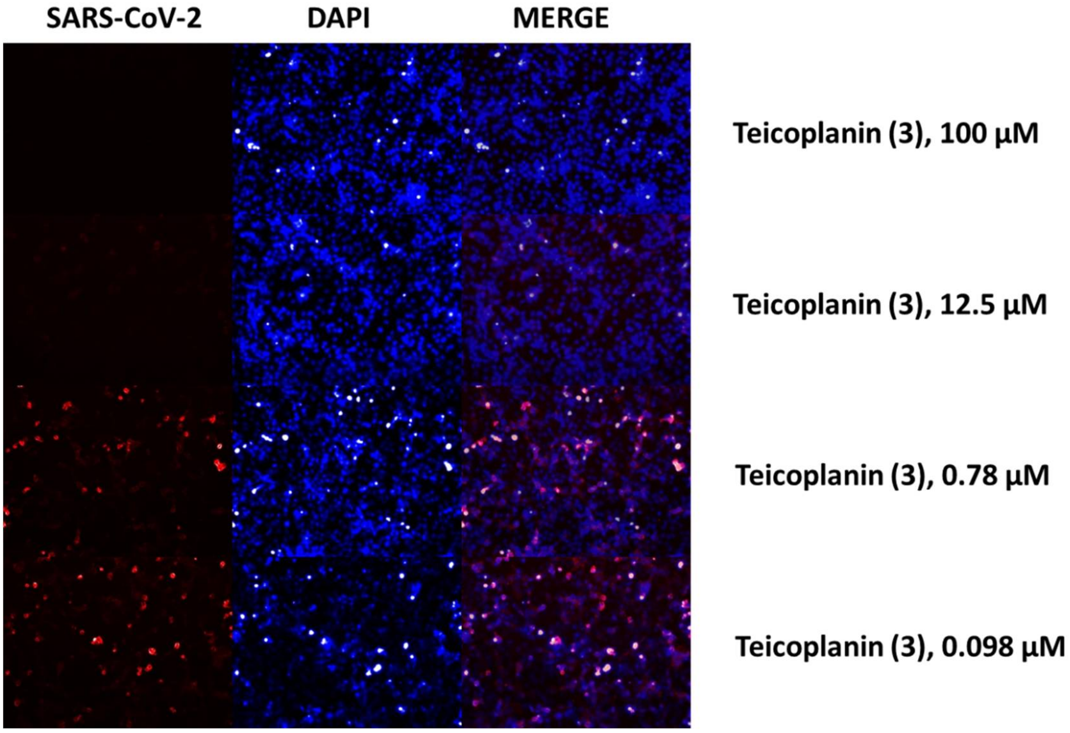
SARS-CoV-2
DAPI
MERGE
Teicoplanin (3), 100 μM
Teicoplanin (3), 12.5 μM
Teicoplanin (3), 0.78 μM
Teicoplanin (3), 0.098 μM

SARS-CoV-2 DAPI MERGE

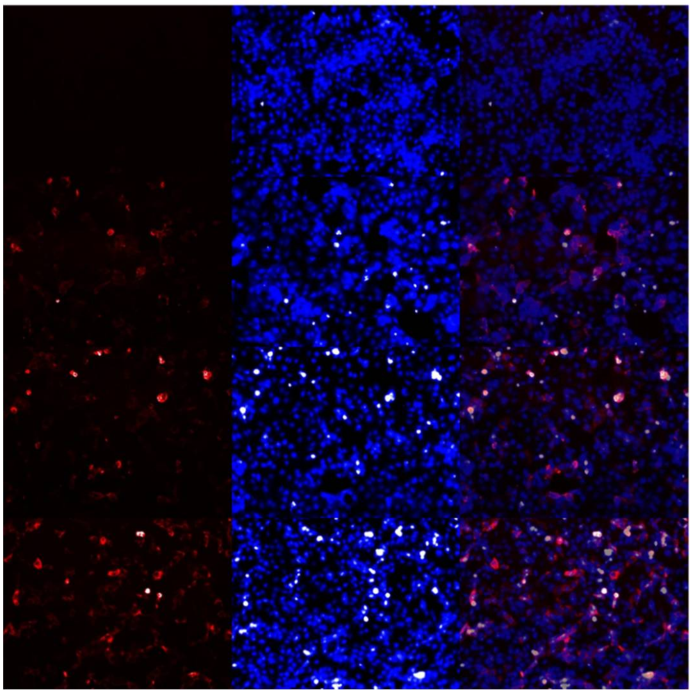

5a, 100 μM

5a, 12.5 μM

5a, 0.78 μM

5a, 0.098 μM

SARS-CoV-2 DAPI MERGE

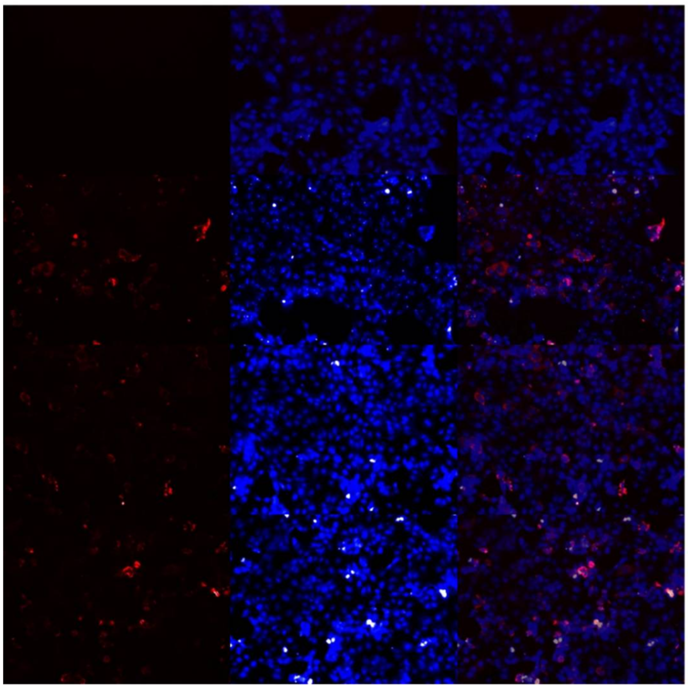

5c, 100 μM

5c, 12.5 μM

5c, 0.78 μM

5c, 0.098 μM

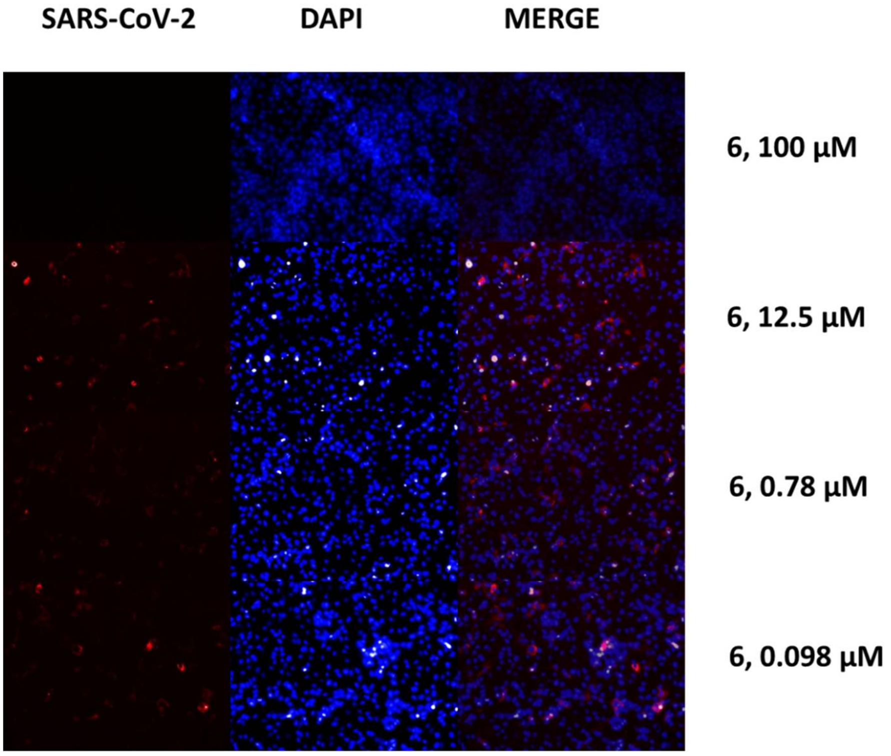

Figure S16. Immunofluorescence microscopy analysis of SARS-CoV-2 infection after addition of 100 μM, 12.5 μM, 0.78 μM, and 0.098 μM of apocarotenoids (**1a** and **1c**), teicoplanin (**3**), and teicoplanin derivatives (**5a**-**5c** and **6**). The infected cells were visualized using mouse SARS-CoV-2 nucleoprotein antibody followed by Cy3-conjugated anti-mouse antibody. The nuclei were stained with DAPI (Invitrogen).

Cathepsin inhibition assays

Human recombinant cathepsins B and L were expressed in *Escherichia coli* [7,8]. The assay buffers 60 mM acetate buffer, pH 5.0 and 100 mM acetate buffer, pH 5.5 were used for determination of cathepsin B exopeptidase and cathepsin L activities, respectively. Each assay buffer contained 0.1% PEG 8000 (Sigma-Aldrich, St. Louis, MO, USA), 5 mM cysteine, and 1.5 mM EDTA. Enzymes were activated in the assay buffer for 5 min at 37 °C prior to the assay.

Determination of relative inhibition

Substrates Abz-Gly-Ile-Val-Arg-Ala-Lys(Dnp)-OH (Bachem) and Z-Phe-Arg-AMC (Bachem) were used to determine the effect of inhibitors on cathepsin B exopeptidase and cathepsin L activity, respectively. To initiate the reaction 90 µl of activated enzyme in the assay buffer was added to the wells of a black microplate containing 5 µl of substrate (final concentration 5 and 1 µM for cathepsin B exopeptidase activity, and 2 µM for cathepsin L) and 5 µl of inhibitor at concentration 50 µM. Formation of the fluorescent degradation products during reaction was continuously monitored at 460 nm ± 10 nm with excitation at 380 nm ± 20 nm for Z-Phe-Arg-AMC and at 420 nm ± 10 nm with excitation at 320 nm ± 20 nm for Abz-Gly-Ile-Val-Arg-Ala-Lys(Dnp)-OH at 37°C on a Tecan Infinite M1000 (Mannedorf, Switzerland) spectrofluorimeter. All assay mixtures contained 5% (v/v) DMSO. To all assay mixtures also 0.01% Triton X-100 was added, to prevent false-positive inhibition due to the formation of compound aggregates [9]. All measurements were performed in triplicates and repeated twice. The relative inhibition was calculated using the equation: *Relative inhibition (%) = 100(1-* v_i/v_o*)*, where v_i and v_o designate the reaction velocities in the presence and absence of inhibitor, respectively.

Determination of IC_{50} *values*

IC_{50} values for inhibition of cathepsin B exopeptidase activity and cathepsin L activity were determined by measuring the reaction velocities at constant substrate concentration in the presence of increasing concentrations of the inhibitor. The assay was performed as described above for determination of relative inhibition whereby nine different final concentrations of inhibitors were used 0, 1, 5, 10, 25, 50, 100, 250 and 500 µM. Prior the assay the inhibitors were diluted in DMSO in a way to ensure all assay mixtures contained equal 5% amount of DMSO. All measurements were performed in duplicates and repeated twice. IC_{50} values were calculated from relative inhibition of enzyme activities using the GraphPad Prism 6.0 software package (GraphPad Software Inc., La Jolla, San Jose, CA, USA). Data are presented as mean ± SEM.

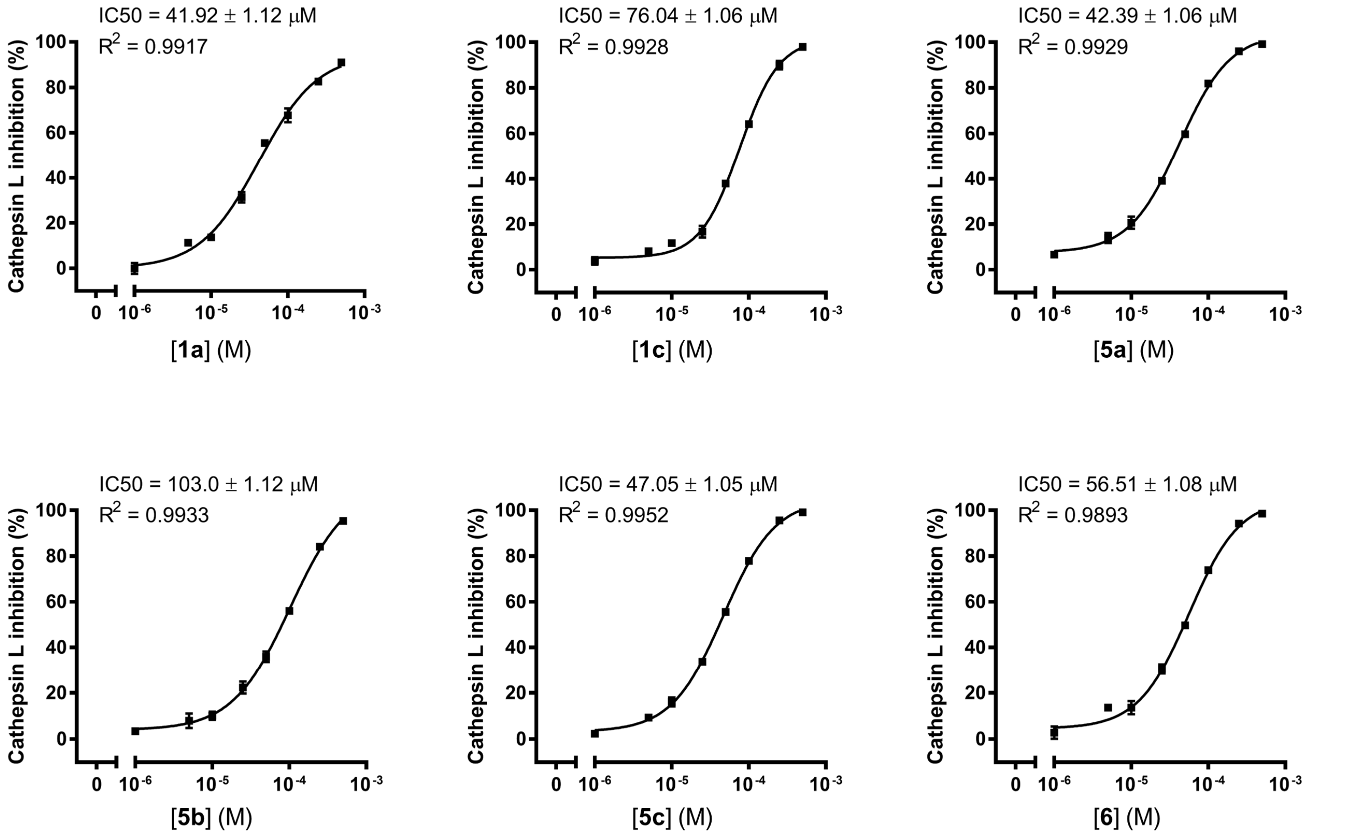

Figure S17. Dose-response curves for the inhibition of cathepsin L activity of glycopeptide conjugates and apocarotenoids. Data are presented as mean ± SEM from *n* = 3 biologically independent samples.

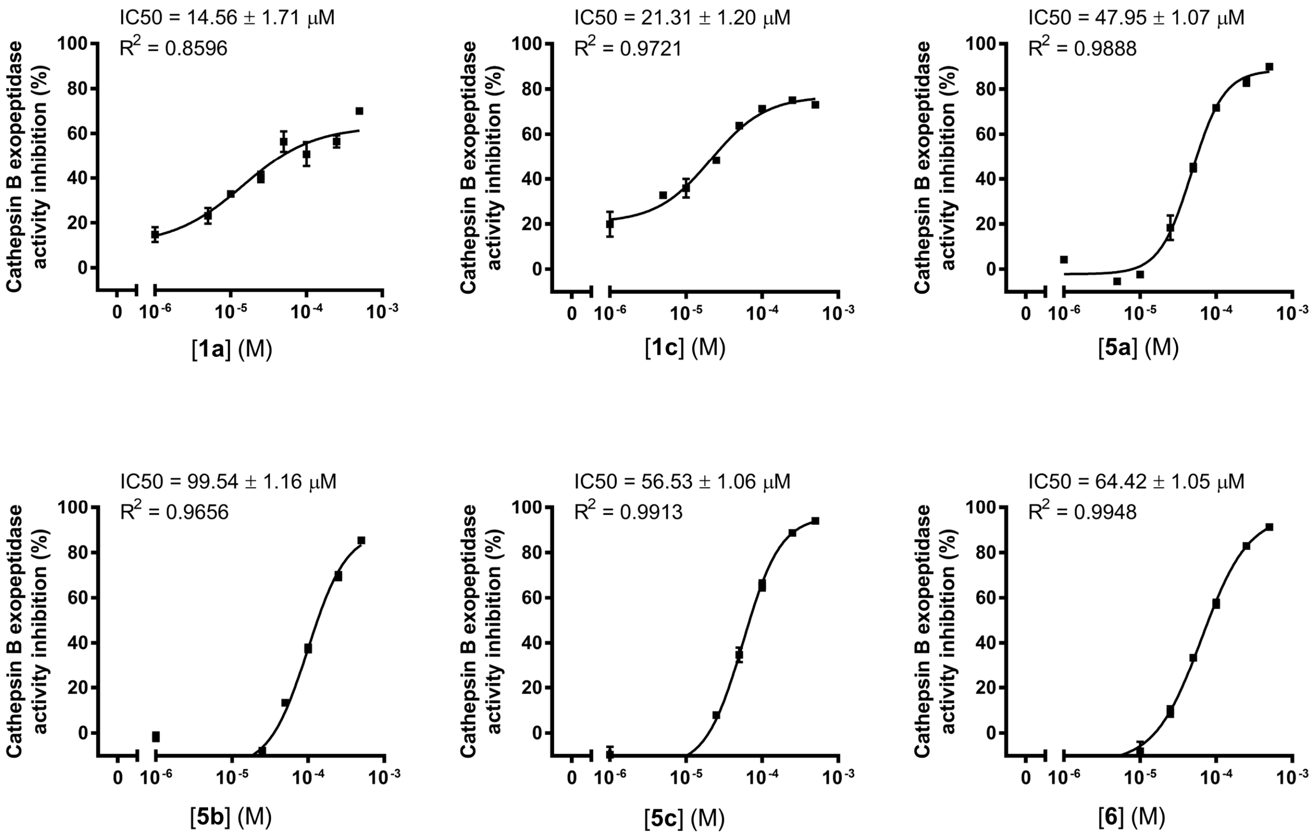

Figure S18. Dose-response curves for the inhibition of cathepsin B exopeptidase activity of glycopeptide conjugates and apocarotenoids. Data are presented as mean ± SEM from $n = 3$ biologically independent samples.

3CLPro inhibition assay

For the fluorescence measurements a generic 3CLPro FRET peptide substrate was used (Hilyte^{TM} Fluor - 488 - ESATLQSGLRKAK - (QXL® - 520) - NH2, Anaspec, Inc., Cat. no. 510/791-9560) [10]. The conditions of the measurements were as follows: either 600 nM or no 3CLPro, 250 nM substrate, 5% DMSO and the requisite concentration of inhibitors in 3CLPro reaction buffer at a final volume of 50 μl/well. The covalent 3CLPro inhibitor 5-Chloropyridin-3-yl-benzo-(b)-thiophene-2-carboxylate (Maybridge, Ltd., Cat. no. BTB07408SC) was used in the assays in 10 μM final concentration as a control [11]. Fluorescence was measured on a black 384-well plate (Thermo Fisher Scientific Inc., Cat. no.

95040020) with a fluorescence microplate reader (Victor[2] 1420 multilabel counter, PerkinElmer Inc.) at 485/520 nm for excitation and emission wavelength respectively, in duplicates.

The candidates were first screened in 100 μM final concentrations. Those showing more than 50% inhibition were tested at a two-fold dilution series during a dose-response measurement, consisting of 6-8 data points. IC_{50} values were determined using a logistic curve fit (Origin 8, Northampton, MA).

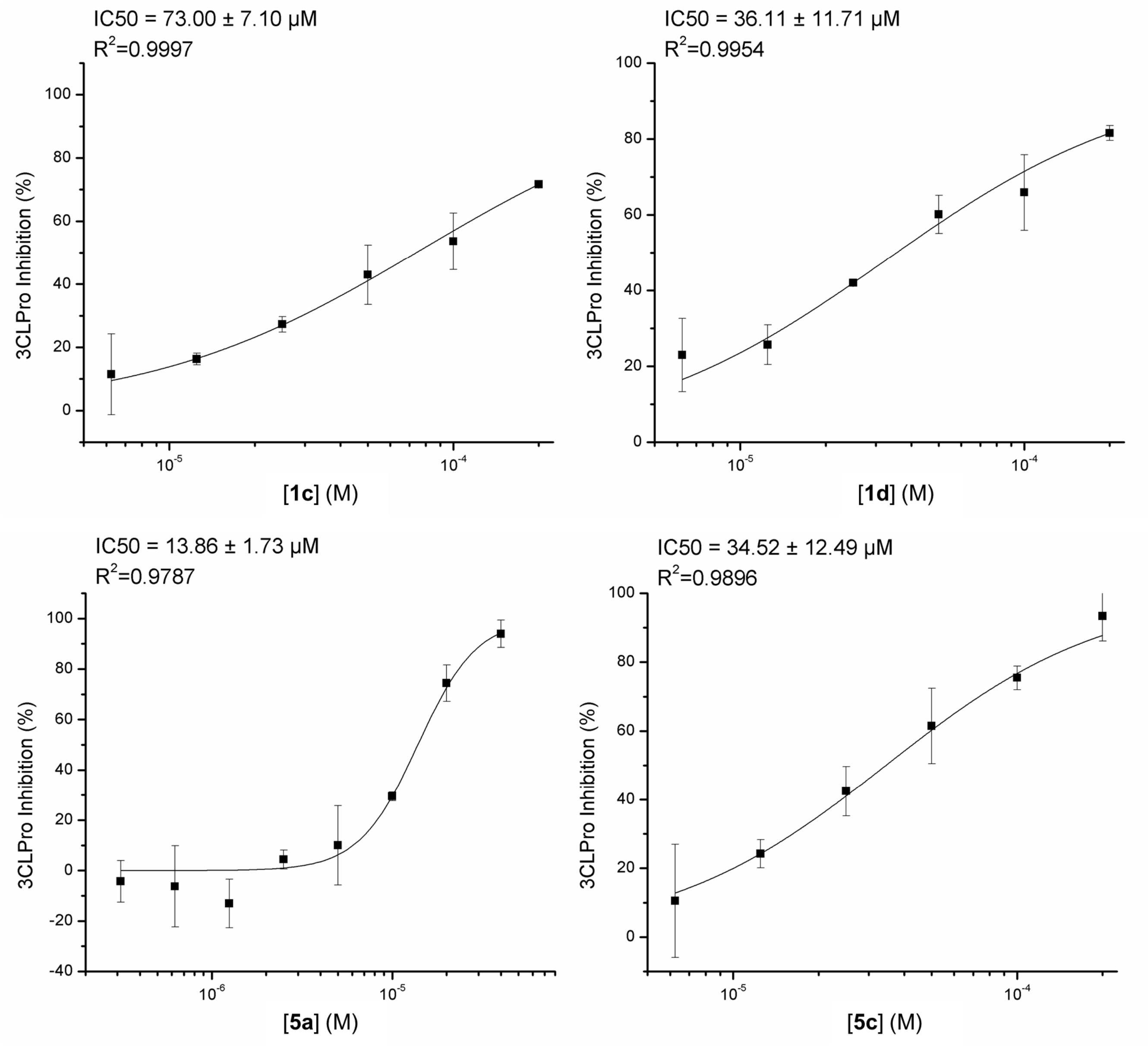

Figure S19. Dose-response curves for the inhibition of 3CLPro activity of glycopeptide conjugates and apocarotenoids. Data are presented as mean ± SEM from *n* = 2 biologically independent samples.

Computational modeling

Compounds were drawn and prepared with Marvin (version 21.4, 2021, ChemAxon, http://www.chemaxon.com), where preparation entailed identifying the major microspecies (protomer/tautomer) at a pH of 7.4, adding explicit hydrogen atoms and relaxing the 3D structure into a local minimum. Protein structures (CatB: 6AY2 [12], CatL: 3H8B [13], 3CLPro: 6LU7 [14]) were prepared with the Protein Preparation Wizard of the Schrödinger software suite (hydrogens were added, bond orders were assigned, missing sidechains and loops were filled in, H-bond networks were optimized and a restrained minimization was performed) [15]. Initial structures for low-mode docking were produced by placing the ligands in the respective binding sites by Glide SP docking [16,17]. The structures were prepared for the Amber package with Antechamber [18] and Parmed [19], and LMOD simulations were performed with Amber 18 [20]. We have performed 10 LMOD iterations after defining the ligand and the flexible loops in the vicinity of the binding sites as flexible, and kept low-energy conformations within an energy window of 50 kcal/mol as the output of the calculations. In every iteration, 5 low-frequency vibrational modes (out of the 15 lowest-frequency modes) are randomly selected and explored in both directions to look for new energy minima. In addition to the LMOD moves, 20 explicit translations and rotations are applied to the ligand, which may or may not lower the energy further. As the output of the calculations, maximum 50 conformations are kept, with the following constraints: (i) each conformation is max. 50kcal/mol above the lowest-energy conformation, and (ii) each conformation is unique (min. 1 Å RMSD from each of the other conformations, calculated for the flexible atoms). The list of binding site residues defined as flexible are as follows (sequential residue numbers starting from 1):

CatB: 22-29, 66-77, 110-122, 176, 181, 196, 199, 221

CatL: 17-25, 63-69, 137-140, 160-162, 187-197

3CLPro: 23-27, 41-51, 139-145, 165-171, 186-192

References

[1] Scharinger, F.; Pálvölgyi, A. M.; Zeindlhofer, V.; Schnürch, M.; Schröder, C.; Bica-Schröder K. Counterion Enhanced Organocatalysis: A Novel Approach for the Asymmetric Transfer Hydrogenation of Enone. *ChemCatChem* **2020**, *12* (14), 3776–3782. DOI: 10.1002/cctc.202000414

[2] Sztaricskai, F.; Batta, G.; Herczegh, P.; Balázs, A.; Jekő, J.; Rőth, E.; Szabó, P. T.; Kardos, S.; Rozgonyi, F.; Boda, Z. A new series of glycopeptide antibiotics incorporating a squaric acid moiety. Synthesis, structural and antibacterial studies. *J Antibiot.* **2006**, *59* (9), 564–582. DOI: 10.1038/ja.2006.77

[3] Malabarba, A.; Ferrari, P.; Gallo, G. G.; Kettenring, J.; Cavalleri, B. Teicoplanin, antibiotics from Actinoplanes teichomyceticus nov. sp. VII. Preparation and NMR characteristics of the aglycone of teicoplanin. *J Antibiot.* **1986**, *39* (10), 1430–1442. DOI: 10.7164/antibiotics.39.1430

[4] Häberli, A.; Pfander H. Synthesis of Bixin and Three Minor Carotenoids from Annatto (Bixa orellana). *Chim. Acta* **1999**, *82* (5), 696–706. DOI: 10.1002/(SICI)1522-2675(19990505)82:5<696::AID-HLCA696>3.0.CO;2-X

[5] Frederico, D.; Donate, P. M.; Constantino, M. G.; Bronze, E. S.; Sairre, M. I. A short and efficient synthesis of crocetin-dimethylester and crocetindial. *J. Org. Chem.* **2003**, *68* (23), 9126–9128. DOI: 10.1021/jo034545y

[6] Scotter, M. The chemistry and analysis of annatto food colouring: a review. *Food Addit. Contam. Part A* **2009**, *26*, 1123–1145. DOI: 10.1080/02652030902942873

[7] Kuhelj, R.; Dolinar, M.; Pungerčar, J.; Turk, V. The Preparation of Catalytically Active Human Cathepsin B from Its Precursor Expressed in Escherichia coli in the Form of Inclusion Bodies. *Eur. J. Biochem.* **1995**, *229* (2), 533–539. DOI: 10.1111/j.1432-1033.1995.0533k.x

[8] Dolinar, M.; Maganja, D. B.; Turk, V. Expression of Full-Length Human Procathepsin L cDNA in Escherichia coli and Refolding of the Expression Product. *Biol. Chem. Hoppe. Seyler.* **1995**, *376* (6), 385–388. DOI: 10.1515/bchm3.1995.376.6.385

[9] Feng, B. Y.; Shoichet, B. K. A detergent-based assay for the detection of promiscuous inhibitors. *Nat. Protoc.* **2006**, *1* (2), 550–553. DOI: 10.1038/nprot.2006.77

[10] Tomar, S.; Johnston, M. L.; St John, S. E.; Osswald, H. L.; Nyalapatla, P. R.; Paul, L. N.; Ghosh, A. K.; Denison, M. R.; Mesecar, A. D. Ligand-induced dimerization of Middle East Respiratory Syndrome (MERS) Coronavirus nsp5 protease (3CLpro): Implications for nsp5 regulation and the development of antivirals. *J. Biol. Chem.* **2015**, *290* (32), 19403–19422. DOI: 10.1074/jbc.M115.651463

[11] Zhang, J.; Pettersson, H. I.; Huitema, C.; Niu, C.; Yin, J.; James, N. M. G.; Eltis, L. D.; Vederas, J. C. Design, synthesis, and evaluation of inhibitors for severe acute respiratory syndrome 3C-like protease

based on phthalhydrazide ketones or heteroaromatic esters. *J. Med. Chem.* **2007**, *50* (8), 1850–1864. DOI: 10.1021/jm061425k

[12] Wei, B.; Gunzner-Toste, J.; Yao, H.; Wang, T.; Wang, J.; Xu, Z.; Chen, J.; Wai, J.; Nonomiya, J.; Tsai, S. P.; Chuh, J.; Kozak, K. R.; Liu, Y.; Yu, S.-F.; Lau, J.; Li, G.; Phillips, G. D.; Leipold, D.; Kamath, A.; Su, D.; Xu, K.; Eigenbrot, C.; Steinbacher, S.; Ohri, R.; Raab, H.; Staben, L. R.; Zhao, G.; Flygare, J. A.; Pillow, T. H.; Verma, V.; Masterson, L. A.; Howard, P. W.; Safina, B. Discovery of Peptidomimetic Antibody-Drug Conjugate Linkers with Enhanced Protease Specificity. *J. Med. Chem.* **2018**, *61* (3), 989–1000. DOI: 10.1021/acs.jmedchem.7b01430

[13] Shenoy, R. T.; Chowdhury, S. F.; Kumar, S.; Joseph, L.; Purisima, E. O.; Sivaraman, J. A combined crystallographic and molecular dynamics study of cathepsin L retrobinding inhibitors. *J. Med. Chem.* **2009**, *52* (20), 6335–6346. DOI: 10.1021/jm900596y

[14] Jin, Z.; Du, X.; Xu, Y.; Deng, Y.; Liu, M.; Zhao, Y.; Zhang, B.; Li, X.; Zhang, L.; Peng, C.; Duan, Y.; Yu, J.; Wang, L.; Yang, K.; Liu, F.; Jiang, R.; Yang, X.; You, T.; Liu, X.; Yang, X.; Bai, F.; Liu, H.; Liu, X.; Guddat, L. W.; Xu, W.; Xiao, G.; Qin, C.; Shi, Z.; Jiang, H.; Rao, Z.; Yang, H. Structure of M^{pro} from SARS-CoV-2 and discovery of its inhibitors. *Nature* **2020**, *582*, 289–293. DOI: 10.1038/s41586-020-2223-y

[15] Sastry, G. M.; Adzhigirey, M.; Day, T.; Annabhimoju, R.; Sherman, W. Protein and ligand preparation: parameters, protocols, and influence on virtual screening enrichments. *J. Comput. Aided. Mol. Des.* **2013**, *27* (3), 221–34. DOI: 10.1007/s10822-013-9644-8

[16] Friesner, R. A.; Banks, J. L.; Murphy, R. B.; Halgren, T. A.; Klicic, J. J.; Mainz, D. T.; Repasky, M. P.; Knoll, E. H.; Shelley, M.; Perry, J. K.; Shaw, D. E.; Francis, P.; Shenkin P. S.; Glide: a new approach for rapid, accurate docking and scoring. 1. Method and assessment of docking accuracy. *J. Med. Chem.* **2004**, *47* (7), 1739–1749. DOI: 10.1021/jm0306430

[17] Halgren, T. A.; Murphy, R. B.; Friesner, R. A.; Beard, H. S.; Frye, L. L.; Pollard, W. T.; Banks J. L. Glide: a new approach for rapid, accurate docking and scoring. 2. Enrichment factors in database screening. *J. Med. Chem.* **2004**, *47* (7), 1750–1759. DOI: 10.1021/jm030644s

[18] Wang, J.; Wang, W.; Kollman, P. A.; Case, D. A. Automatic atom type and bond type perception in molecular mechanical calculations. *J. Mol. Graph. Model.* **2006**, *5* (2), 247–260. DOI: 10.1016/j.jmgm.2005.12.005

[19] Shirts, M. R.; Klein, C.; Swails, J. M.; Yin, J.; Gilson, M. K.; Mobley, D. L.; Case, D. A.; Zhong E. D. Lessons learned from comparing molecular dynamics engines on the SAMPL5 dataset. *J. Comput. Aided. Mol. Des.* **2017**, *31* (1), 147–161. DOI: 10.1007/s10822-016-9977-1

[20] Salomon-Ferrer, R.; Case, D. A.; Walker, R. C. An overview of the Amber biomolecular simulation package. *Wiley Interdiscip. Rev. Comput. Mol. Sci.* **2013**, *3* (2), 198–210. DOI: 10.1002/wcms.1121

NMR spectra

NMR spectra of crocetin monomethyl ester (1b)

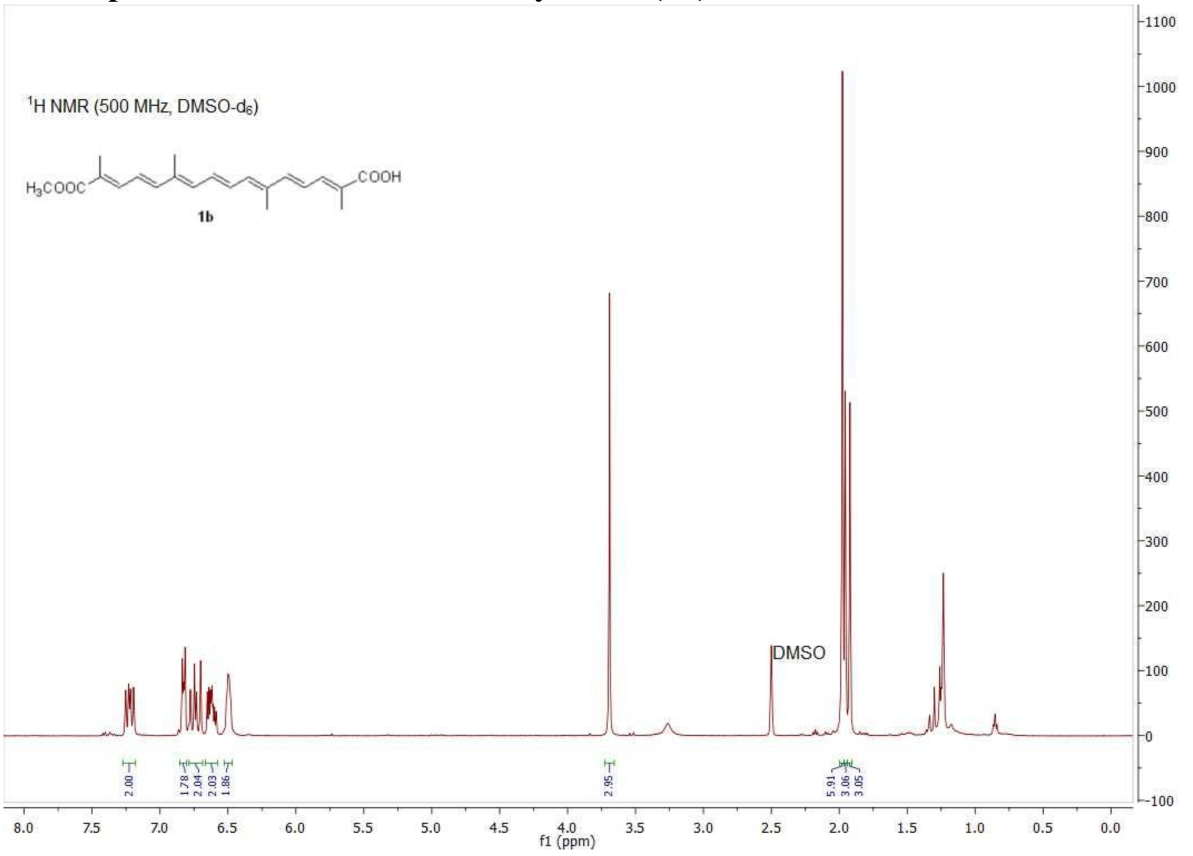

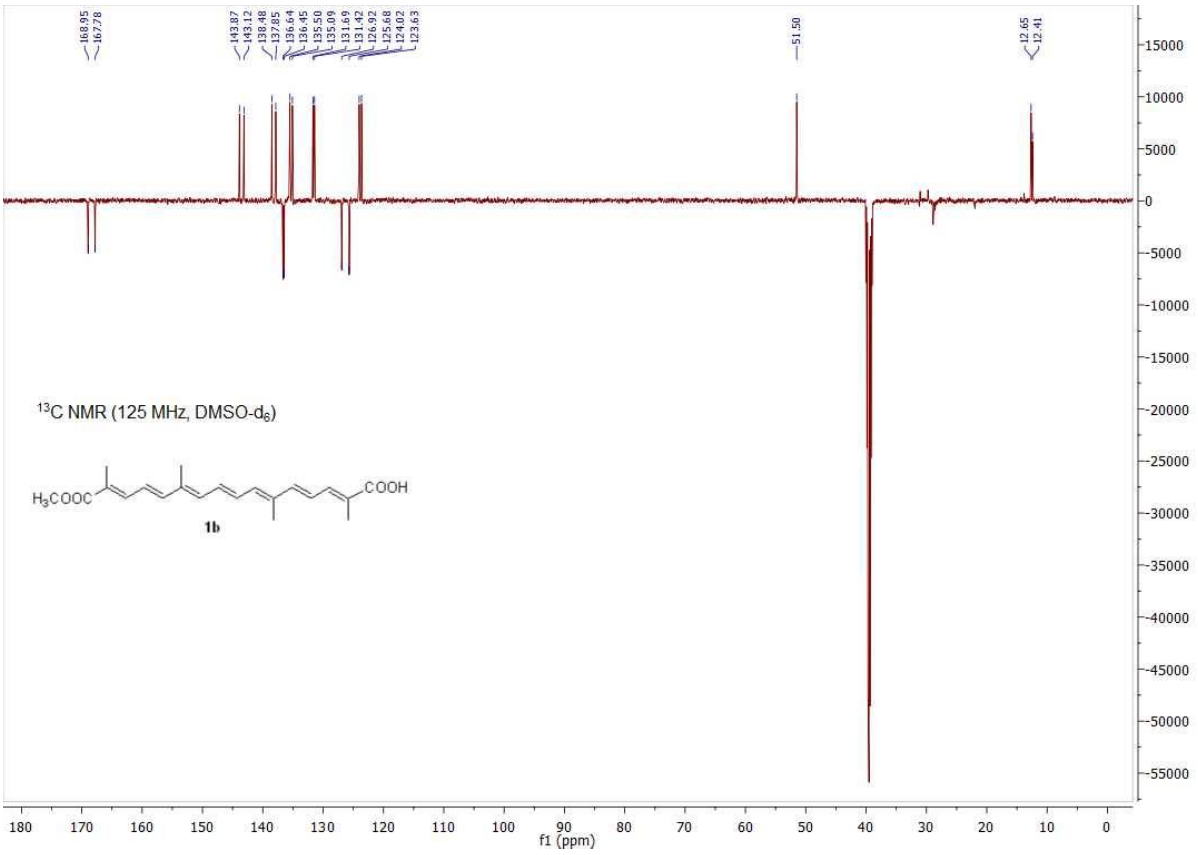

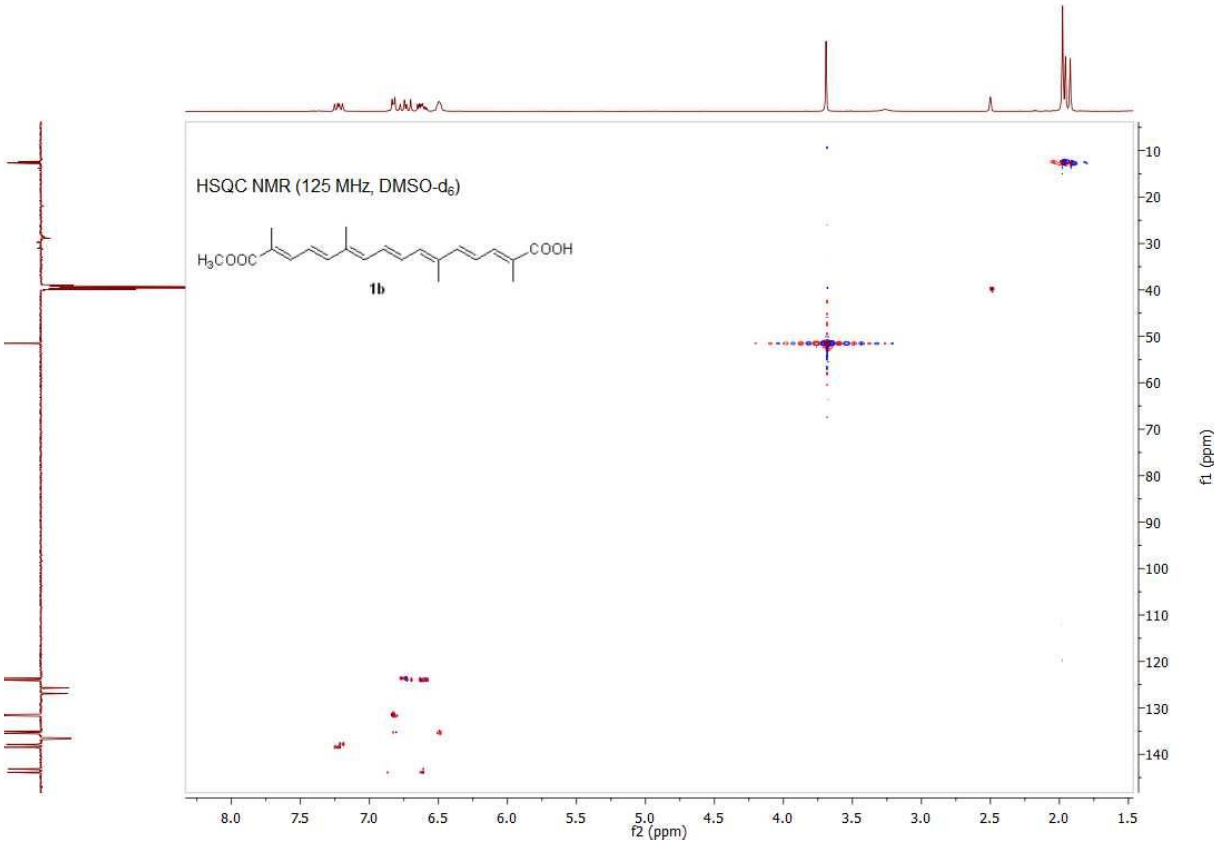

NMR spectra of β-apo-8’-carotenoic acid (1c)

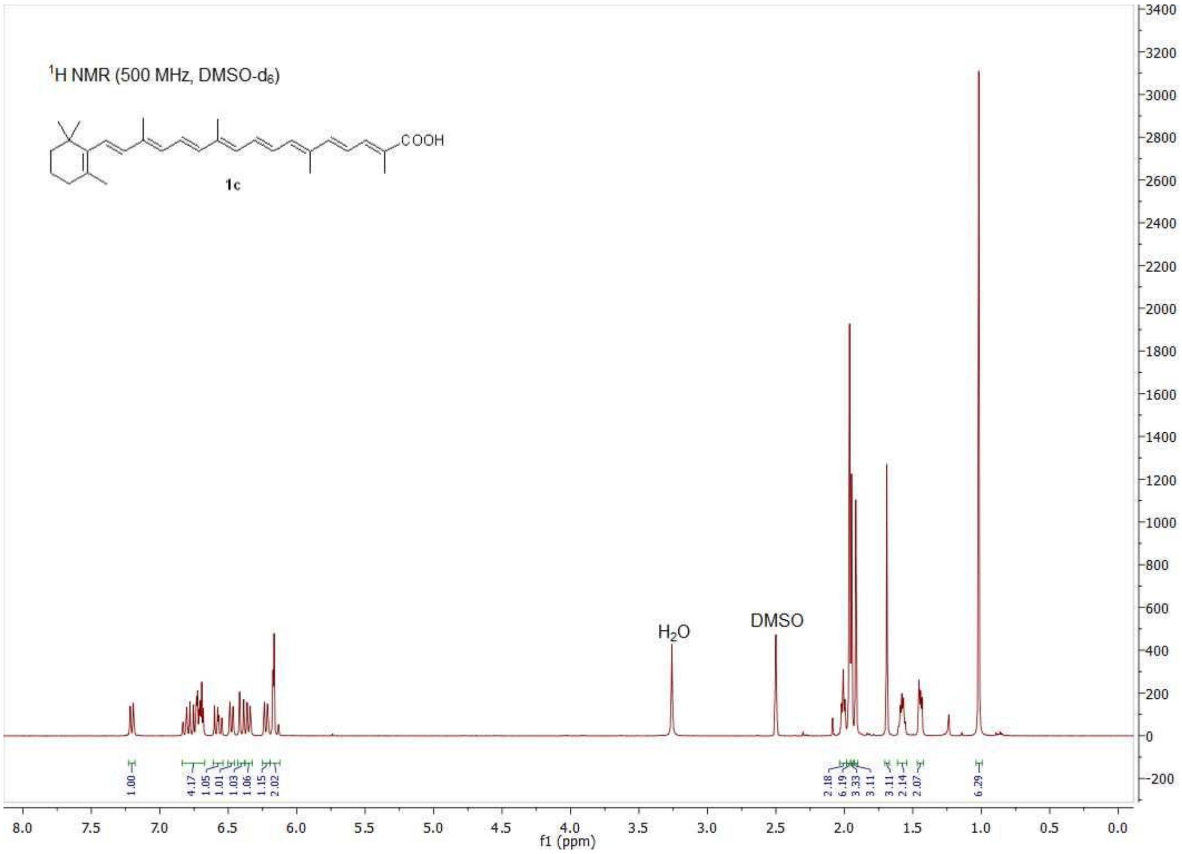

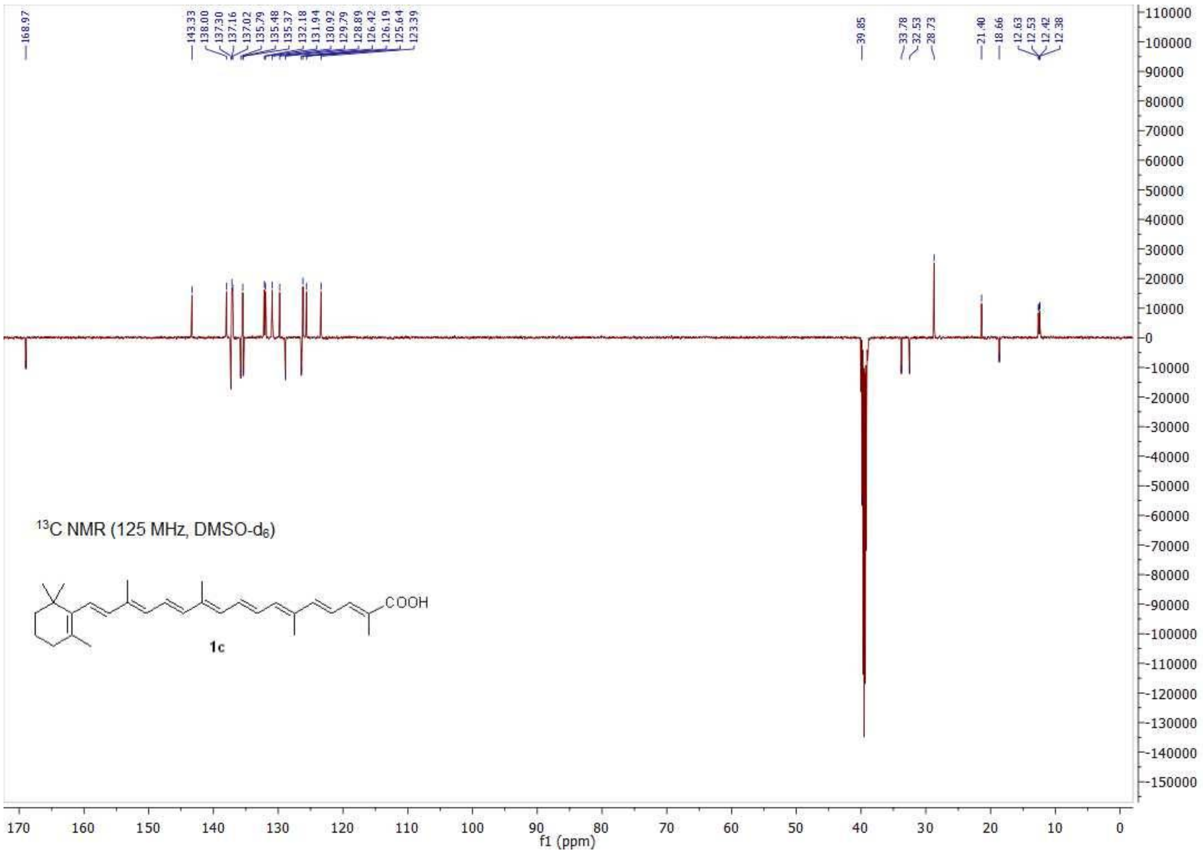
168.97
143.33
138.00
137.30
137.16
137.02
135.79
135.48
135.37
132.18
131.94
130.92
129.79
128.89
126.42
126.19
125.64
123.39
39.85
33.78
32.53
28.73
21.40
18.66
12.63
12.53
12.42
12.38
^{13}C NMR (125 MHz, DMSO-d$_6$)
COOH
1c
f1 (ppm)

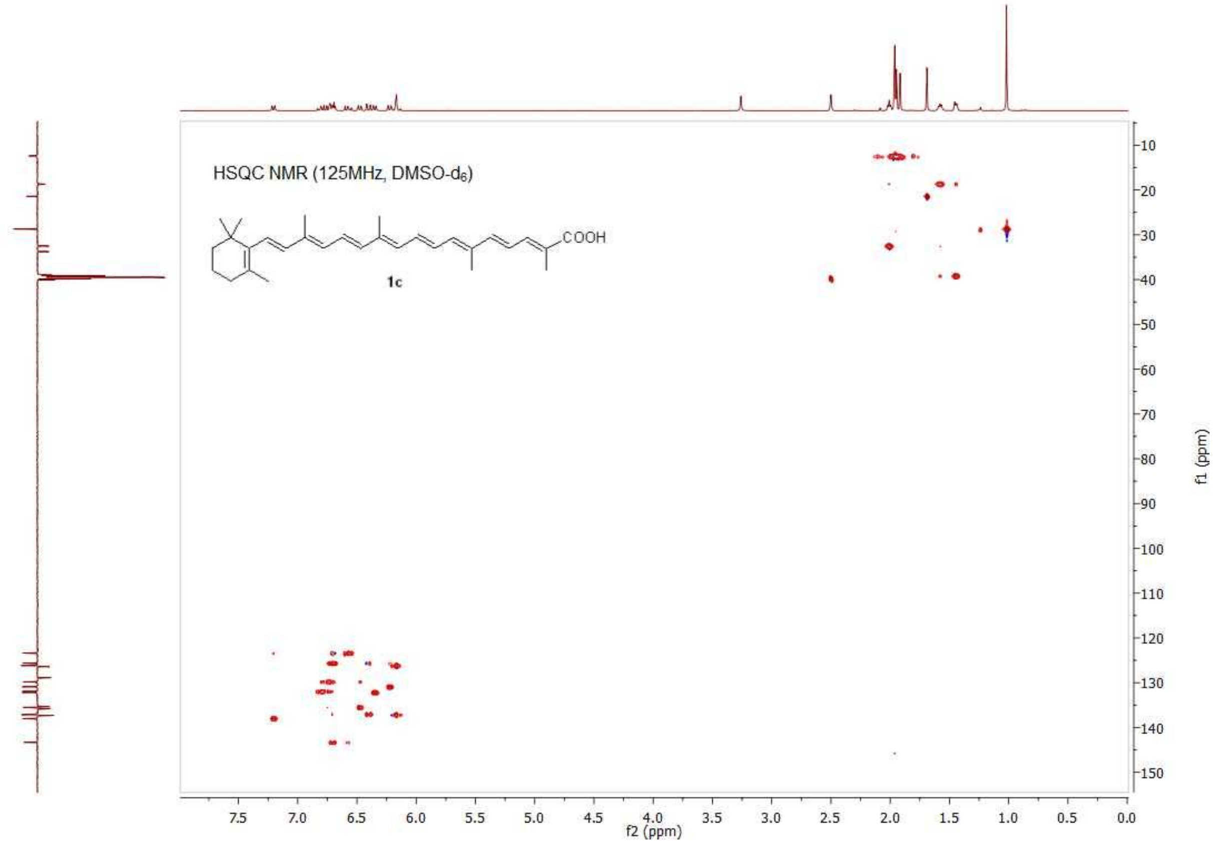
HSQC NMR (125MHz, DMSO-d$_6$)
COOH
1c
f2 (ppm)
f1 (ppm)

NMR spectra of 1c-ester

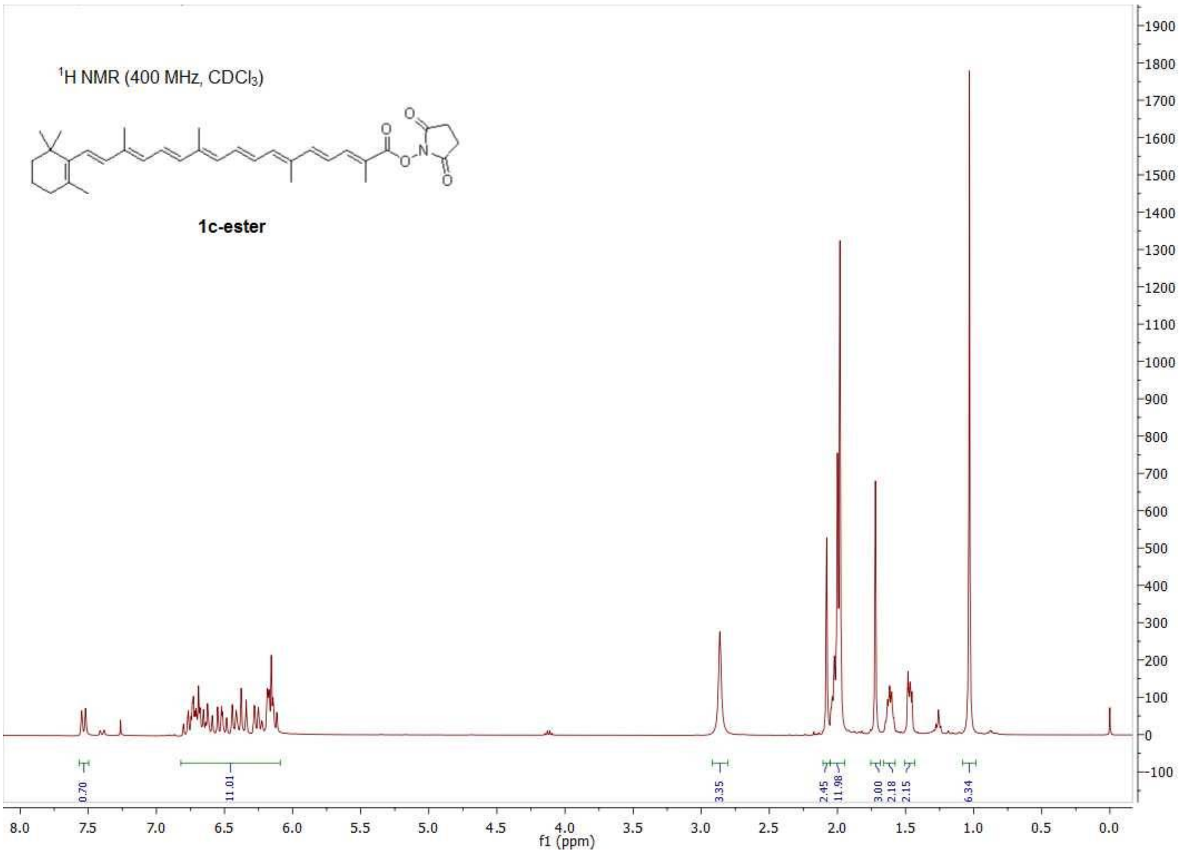

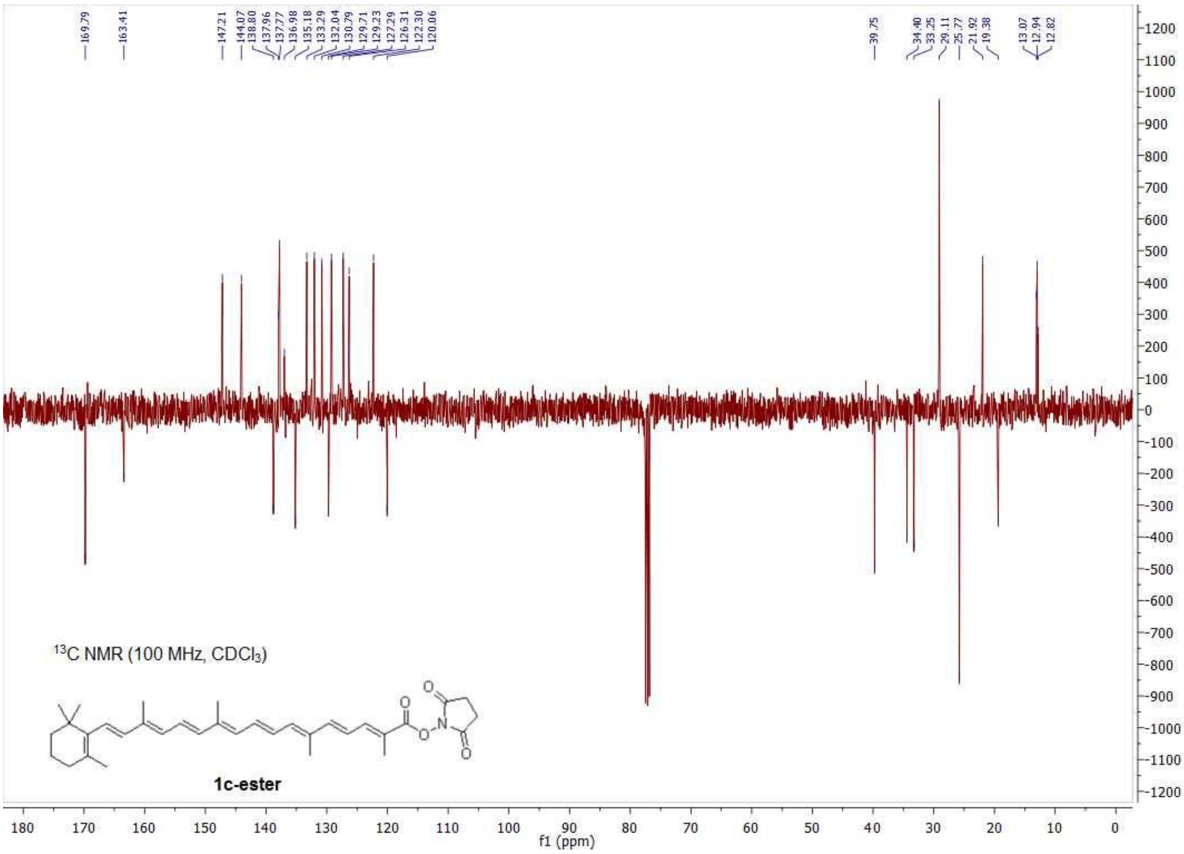

NMR spectra of 9

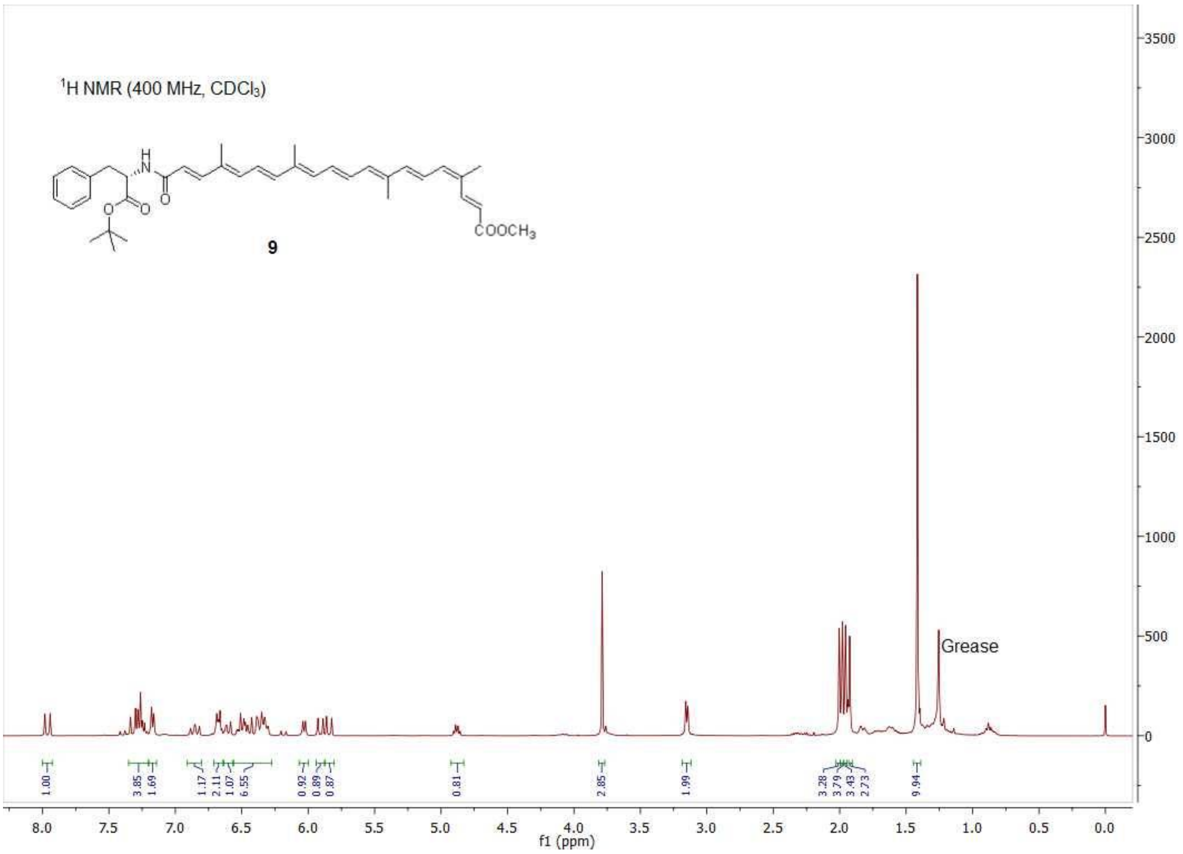

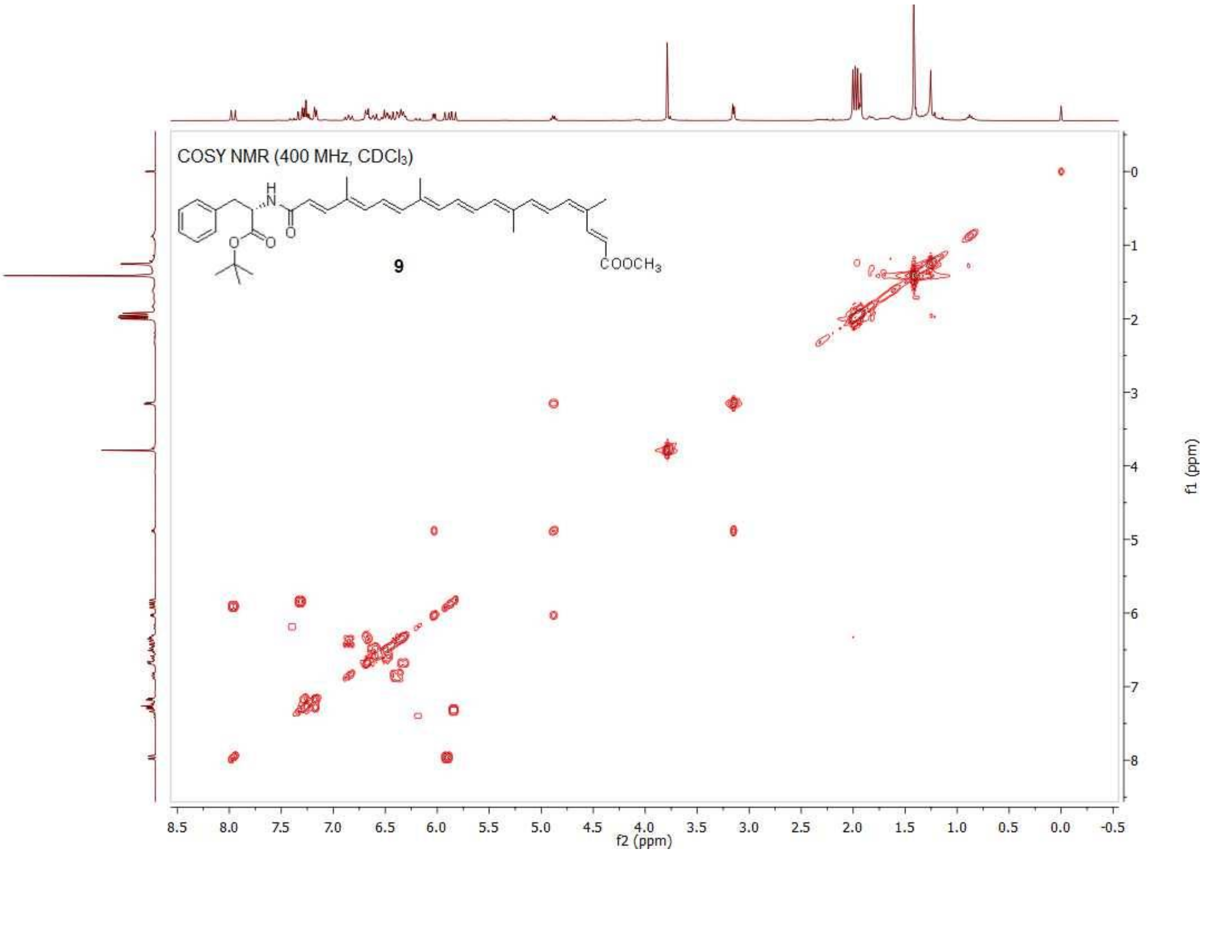

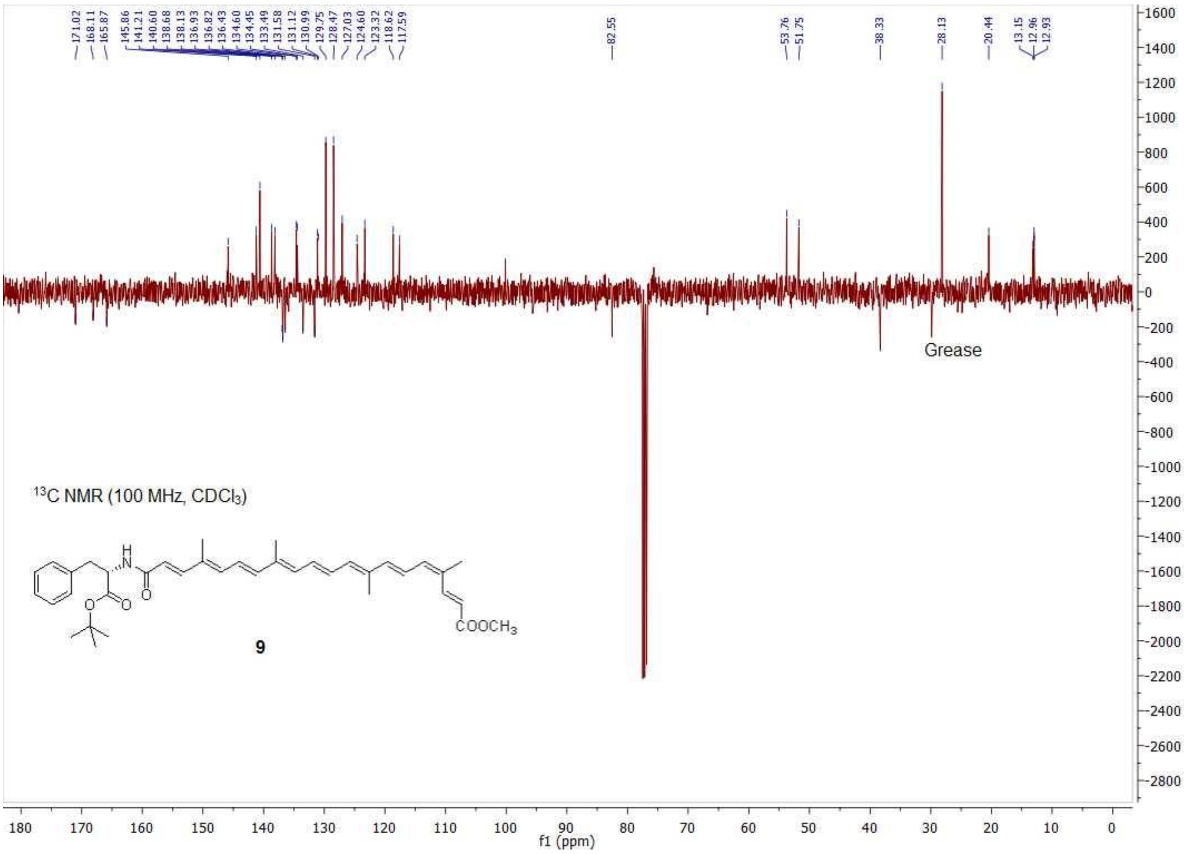

171.02
168.11
165.87
145.86
141.21
140.60
138.68
138.13
136.93
136.82
136.43
134.60
134.45
133.49
131.58
131.12
130.99
129.75
128.47
127.03
124.60
123.32
118.62
117.59
82.55
53.76
51.75
38.33
28.13
20.44
13.15
12.96
12.93
Grease
^{13}C NMR (100 MHz, CDCl$_3$)
9
COOCH$_3$
f1 (ppm)

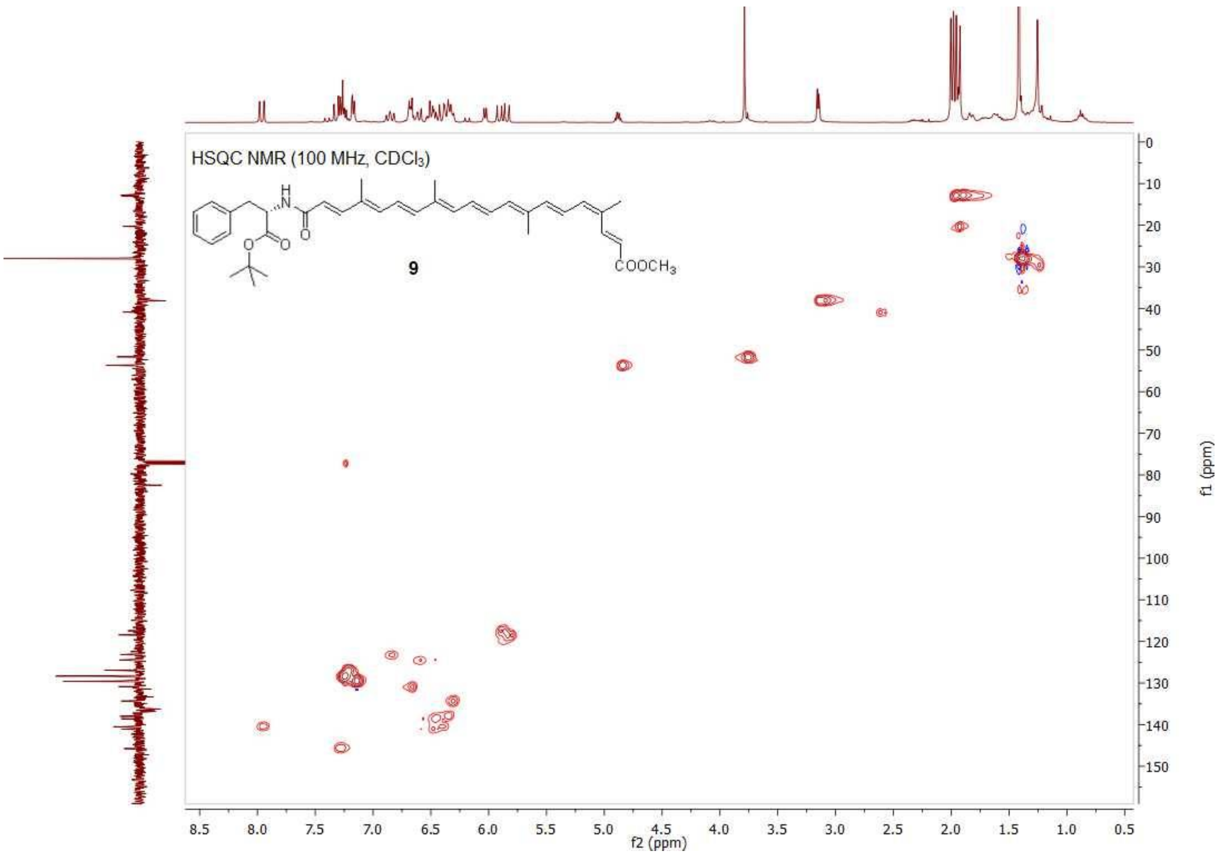

HSQC NMR (100 MHz, CDCl$_3$)
9
COOCH$_3$
f2 (ppm)
f1 (ppm)

NMR spectra of glycopeptide derivatives 5a, 5b, 5c and 7 (125 MHz and 500 MHz in DMSO)

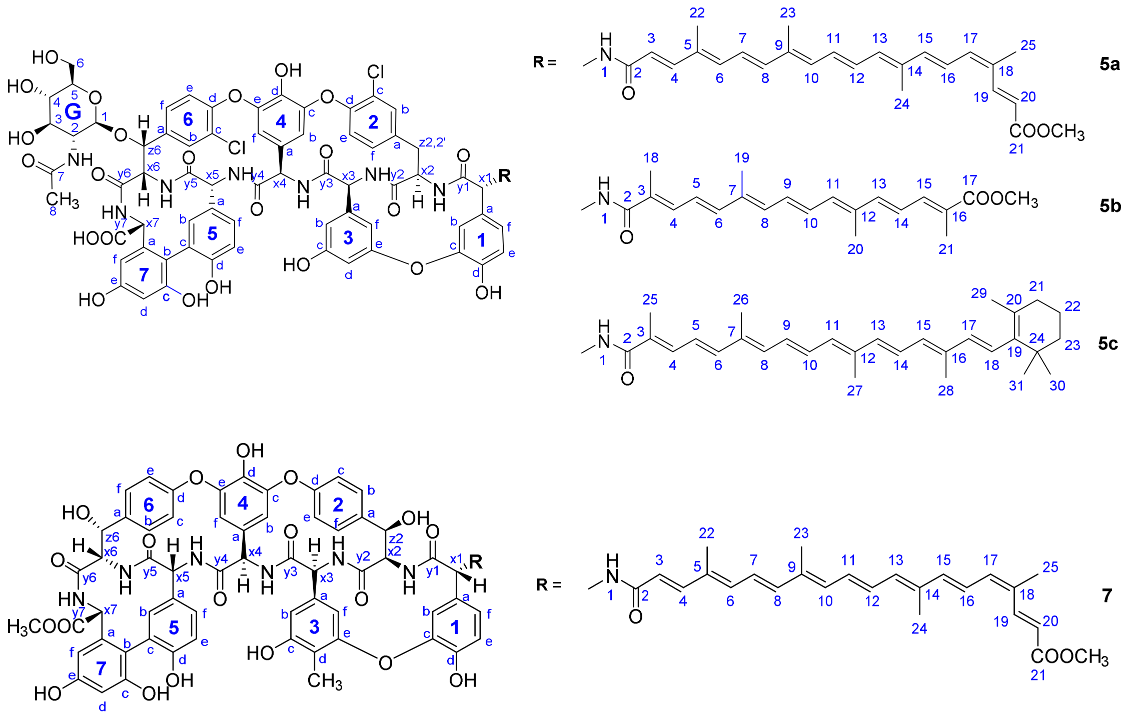

Figure S20. Atom numberings of teicoplanin pseudoaglycon derivatives (**5a-c**) and ristocetin aglycon derivative (**7**) for NMR assignment.

HSQC spectra of 5a

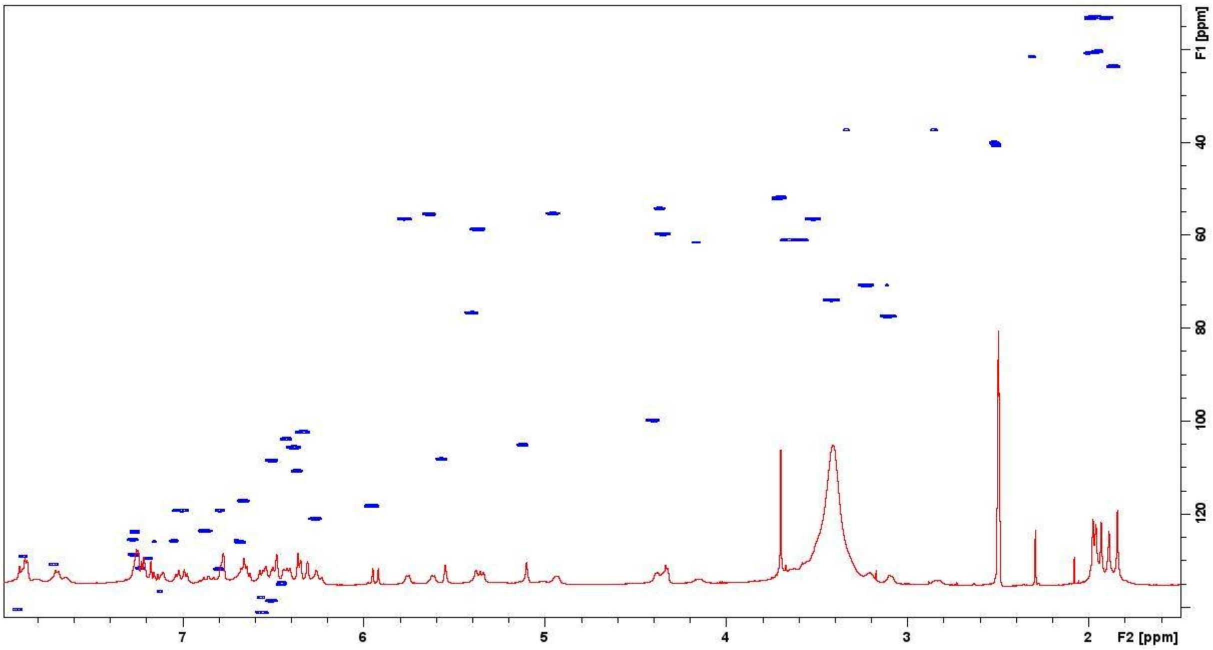

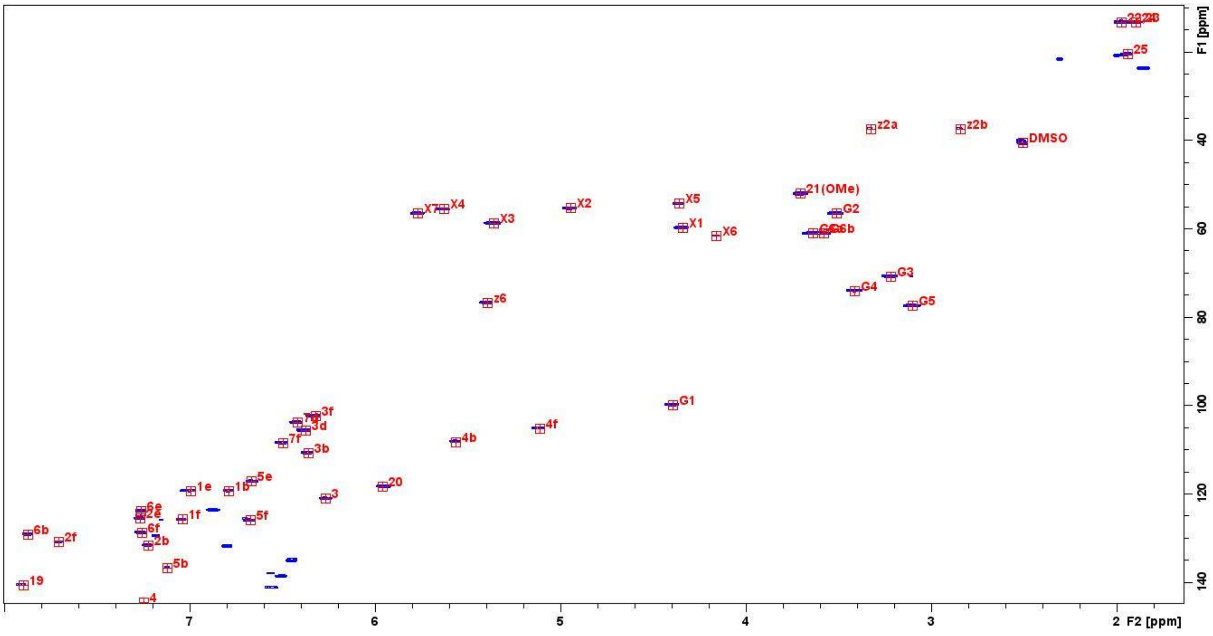

^{13}C spectrum of 5a

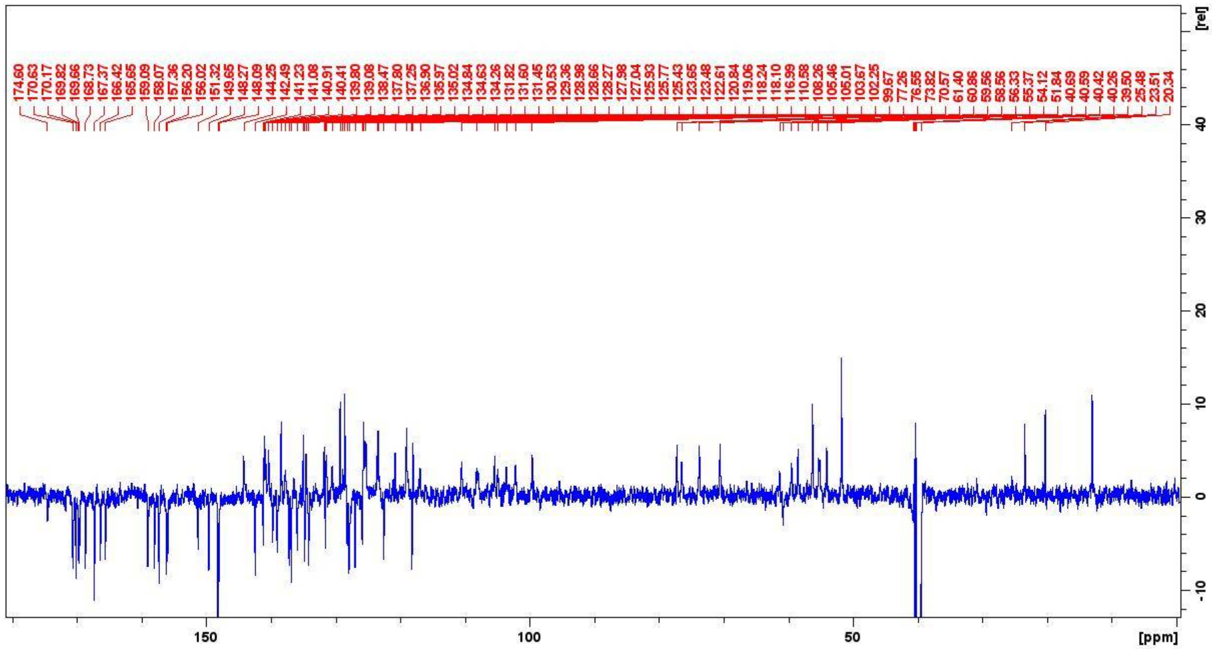

HSQC spectra of 5b

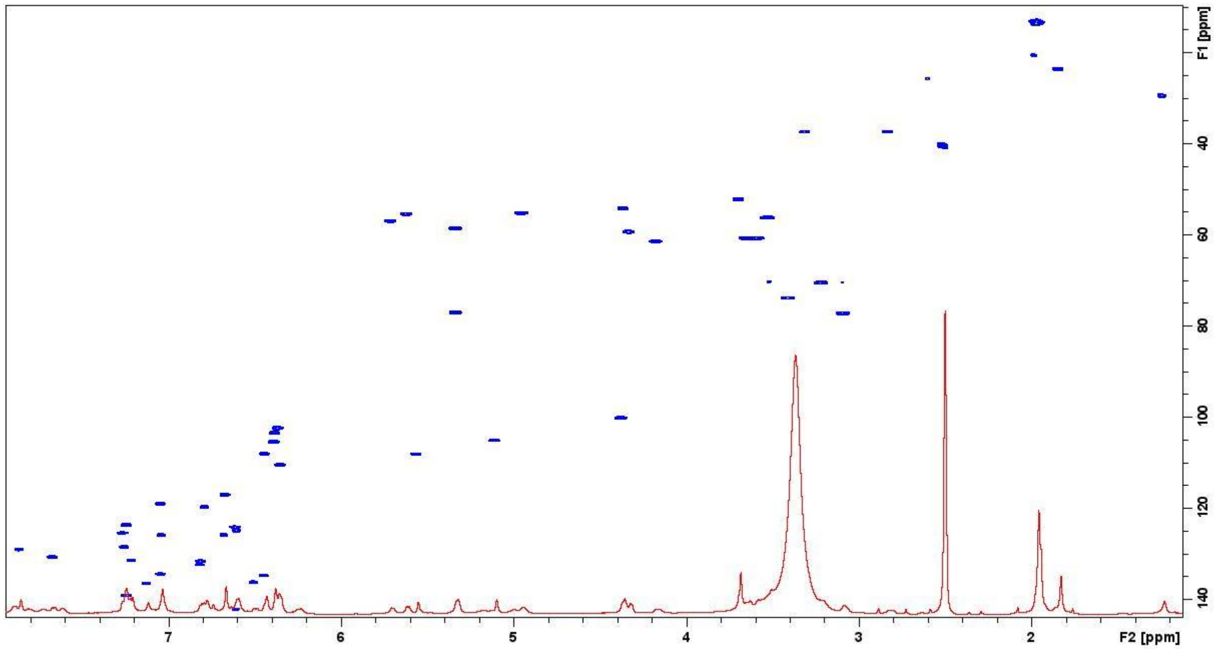

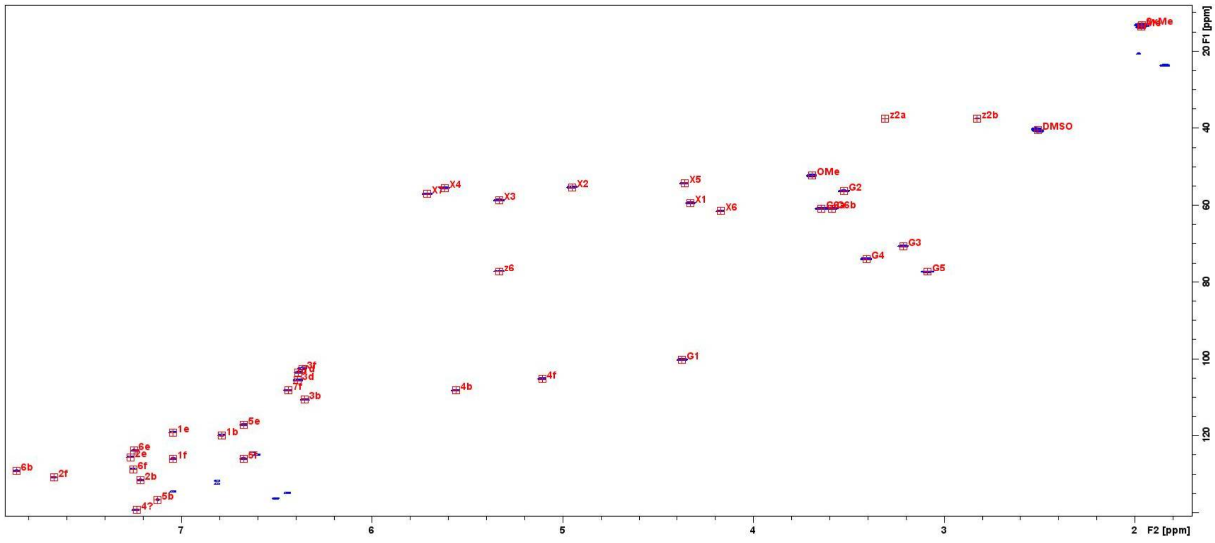

^{13}C spectrum of 5b

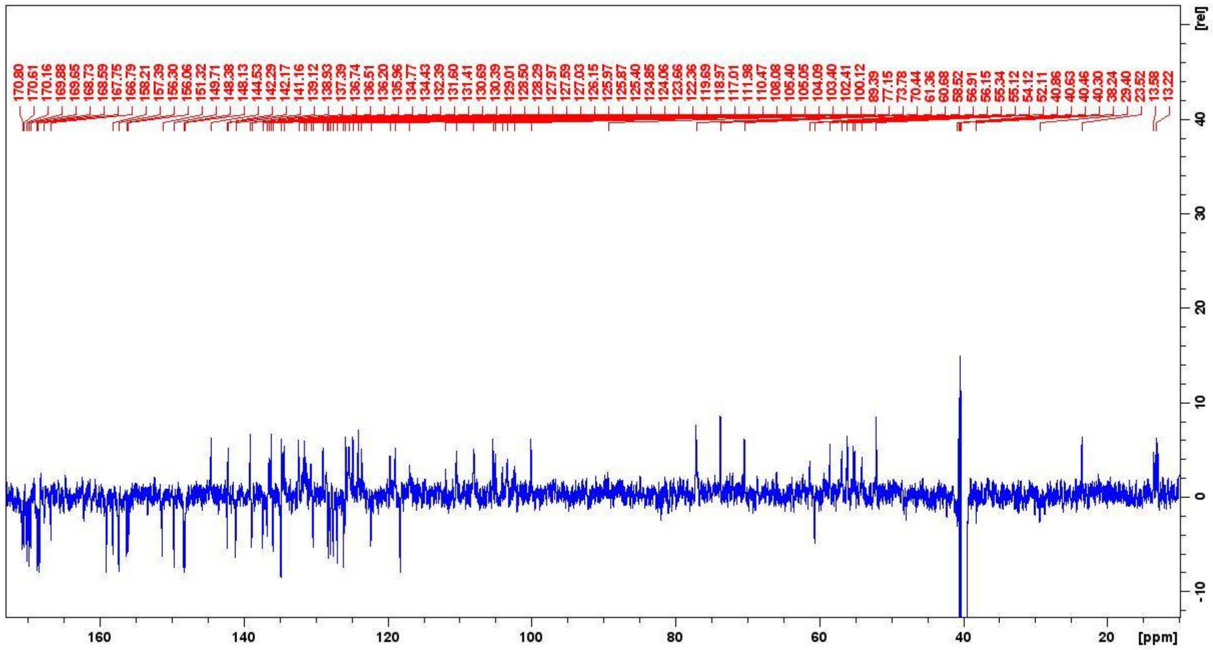

HSQC spectra of 5c

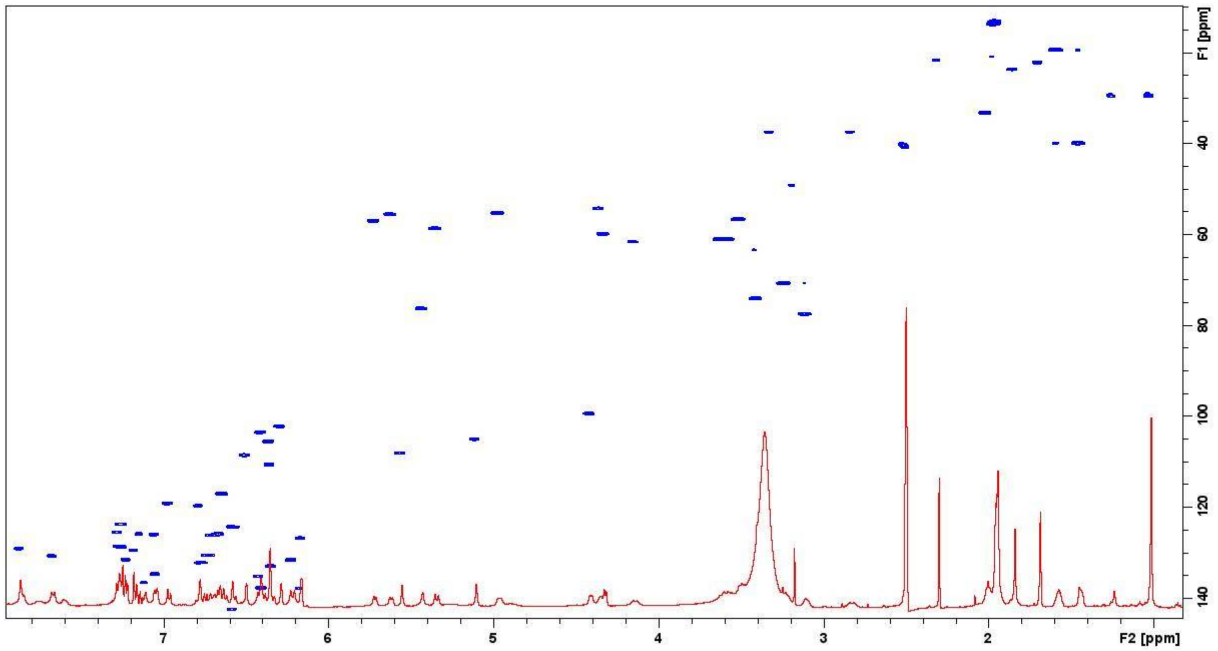

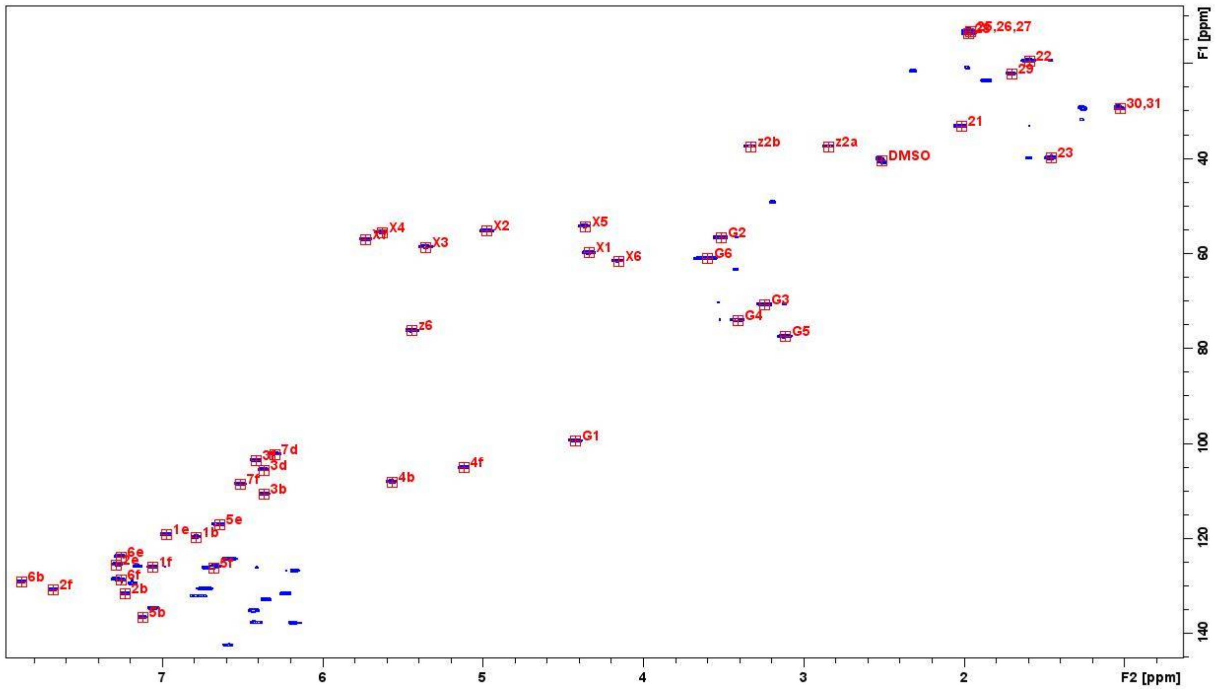

^{13}C spectrum of 5c

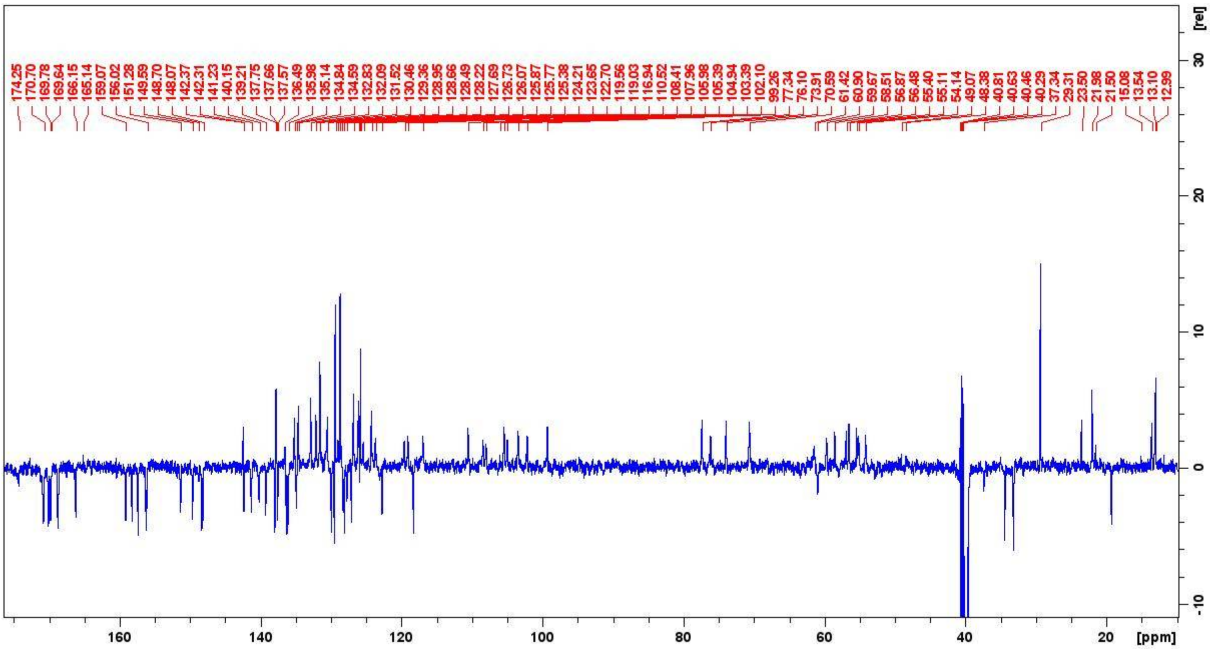

HSQC spectra of 7

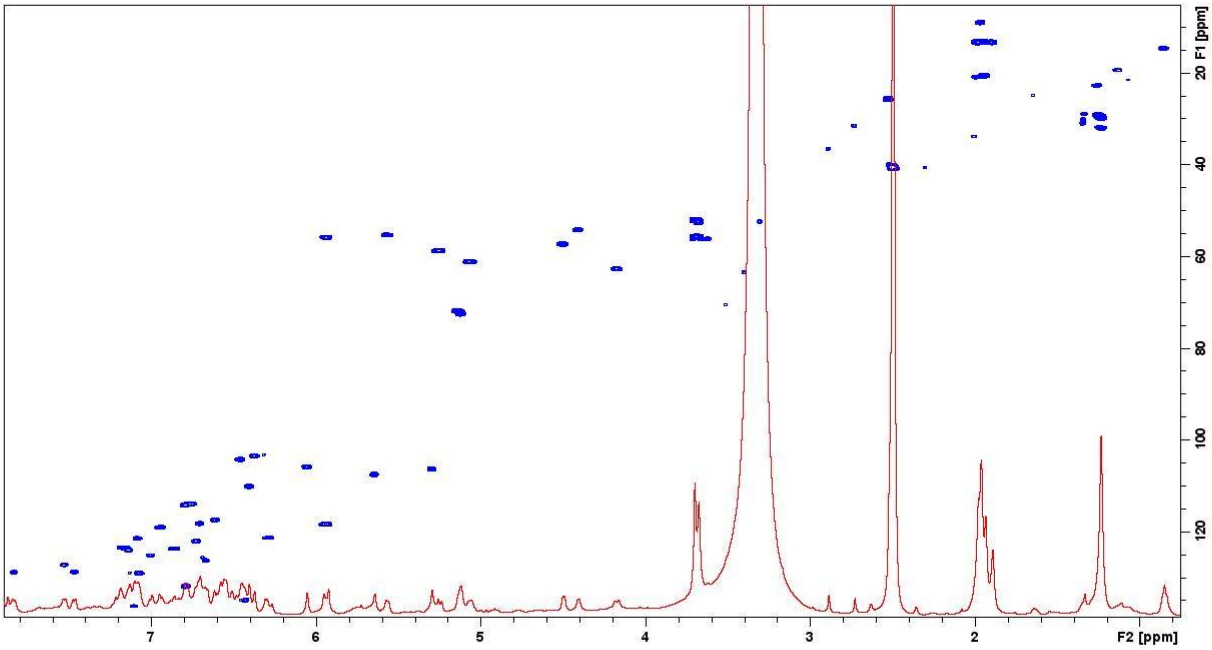

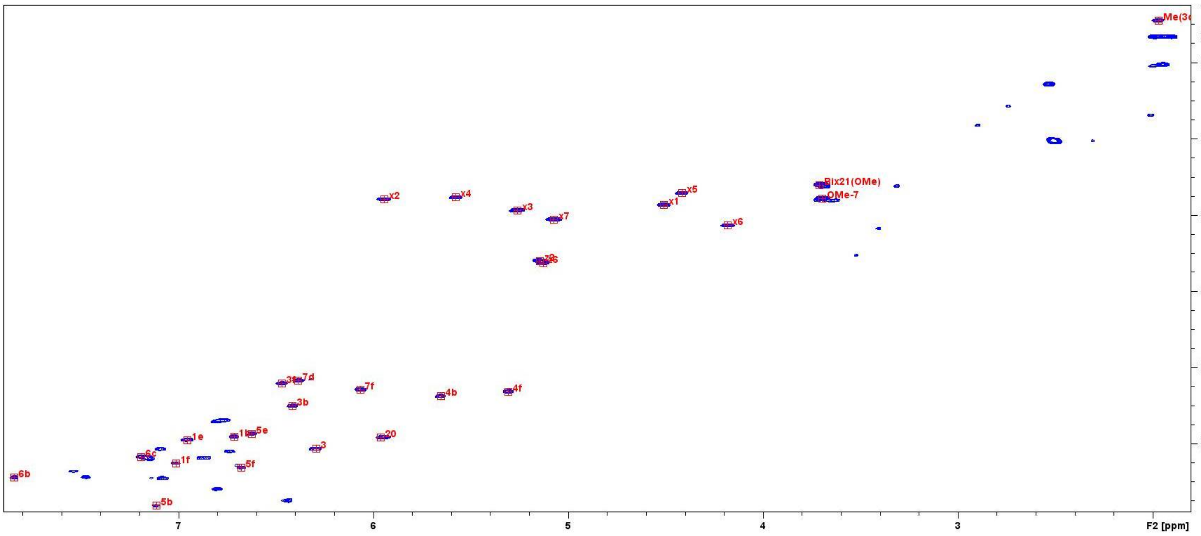

^{13}C spectrum of 7

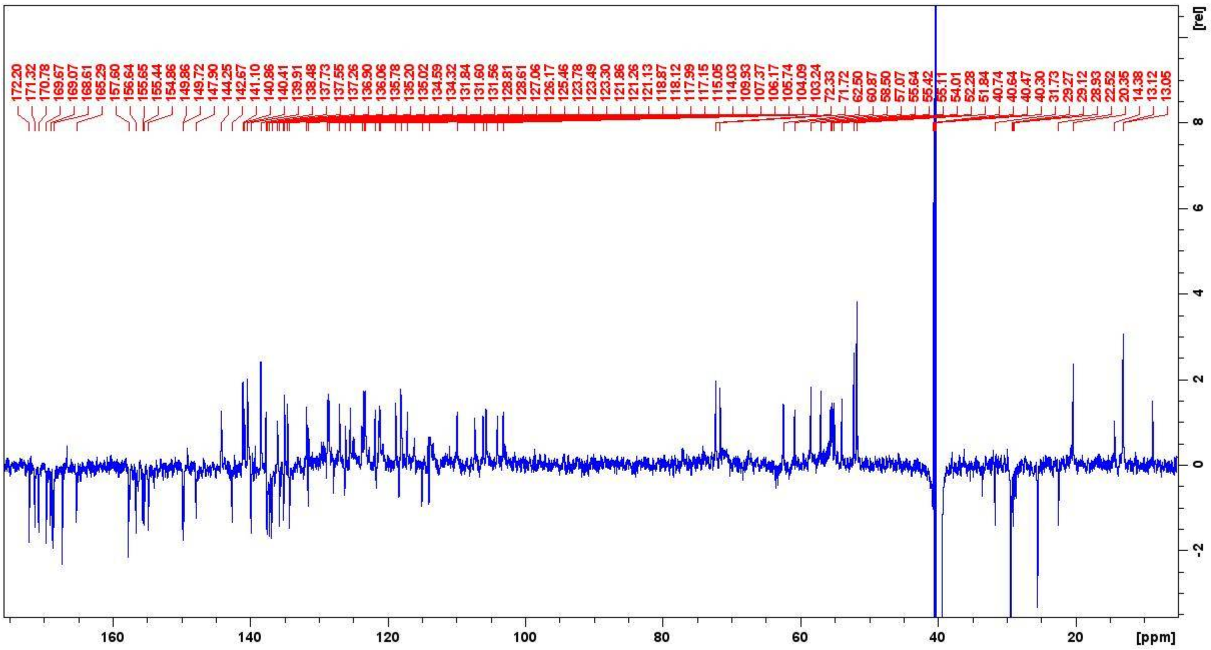

Supplement: Supplementary file 1 [file pharmaceuticals-14-01111-s001.zip › pharmaceuticals-1426331-supplementary.pdf]
